# Organ-Specific Analysis of *Morus alba* Using a Gel-Free/Label-Free Proteomic Technique

**DOI:** 10.3390/ijms20020365

**Published:** 2019-01-16

**Authors:** Wei Zhu, Zhuoheng Zhong, Shengzhi Liu, Bingxian Yang, Setsuko Komatsu, Zhiwei Ge, Jingkui Tian

**Affiliations:** 1 College of Biomedical Engineering and Instrument Science, Zhejiang University, Hangzhou 310027, China; rutin@zju.edu.cn (W.Z.); zhongzhh@zju.edu.cn (Z.Z.); bylzs8410@163.com (S.L.); xianyb@zju.edu.cn (B.Y.); 2 Faculty of Environmental and Information Sciences, Fukui University of Technology, Fukui 910-8505, Japan; skomatsu@fukui-ut.ac.jp; 3 Analysis Center of Agrobiology and Environmental Sciences, Zhejiang University, Hangzhou 310027, China; gezw@zju.edu.cn; 4 Key Laboratory for Biomedical Engineering of Ministry of Education, Zhejiang-Malaysia Joint Research Center for Traditional Medicine, Zhejiang University, Hangzhou 310027, China

**Keywords:** *Morus*, organ, gel-free/label-free proteomics, flavonoid, antioxidant activity

## Abstract

*Morus alba* is an important medicinal plant that is used to treat human diseases. The leaf, branch, and root of *Morus* can be applied as antidiabetic, antioxidant, and anti-inflammatory medicines, respectively. To explore the molecular mechanisms underlying the various pharmacological functions within different parts of *Morus*, organ-specific proteomics were performed. Protein profiles of the *Morus* leaf, branch, and root were determined using a gel-free/label-free proteomic technique. In the *Morus* leaf, branch, and root, a total of 492, 414, and 355 proteins were identified, respectively, including 84 common proteins. In leaf, the main function was related to protein degradation, photosynthesis, and redox ascorbate/glutathione metabolism. In branch, the main function was related to protein synthesis/degradation, stress, and redox ascorbate/glutathione metabolism. In root, the main function was related to protein synthesis/degradation, stress, and cell wall. Additionally, organ-specific metabolites and antioxidant activities were analyzed. These results revealed that flavonoids were highly accumulated in *Morus* root compared with the branch and leaf. Accordingly, two root-specific proteins named chalcone flavanone isomerase and flavonoid 3,5-hydroxylase were accumulated in the flavonoid pathway. Consistent with this finding, the content of the total flavonoids was higher in root compared to those detected in branch and leaf. These results suggest that the flavonoids in *Morus* root might be responsible for its biological activity and the root is the main part for flavonoid biosynthesis in *Morus*.

## 1. Introduction

Mulberry tree (*Morus alba* L.) is a deciduous woody shrub in the family Moraceae and widely cultivated in China, Korea, India, and Japan [1]. In addition to its use in sericulture, *Morus* can be used in fruit production, tolerating saline soils, and soil retention in loess soils [2,3]. In China, different parts of the mulberry tree have a long history of being used in traditional Chinese medicine to treat human diseases such as diabetes, arthritis, and rheumatism [4]. Therefore, *Morus* has attracted attention for its pharmaceutical value. For example, the mulberry leaf has been proven to modulate the cardiovascular system through endothelial nitric oxide synthase signaling [5] and mulberry-leaf polysaccharides, which are one of the main active components in mulberry leaf, have been purified from an ethanol extraction and showed potential antioxidative activities [6]. The mulberry-branch bark had a powerful antidiabetic effect that could rescue gluconeogenesis and glycogen synthesis by protecting genes in the phosphatidylinositol-3 kinase and protein kinase B signaling pathways [7]. Furthermore, the ethanol extraction of mulberry root bark could effectively ameliorate hyperlipidemia and four major active compounds, including mulberrofuran C, sanggenon G, moracin O, and moracin P, were isolated [8]. However, the mechanisms for the different biological activity in different parts of *Morus* remain unclear.

For most medicinal plants, their pharmaceutical value varies in different parts of the plant. For example, the *Scutellaria baicalensis* root rather than its aerial parts can be used as a traditional Chinese medicine for its anticancer, anti-HIV, and antibacterial effects [9]. The flower from *Coreopsis tinctorial* has been reported to possess antioxidative and antidiabetic activities and is used as a health food, while its stems and leaves are commonly discarded [10]. Organ- and tissue-specific studies are an effective way to discover the reason for these phenomena and aid in the quality control and pharmacological evaluation of medicinal plants, especially for medicinal plants such as *Morus* whose vegetative organs (root, branch, and leaf) can all be used as medicines. A comparative study on the antioxidant activity and phenolic contents of methanol extractions from mulberry leaf, stem bark, fruit, and root bark indicated that the mulberry stem bark had the highest antioxidant activity [11]. In contrast, the ethanol extraction from mulberry leaf had a higher antioxidant activity than the fruit and stem extractions [12]. Furthermore, the antioxidant activity of the ethanolic extraction from mulberry twigs was better than that from mulberry root bark [13]. However, there have been no systemic studies comparing the antioxidant activities among mulberry root, branch, and leaf, and the mechanisms of their different antioxidant activities are still unknown.

Omics technologies allow for the analysis of the complete set of genomes and are the most popular approaches for performing systemic studies [14]. The majority of functional genomics is based on transcriptomics, proteomics and metabolomics [15]. Proteomic technologies provide one of the best choices for the functional analysis of translated parts of the genome and have been applied in organ-/tissue-specific studies in plants. Zhu et al. [16] performed organ-specific proteomic analysis on the medicinal plant *Mahonia* and successfully identified the root-specific expressed proteins S-adenosylmethionine synthetase and (S)-tetrahydroprotoberberine. As the roots accumulated the most alkaloids, such as columbamine, jatrorrhizine, palmatine, tetrandrine, and berberine, these proteins are assumed to be involved in alkaloid biosynthesis. Ji et al. [17] conducted a comparative proteomics analysis using healthy and infected leaves from *Morus* to study the dwarf response mechanism. Therefore, proteomic approaches can provide significantly more detailed protein information in different organs, which may be beneficial for revealing important mechanisms.

Although the leaf, branch, and root of *Morus* can all be used as traditional Chinese medicine, additional studies are needed to identify the organ-specific factors with medicinal value. In this study, to uncover the molecular mechanisms of the different pharmacological functions in *Morus* leaf, branch, and root, gel-free/label-free proteomic approach was used. Bioinformatic, phytochemical, and qRT-PCR techniques were used for confirmation of results from proteomics.

## 2. Results

### 2.1. The Metabolite Contents and Antioxidant Activity in Morus Root were Higher than in Branch and Leaf

**Figure 1:**
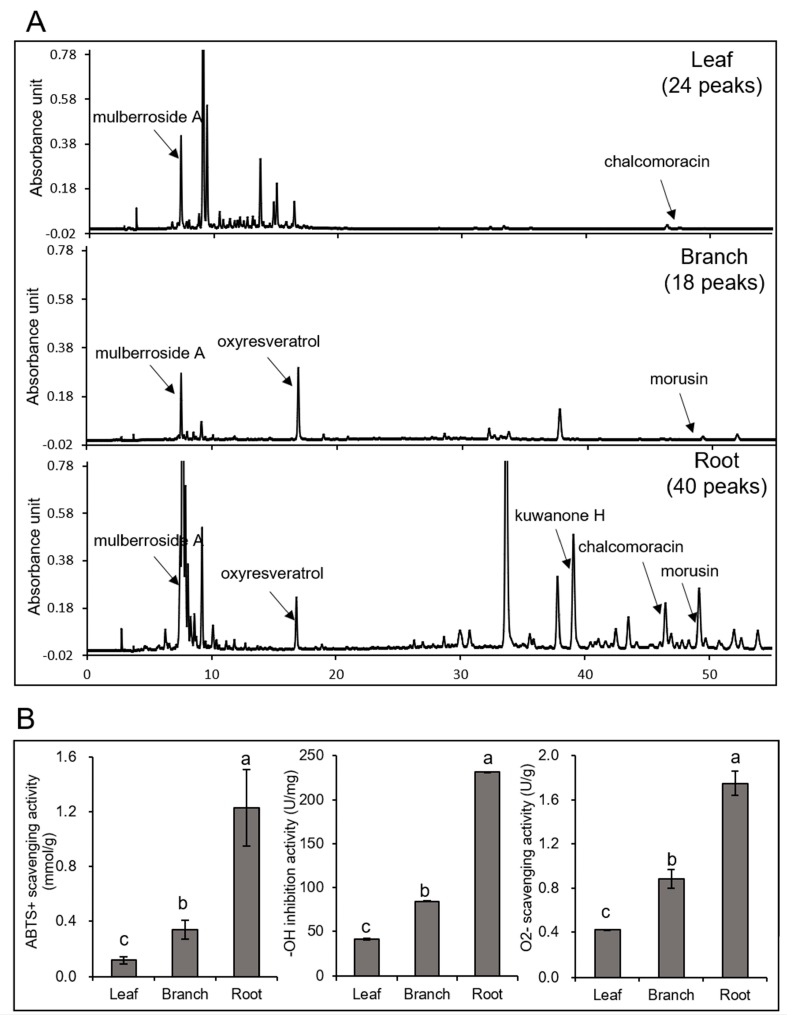
Analysis of the metabolites in *Morus* leaf, branch, and root and their antioxidant activities. The methanol extracts from *Morus* leaf, branch, and root were analyzed by HPLC (**A**). A C18 column was used with a flow rate of 1 mL min^−1^. The peaks were determined at a wavelength of 320 nm. For the determination of the antioxidant activities of *Morus* leaf, branch, and root, ABTS^+^ scavenging, hydroxyl free radical, and O_2_^−^ scavenging activities were analyzed (**B**). The data are shown as the mean ± SD from three independent biological replicates. Means with the same letter are not significantly different according to the one-way ANOVA test (*p* < 0.05).

To reveal differences among the leaf, branch, and root in *Morus*, five secondary metabolites were identified in the three organs. The leaf, branch and root were collected from mulberry trees and methanol extracts from the three organs were examined by HPLC analysis. From the HPLC chromatograms detected at 320 nm, 24, 18, and 40 peaks were observed in the extracts from the leaf, branch, and root, respectively (Figure 1A). Five major secondary metabolites including mulberroside A, oxyresveratrol, kuwanone H, chalcomoracin, and morusin were identified in the root, while only three of the five metabolites (mulberroside A, oxyresveratrol, and morusin) were detected in the branch and two of the five metabolites (mulberroside A, and chalcomoracin) were detected in the leaf. Except for oxyresveratrol, the contents of the other four metabolites in the root are more than 10 times higher than those in the leaf and branch (Table 1). To understand the biological differences among the three organs in Morus, the antioxidant activities were evaluated by using an ABTS+ scavenging activity, hydroxyl free radical, and O_2_^−^ scavenging activity assays. As a result, the root had the strongest ABTS+ scavenging activity, O_2_^−^ scavenging activity, and hydroxyl free radical inhibition activity, while the leaf had the worst antioxidant activity (Figure 1B). 

### 2.2. A Total of 257, 148, and 170 Proteins were Specific to Leaf, Branch, and Root in Morus, Respectively

**Figure 2:**
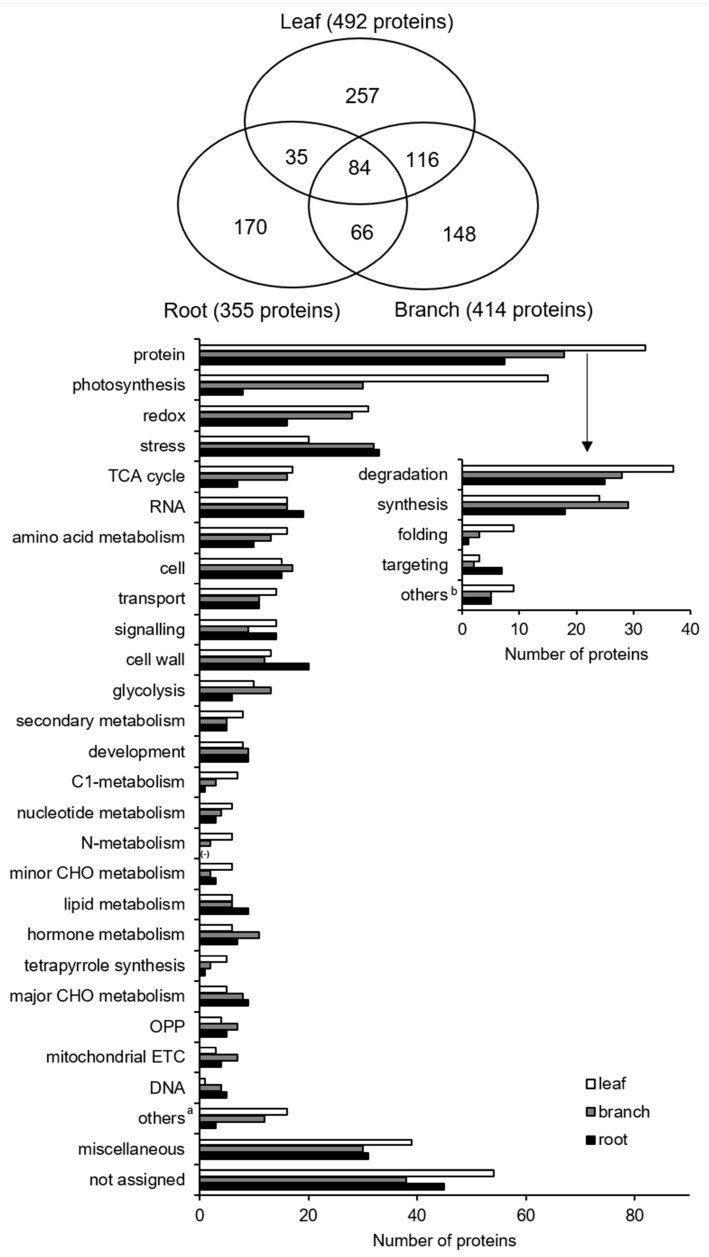
Functional categorization of proteins in leaf, branch, and root from *Morus*. Leaf (white), branch (gray), and root (black) samples were collected and proteins were extracted, digested, and analyzed by nanoLC-MS/MS. Protein functions were predicted and categorized using MapMan bin codes. Abbreviations: redox, redox ascorbate/glutathione metabolism; TCA, tricarboxylic acid; RNA, RNA processing and regulation of transcription; cell, cell organization, and vesicle transport; CHO, carbohydrates; OPP, oxidative pentose phosphate; ETC, electron transport chains; and DNA, DNA synthesis, and repair. ^a^ Others, containing biodegradation of xenobiotics, co-factor and vitamin metabolism, S-assimilation, gluconeogenesis, fermentation, and metal handling. ^b^ Others, containing amino acid activation, posttranslational modification, and assembly/cofactor ligation.

To analyze the mechanisms functioning within the three organs, a gel-free proteomics approach was used to identify and determine the abundance of proteins in mulberry leaf, branch, and root with the help of nano LC-MS/MS. A total of 492, 414, and 355 proteins with more than two matched peptides were identified in the leaf (Supplemental Table S1), branch (Supplemental Table S2) and root (Supplemental Table S3), respectively. Among these proteins, 257 (257/492, 52%), 148 (148/414, 36%), and 170 (170/355, 48%) were specific to the leaf, branch, and root, respectively (Figure 2).

To determine the biological processes involved in the three organs, the identified proteins were functionally classified using MapMan bin codes (Figure 2, Supplemental Tables S1–S3, Supplemental Figure S4. In leaf, the main functional categories were related to protein metabolism (82/492, 17%), photosynthesis (64/492, 13%), redox ascorbate/glutathione metabolism (31/492, 6%), stress (20/492, 4%), and tricarboxylic acid cycle (TCA, 17/492, 3%). In branch, the main functional categories were related to protein metabolism (67/414, 16%), stress (32/414, 8%), photosynthesis (30/414, 7%), redox ascorbate/glutathione metabolism (28/414, 7%), and cell cycle/cell organization and division (17/414, 4%). In root, the main functional categories were related to protein metabolism (56/355, 16%), stress (33/355, 9%), cell wall (20/355, 6%), RNA metabolism (19/355, 5%), and redox ascorbate/glutathione metabolism (16/355, 5%). Based on these results, proteins related to protein synthesis/degradation comprised the main functional category in all three organs. The abundance of proteins related to redox, TCA cycle, and glycolysis largely differed in the root samples compared to the leaf and branch samples (Figure 2).

### 2.3. Functional Characterization of Organ-Specific Proteins Identified from Morus

**Figure 3:**
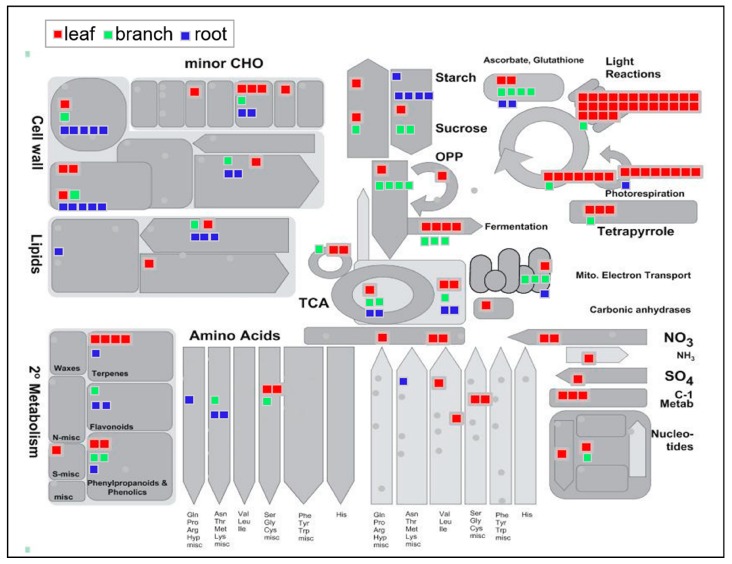
Organ-specific proteins were functionally categorized and visualized using the MapMan software. Leaf- (red), branch- (green), and root-(blue) specific proteins were submitted to the MapMan software (version 3.6.0RC1) using the metabolism overview pathway map. Each square indicates one mapped protein.

To determine the organ-specific protein expression patterns within each organ, leaf-, branch-, and root-specific proteins were functionally categorized and visualized using the MapMan software (version 3.6.0RC1, Aachen, Germany) (Figure 3). In the root, the number of cell wall-related proteins (10 proteins) was larger than in the leaf (four proteins) and branch (two proteins). Moreover, the proteins related to lipid metabolism identified in the root (four proteins) are more than the other organs (one protein for branch and two proteins for leaf). Additionally, five root-specific proteins related to major carbohydrate metabolism are functionally categorized as the degradation of sucrose and starch, while two leaf-specific proteins are involved in the synthesis of sucrose. In amino acid synthesis/metabolism, four root-specific proteins were found to be involved with amino acids; these belonged to the glutamate and aspartate families. There were also four leaf-specific proteins related to amino acid degradation/metabolism.

**Figure 4:**
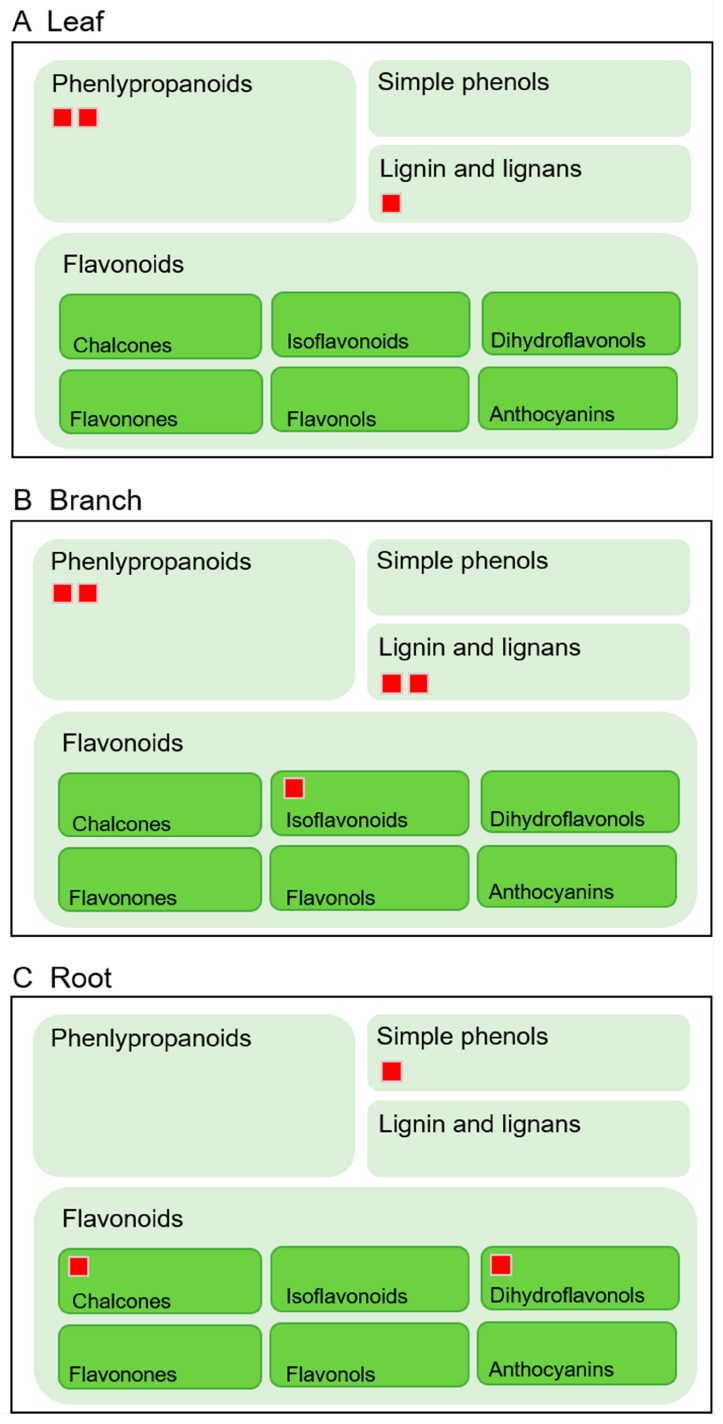
Comparison of the organ-specific proteins related to the flavonoid, phenlypropanoid, simple phenol, and lignin. Leaf- (**A**), branch- (**B**), and root- (**C**) specific proteins related to secondary metabolism were submitted to the MapMan software (version 3.6.0RC1). Each red square indicates one mapped protein.

There were several specifically identified proteins mapped to secondary metabolism that exhibited organ-specific expression patterns. Therefore, the organ-specific proteins involved in secondary metabolism were visualized using the MapMan software (version 3.6.0RC1, Aachen, Germany) (Supplemental Figure S1). There were eight, five, and four specifically identified proteins from the leaf, branch, and root samples, respectively. Leaf-specific proteins related to secondary metabolism mainly accumulated in the non-MVA and phenlypropanoid pathways. Branch-specific proteins related to secondary metabolism mainly accumulated in the phenlypropanoid and lignin/lignan pathways. Notably, the root-specific proteins related to secondary metabolism, such as chalcone flavanone isomerase and flavonoid 3,5-hydroxylase, were accumulated in the flavonoid pathway, especially for the biosynthesis of chalcones and dihydroflavonols (Figure 4).

### 2.4. Largely Differential Common Proteins Were Identified among Three Organs in Morus

To further analyze the differences in the proteins among the three organs, Venn diagram analysis was performed on the proteins identified in the three organs (Figure 2). A total of 84 proteins was commonly identified from the three organs, and the abundance of peroxidase (protein number 2) in the root was significantly higher than in the branch and leaf (Table 2). The abundance of proteasome (protein number 9) in the root was approximately six times higher than that found in the leaf. However, isoflavone reductase homolog P3 (protein number 48), which is the only protein involved in secondary metabolism from the commonly identified protein group, was the most abundant in the branch samples (1.17 mol%) compared to the leaf (0.33 mol%) and root (0.37 mol%) samples. Furthermore, the abundance of triosephosphate isomerase in the leaf is approximately ten times higher than in the root.

### 2.5. TCA Cycle and Glycolysis Pathways Largely Differed among the Three Organs

**Figure 5:**
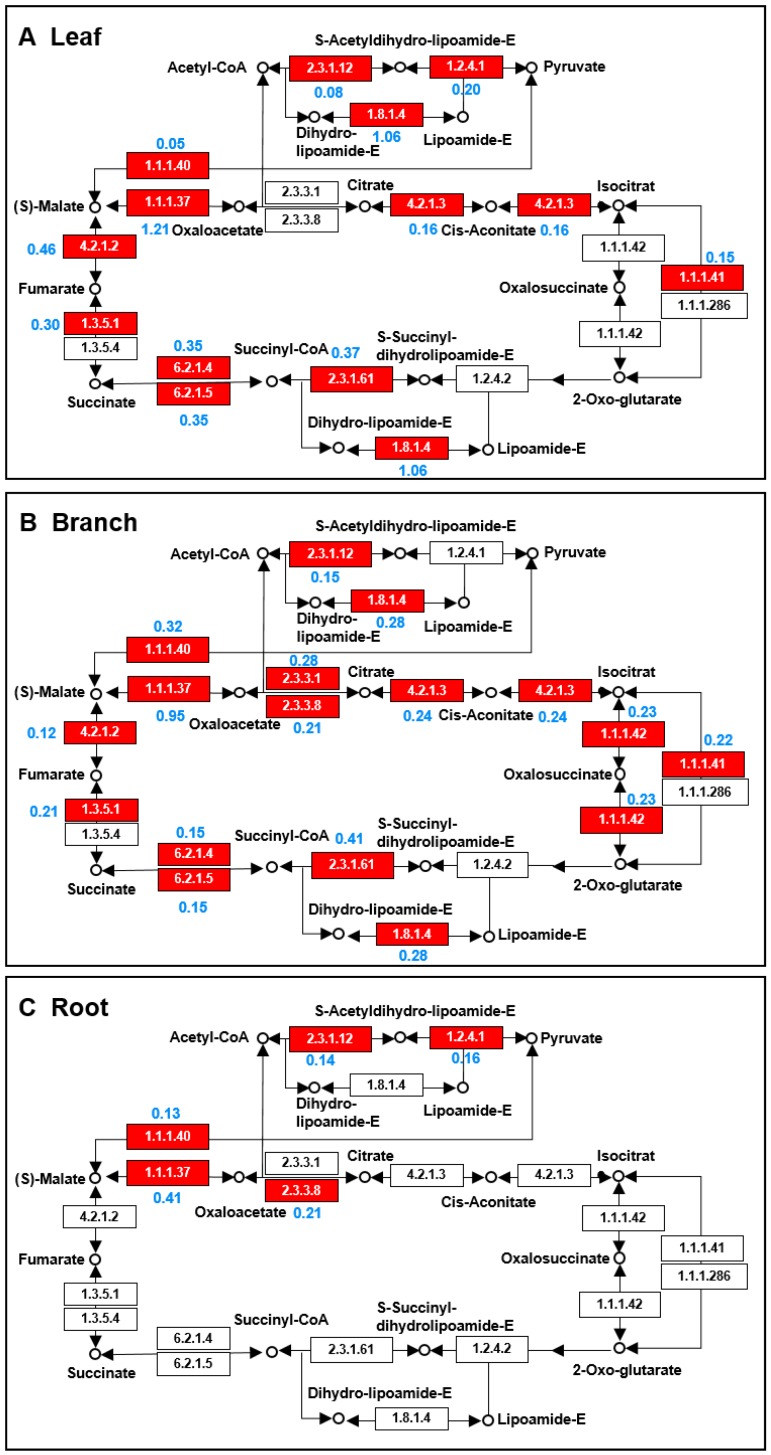
Mapping of proteins related to the TCA cycle in three organs from *Morus*. The TCA cycle pathways were identified by the mapping of the identified proteins from the leaf (**A**), branch (**B**), and root (**C**) using the KEGG database. Enzymes in red represent identified proteins, and the blue number represents the protein abundance. The EC number for the following proteins are 1.1.1.37, malate dehydrogenase; 1.1.1.40, malate dehydrogenase (oxaloacetate-decarboxylating)(NADP+); 1.1.1.41, isocitrate dehydrogenase (NAD+); 1.1.1.42, isocitrate dehydrogenase; 1.1.1.286, homoisocitrate dehydrogenase; 1.2.4.1, pyruvate dehydrogenase E1; 1.2.4.2, 2-oxoglutarate dehydrogenase; 1.3.5.1, succinate dehydrogenase; 1.3.5.4, fumarate reductase; 1.8.1.4, dihydrolipoamide dehydrogenase; 2.3.3.1, citrate synthase; 2.3.3.8, ATP-citrate synthase; 2.3.1.12, pyruvate dehydrogenase; 2.3.1.61, dihydrolipoamide succinyltransferase; 4.2.1.2, fumarate hydratase; 4.2.1.3, aconitate hydratase; 6.2.1.4, succinyl-CoA synthetase; and 6.2.1.5, ATP-citrate synthase.

**Figure 6:**
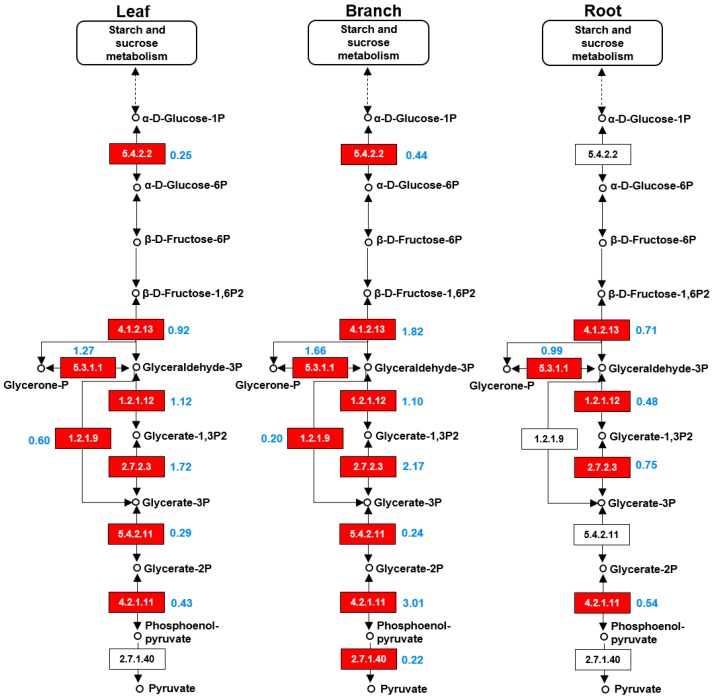
Mapping of proteins related to glycolysis in the three organs from *Morus*. The glycolysis pathways were identified by the mapping of the identified proteins from the leaf (**A**), branch (**B**), and root (**C**) using the KEGG database. Enzymes in red represent identified proteins, and the blue number represents the protein abundance. The EC number for the following proteins are 5.4.2.2, phosphoglucomutase; 4.1.2.13, fructose-bisphosphate aldolase; 5.3.1.1, triosephosphate isomerase; 1.2.1.12, glyceraldehyde 3-phosphate dehydrogenase; 1.2.1.9, glyceraldehyde-3-phosphate dehydrogenase (NADP); 2.7.2.3, phosphoglycerate kinase; 5.4.2.11, phosphoglycerate mutase; 4.2.1.11, enolase; and 2.7.1.40, pyruvate kinase.

To better understand the different metabolic pathways that are active in the leaf, branch, and root of *Morus*, the identified proteins from each organ were mapped to the TCA cycle and glycolysis pathways using the KEGG database (Figure 5 and Figure 6). In the TCA cycle, the abundance of dihydrolipoyl dehydrogenase (EC1.8.1.4) in the leaves was higher than that in the branches, and no dihydrolipoyl dehydrogenase was detected in the roots. The abundance of NADP-dependent malic enzyme (EC1.1.1.40) in the branches was higher than in the leaves and roots. The abundance of malate dehydrogenase (EC1.1.1.37) in the leaves was nearly three times greater than that in the roots. The abundance fumarate hydratase (EC4.2.1.2) in the leaves was more than three times higher than that in the branches. Furthermore, isocitrate dehydrogenase (EC1.1.1.42) was only identified in the branches (Figure 5). In the glycolysis pathway, the abundance of fructose bisphosphate aldolase (EC4.1.2.13), phosphoglycerate kinase (EC2.7.2.3), and enolase (EC4.2.1.11) in the roots were higher than in the branches and roots. Additionally, pyruvate kinase (EC2.7.1.40) was only identified in the branches (Figure 6).

### 2.6. Total Flavonoid Contents were the Highest in the Roots

**Figure 7:**
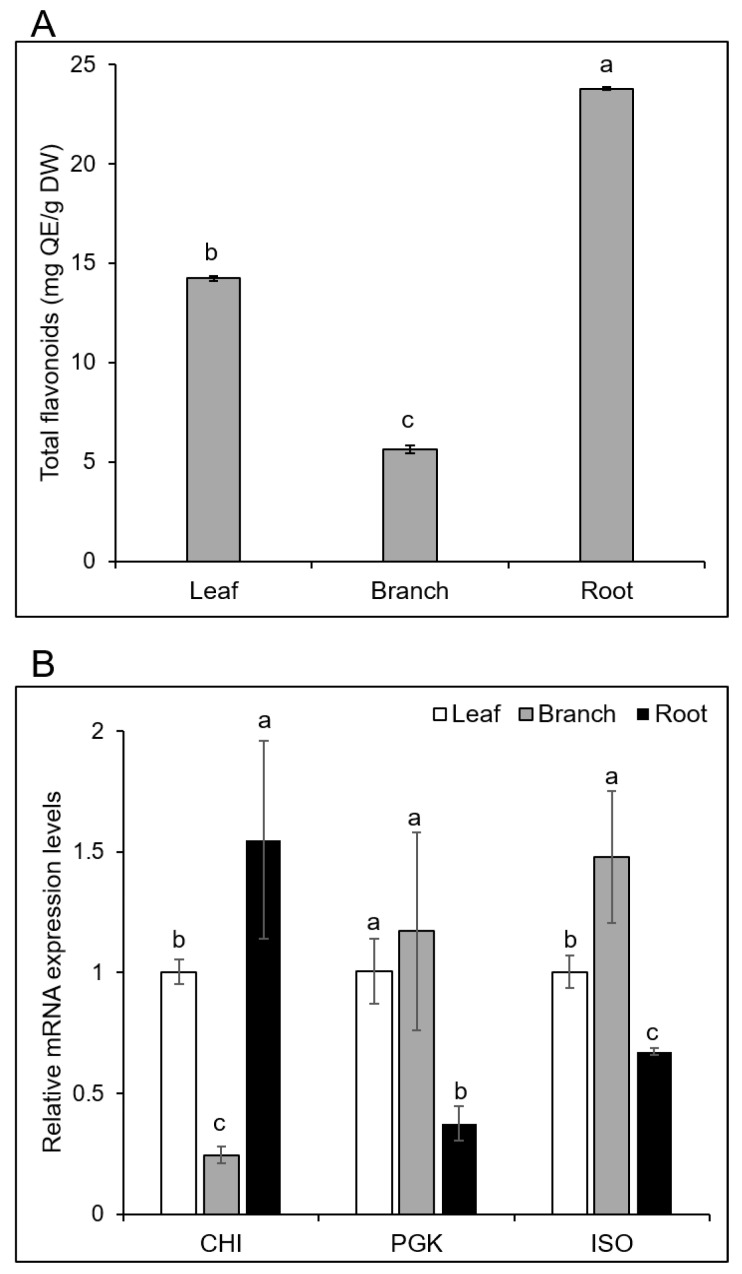
Total flavonoid contents and the expression of three genes in the three organs from *Morus*. The total flavonoids extracted from *Morus* leaf, branch, and root were analyzed using a colorimetric method (**A**). The transcript abundance of the selected genes was analyzed by qRT-PCR. Total RNA was extracted from the collected leaf, branch, and root (**B**). Data are shown as the means ± SD from three independent biological replicates. Means with the same letter are not significantly different according to one-way ANOVA test (*p* < 0.05). The abbreviations are follows: QE, quercetin-3-O-rutinoside. The genes CHI, PGK, and ISO represent chalcone flavanone isomerase, phosphoglycerate kinase, and isoflavone reductase, respectively. The *β*-actin gene was used as a reference control gene.

The proteomics analysis of *Morus* revealed that two proteins involved in the flavonoid biosynthetic pathway were only identified in the roots (Figure 4). Based on this finding, the total flavonoid contents of the three organs in *Morus* were analyzed to confirm whether the flavonoids accumulated in the roots. The total flavonoids extracted from the leaves, branches, and roots of *Morus* were analyzed using a colorimetric method. The results confirmed that the total flavonoid contents in the roots were significantly higher than in the leaves and branches (Figure 7A); the levels were approximately 1.5 times higher than in the leaves and four times higher than in the branches (Supplemental Table S4).

### 2.7. Expression of Genes Related to Root- and Branch-Specific Proteins

Functional characterization of the root-specific proteins showed that chalcone flavanone isomerase, which is involved in secondary metabolism, was accumulated through the flavonoid pathway (Figure 4). The abundance of isoflavone reductase in the branch was higher than in the leaf and root (Table 2). Moreover, the abundance of phosphoglycerate kinase in the branch, which is involved in the glycolysis pathway, was higher than in the other organs (Figure 6). Based on this finding, these three genes, chalcone flavanone isomerase (CHI), isoflavone reductase (ISO), and phosphoglycerate kinase (PGK), were selected for further analysis of their mRNA expression levels (Figure 7B). Among the examined genes, the mRNA expression of CHI in the root is significantly higher than in the leaf and branch. Additionally, the expression of PGK and ISO in the branch is significantly higher than in the root (Figure 7B).

## 3. Discussion

### 3.1. The Secondary Metabolites and Total Flavonoid Contents are Different in Morus Leaf, Branch, and Root

*Morus* is a widely distributed medicinal plant in China, and various parts of *Morus* are commonly used in traditional Chinese medicinal treatments [4]. To date, it has been reported that the leaf, branch, and root of *Morus* have various pharmacological activities, such as antidiabetic, anti-inflammatory, and anticancer [18,19,20]. In this study, the antioxidant activity of the root was the best among the three *Morus* organs examined (Figure 1B). It is known that the antioxidant-enzyme system is an important part of plant responses to oxidative stress [21]. Among the antioxidant enzymes, superoxide dismutase (SOD) and peroxidase (POD) play a key role in the antioxidant defense mechanism [22]. SOD is the first line of defense in the enzymatic pathway against free oxygen radicals [23]. POD is an enzymatic protectant that scavenges both radical and non-radical oxygen species [24]. In the present study, the abundance of peroxidase and superoxide dismutase were higher in the root than in the other organs (Table 2), which might contribute to high antioxidant activity of the *Morus* root.

In contrast, many secondary metabolites with various biological activities, such as alkaloids, flavonoids, polysaccharides, terpenoids, phenolic acids, stilbenoids, and coumarins, were identified in *Morus* [25]. Chen et al. [26] compared the chemical composition among the bark, leaf, twig, and fruit of *Morus*, and further analysis showed that the bark contained the highest amount of prenylated flavonoids (kuwanon G, sanggenon C, morusin, and mulberroside A) compared to the twig, leaf, and fruit. The total flavonoid contents showed a significant contribution to α-glucosidase inhibition. In the present study, the differences among the leaf, branch, and root from *Morus* were uncovered at the protein level using a gel-free proteomics technique. Two proteins (chalcone flavanone isomerase and flavonoid 3,5-hydroxylase) involved in the flavonoid biosynthetic pathway were only identified in the root (Figure 4), which might mean that synthesized flavonoids accumulate in the root. 

Chalcone flavanone isomerase (CHI) is a key branch-point enzyme between the phenylpropanoid and flavonoid pathways that can catalyze the synthesis of flavanones and the backbone for many downstream metabolites including flavonoids and isoflavonoids [27]. Flavonoid 3,5-hydroxylase (F3’,5’Hs), which belongs to the cytochrome P450 (CYP) enzyme family [28], can catalyze the hydroxylation of the flavonoid B-ring at the 3’ and 5’ positions [29]. The results also indicated that dihydroxy B-ring-substituted flavonoids have a great potential to inhibit the generation of ROS and show antioxidant activity [30]. In the present study, the total flavonoid and five secondary metabolites contents were highly accumulated in the roots of *Morus* (Figure 1). This evidence strengthens the idea that both antioxidant enzymes and secondary metabolites in the root of *Morus* are responsible for the antioxidant activity.

### 3.2. Anaylses of Enzymes Involved in the Glycolysis and Isoflavonoid Biosynthetic Pathway in Morus

Glycolysis is a sequence of ten enzyme-catalyzed reactions that converts glucose into pyruvate. Proteomic analyses of leaf, branch, and root showed that proteins related to glycolysis were mostly abundant in the branch compared with the leaf and root (Figure 6). The abundance of fructose-bisphosphate aldolase (EC:4.1.2.13), phosphoglycerate kinase (EC:2.7.2.3), and enolase (EC4.2.1.11) in the branch was higher than in the leaf and root samples (Table 2). These enzymes play important roles in glycolysis. Fructose-bisphosphate aldolase catalyzes the split of fructose 1,6-bisphosphate into dihydroxyacetone phosphate and glyceraldehyde 3-phosphate [31]. Phosphoglycerate kinase catalyzes the transfer of a phosphate group from 1,3-bisphosphoglycerate to ADP via phosphoglycerate kinase, forming ATP and 3-phosphoglycerate [32]. Enolase converts 2-phosphoglycerate to phosphoenolpyruvate [33]. Cramer et al. [34] reported that the early-responding proteins to water deficit included proteins related to photosynthesis, glycolysis, translation, antioxidants, and growth, which could funnel carbon and energy into antioxidant defenses during the very early stages of plant responses to water deficit before any significant injury. The enhanced glycolysis in the mulberry branch might be engaged in a similar regulatory pathway, which enables the branch to exhibit antioxidant activity in vitro. 

Furthermore, mulberry root bark is usually used in traditional Chinese medicine as a diuretic and expectorant agent, while the leaf was consumed as food by silkworms [35] and the fruit was taken as a health food [36]. However, the branch was largely neglected and ended up as fire wood material or agro-waste. Few studies have examined and confirmed the pharmacological activities of the branch bark from mulberry [37,38]. When comparing the abundance of commonly identified proteins from different parts of *Morus*, we discovered that isoflavone reductase homolog (IFRh) was most abundant in branches, approximately three times higher than in the leaf and root. Isoflavone reductase (IFR) is located in the cytoplasm and has been identified as one of the key enzymes involved in the synthesis of isoflavonoid phytoalexin [39,40,41]. IFR is unique to the plant kingdom and considered to have crucial roles in plant responses to various biotic and abiotic stresses. Cheng et al [42] discovered that overexpression of soybean isoflavone reductase enhanced resistance to *Phytophthora sojae* in soybean, though its specific biological function remains to be elucidated. The abundantly expressed isoflavone reductase homolog protein in mulberry branch inspired us to propose that intense isoflavonoid biosynthesis is present in the mulberry branch. However, the reason for this phenomenon is unknown and the function of IFRh in *Morus* is worth further investigation. 

## 4. Materials and Methods 

### 4.1. Plant Materials and Growth Conditions

Mulberry trees (*Morus alba* L.) were provided by the College of Agriculture and Biotechnology, Zhejiang University (Hangzhou, China). They were grown in a greenhouse under white fluorescent light (160 μmol m^−2^ s^−1^, 16 h light period/day) at 25 °C and 70% humidity. The soil conditions were controlled to ensure normal plant growth without exposure to extreme drought or plant diseases. Leaf, branch and root were then collected, frozen in liquid nitrogen and stored at −80 °C. For each organ, three independent experiments were performed as biological replicates. Each biological replicate means leaves, branches, and roots from three individuals were collected and analyzed by mass spectrometry separately. A total of nine plants were used in this study (Supplemental Figures S2 and S3).

### 4.2. Protein Extraction

A portion (0.5 g) of each organ sample was ground into powder in liquid nitrogen using a mortar and pestle and then transferred into a polypropylene tube containing a solution of 10% trichloroacetic acid and 0.07% 2-mercaptoethanol in acetone. The resulting mixture was vortexed and sonicated for 10 min at 4 °C. The suspension was incubated for 1 h at −20 °C with vortexing every 15 min. It was then centrifuged at 9000× *g* for 10 min at 4 °C. The supernatant was discarded and the pellet was washed twice with 0.07% 2-mercaptoethanol in acetone. The final pellet was dried and resuspended in lysis buffer consisting of 7 M urea, 2 M thiourea, 5% CHAPS, and 2 mM tributylphosphine by vortexing for 1 h at 25 °C. The suspension was then centrifuged at 20,000× *g* for 20 min at room temperature until a clean supernatant was obtained. Protein concentrations were determined using the Bradford assay [43] with bovine serum albumin as the standard.

### 4.3. Purification and Digestion of Proteins for Mass Spectrometry Analysis

Proteins (100 μg) were purified with methanol and chloroform to remove any detergent from the sample solutions [44]. Briefly, 400 μL of methanol was added to each sample, and the resulting solution was mixed. Subsequently, 100 μL of chloroform and 300 μL of water were added to each sample, which were mixed and centrifuged at 20,000× *g* for 10 min to achieve phase separation. The upper aqueous phase was discarded and the pellets were dried. The dried pellets were resuspended in 50 mM NH_4_HCO_3_. The proteins were reduced with 50 mM dithiothreitol for 30 min at 56 °C and alkylated with 50 mM iodoacetamide for 30 min at 37 °C in the dark. Alkylated proteins were digested with trypsin and lysyl endopeptidase (Wako, Osaka, Japan) at 1:100 enzyme/protein concentrations at 37 °C for 16 h. The resulting tryptic peptides were acidified by mixing with formic acid (pH < 3), and the resulting solution was centrifuged at 20,000× *g* for 10 min. The obtained supernatant was collected and analyzed by nanoliquid chromatography (LC)- mass spectrometry (MS).

### 4.4. Nanoliquid Chromatography-Tandem Mass Spectrometry Analysis

Peptides were analyzed using a nanospray LTQ Orbitrap mass spectrometer (Thermo Fisher Scientific, San Jose, CA, USA) with the Xcalibur software (version 2.1, Thermo Fisher Scientific, Bremen, Germany) in data-dependent acquisition mode. Using an Ultimate 3000 nanoLC system (Dionex, Germering, Germany), peptides in 0.1% formic acid were loaded onto a C18 PepMap trap column (300 μm ID × 5 mm, Dionex, Sunnyvale, CA, USA) and were then eluted with a linear acetonitrile gradient (8–30% over 150 min) in 0.1% formic acid at a flow rate of 200 nL/min. The eluted peptides were separated and sprayed on a C18 capillary tip column (75 μm ID × 120 mm, Nikkyo Technos, Tokyo, Japan) with a spray voltage of 1.5 kV.

Full-scan mass spectra were acquired on the LTQ Orbitrap mass spectrometer (Thermo Fisher Scientific, San Jose, CA, USA) over 400–1500 m/z with a resolution of 30,000. A lock mass function was used for high mass accuracy [45]. The ten most intense precursor ions were selected for collision-induced fragmentation in the linear ion trap at a normalized collision energy of 35%. Dynamic exclusion was employed within 90 s to prevent the repetitive selection of peptides [46].

### 4.5. Protein Identification from the Mass Spectrometry Data

Proteins were identified using the Mascot search engine (version 2.5.1; Matrix Science, London, UK) with the *Morus* Genome database (MorusDB) (version 2.0, https://morus.swu.edu.cn/morusdb/datasets). The acquired raw data files were processed using Proteome Discoverer (version 1.4.0.288, Thermo Fisher Scientific, Bremen, Germany). The parameters used in Mascot searches were as follows: carbamidomethylation of cysteine was set as a fixed modification and oxidation of methionine was set as a variable modification. Trypsin was specified as the proteolytic enzyme, and one missed cleavage was allowed. The peptide mass tolerance was set at 10 ppm, the fragment mass tolerance was set at 0.8 Da, and the peptide charge was set at +2, +3, and +4. An automatic decoy database search was performed as part of the search. Mascot results were filtered with Mascot Percolator to improve the accuracy and sensitivity of peptide identification [47]. The false discovery rates for peptide identification in all searches were less than 1.0%. Peptides with a percolator ion score of more than 13 (*p* < 0.05) were used for protein identification. Protein abundance was analyzed based on the exponentially modified protein abundance index (emPAI) value [48]. Mascot-derived emPAI values were converted to molar percentages by normalizing against the sum of all emPAI values for the acquisition. Briefly, the mean of three emPAI values was divided by the sum of the emPAI values for all identified proteins and multiplied by 100. The protein content was estimated by the molar fraction percentage (mol%).

### 4.6. Functional Analysis of Identified Proteins

Protein functions were categorized using MapMan bin codes as previously described [49]. The small-scale prediction of the identified proteins from Mahonia was performed by transferring annotations from the *Arabidopsis thaliana* genome and considering orthologous genes. Pathway mapping of identified proteins was performed using the Kyoto Encyclopedia of Genes and Genomes (KEGG) database (https://www.kegg.jp/) [50].

### 4.7. Quantitative Analysis of Metabolites from Morus

A portion (0.2 g dry weight) of collected organ sample was sonicated in 150 mL of methanol for 2 h, centrifuged at 10,000× *g* for 10 min, and the supernatant was collected. The methanol extracts were dried in a rotary evaporator at 50 °C. For root samples, the obtained residue was dissolved in 10 mL of methanol. For branch and leaf samples, the obtained residue was dissolved in 2 mL of methanol. The dissolved samples were then filtered through a 0.45-μm filter (Millipore, Bullerica, MA, USA) for HPLC analysis. Several standard compounds, which consisted of mulberroside A, oxyresveratrol, kuwanone H, chalcomoracin, and morusin, were provided by Zhejiang Institute for Food and Drug Control (Hangzhou, China). For quantification, a calibration curve was constructed using the standard solutions diluted in methanol at six different concentrations: mulberroside A (0.05, 0.1, 0.25, 0.375, 0.5, and 0.75 mg mL^−1^), oxyresveratrol (0.001, 0.005, 0.01, 0.025, 0.05, and 0.1 mg mL^−1^), kuwanone H (0.01, 0.05, 0.1, 0.2, 0.3, and 0.5 mg mL^−1^), chalcomoracin (2.36, 4.72, 9.44, 14.16, 18.88, and 23.6 mg mL^−1^), and morusin (0.01, 0.05, 0.1, 0.25, 0.5, and 1 mg mL^−1^). For HPLC analysis, 10 μL of the standard solutions and samples were used.

HPLC analysis was performed on a Waters 2695 Alliance HPLC system (Waters, Milford, MA, USA) equipped with a photodiode array detector, an online degasser and an auto-sampler for solvent delivery. Compounds in samples were separated using reverse-phase HPLC. A C18 column (4.6 mm ID × 250 mm, Agilent, Santa Clara, CA, USA) was used with a flow rate of 1 mL min^−1^ at 40 °C. The solvent system consisted of a linear gradient from 10% to 95% (*v*/*v*) acetonitrile in water with 0.1% phosphoric acid over a period of 70 min followed by an isocratic elution with 95% for 5 min. The spectra were measured at 320 nm and the retention time and ultraviolet spectra of the samples’ peaks were compared with that of the standards’ peaks.

### 4.8. In Vitro Antioxidant Activity Analysis of Mulberry Leaf, Branch, and Root

For the determination of the antioxidant activities of mulberry leaf, branch and root, ABTS^+^ scavenging activity, hydroxyl free radical and O_2_^−^ scavenging activity were analyses. The ABTS^+^ scavenging activities within different organs were determined using a total antioxidant capacity assay kit (A015-2, Nanjing Jiancheng Bioengineering Institute, Nanjing, China) following the manufacturer’s protocol. Briefly, 10 μL of each sample were mixed with diluted ABTS^+^ solution and then shaken vigorously for 6 min at room temperature in the dark. The absorbance of the samples was measured at 405 nm immediately after incubation. A calibration curve was constructed using absorbance values measured when ABTS^+^ solution was mixed with a standard antioxidant—Trolox at 0.1 mM, 0.2 mM, 0.4 mM, 0.8 mM and 1.0 mM. The antioxidant activity of each group was measured as a Trolox-Equivalent Antioxidant Capacity (TEAC).

The hydroxyl free radical assay was performed using kit A018 purchased from the Nanjing Jiancheng Bioengineering Institute following the manufacturer’s protocol based on the principle of the Fenton reaction [51]. Samples were mixed with reaction buffer and reacted at 37 °C for 1 min and terminated with the addition of developer. The absorbance of the samples was measured at 550 nm after developing for 20 min; the standard sample contained 0.03% H_2_O_2_ as a control. 

The O_2_^−^ scavenging activities were determined using the inhibition and produce superoxide anion assay kit (A052, Nanjing Jiancheng Bioengineering Institute, Nanjing, China) according to manufacturer’s protocol. Samples were mixed with reaction buffer, incubated at 37 °C for 40 min and terminated by the addition of developer. The absorbance of the samples was measured at 550 nm after developing for 10 min developing; a vitamin C standard solution at 0.15 mg mL^−1^ was used as the control.

### 4.9. RNA Extraction and Quantitative Reverse Transcription-Polymerase Chain Reaction Analysis

Samples (0.1 g fresh weight) were ground to powder in liquid nitrogen using a sterilized mortar and pestle. Total RNA was extracted using a Quick RNA Isolation Kit (Huayueyang Biotechnology, Beijing, China) and reverse-transcribed using a 5X All-In-One RT MasterMix with AccuRT Genomic DNA Removal Kit (Applied Biological Materials Ins, CA) according to manufacturers’ protocols. The primers were designed using Primer Premier 6.0. qRT-PCR was performed in a 10 μL reaction volume using an Evagreen 2 × qPCR MasterMix (Applied Biological Materials Ins, CA) in an IQ5 multicolor real-time PCR detection System (Bio-Rad, Hercules, CA, USA). The relative quantification method (2-ΔΔCT) was used to evaluate the quantitative variation between treatments. β-actin (GeneBank ID: HQ 163776) served as an internal control to normalize target gene quantities [52]. The gene-specific primers are listed in Supplemental Table S5. The qRT-PCR results were analyzed using the Statistical Product and Service Solutions software (version 20.0, IBM, Armonk, NY, USA).

### 4.10. Quantitative Analysis of the Total Flavonoids in Three Morus Organs

The total flavonoid contents of the three organs in *Morus* was determined by a colorimetric method as described previously with minor modifications [53]. Briefly, a portion (2.0 g) of each freeze-dried organ sample was sonicated in 100 mL of methanol for 30 min, centrifuged at 8000× *g* for 10 min, and the supernatant was collected. The pellet was resuspended in 100 mL of methanol and the resulting suspension was sonicated for 30 min. The methanol extracts were dried in a rotary evaporator at 50 °C. The obtained residue was dissolved in methanol using 10 mL in a volumetric flask and brought to volume by methanol. One milliliter of the final extract was placed in a 10 mL volumetric flask. Then, 0.5 mL of 5% NaNO_2_ was added and the mixture was maintained for 5 min at room temperature. After incubation, 0.5 mL of 10% Al (NO_3_)_3_ was added to the reaction mixture and incubated for 5 min. Next, 1.5 mL of 2 M NaOH was added and methanol was added up to volume. After incubating for 15 min, the absorbance was measured at 510 nm. The data are expressed as mg quercetin-3-O-rutinoside (rutin) equivalents (QE)/g dry weight (DW), as quercetin-3-O-rutinoside was used as a reference standard for the quantification of the total flavonoids.

### 4.11. Statistical Analysis

The SPSS statistical software (version 22.0, IBM, Armonk, NY, USA) was used for the statistical evaluation of the results. Statistical significance was evaluated by Student’s t-test when only two groups were compared and with one-way ANOVA test when multiple groups were compared. All results are presented as the mean ± SD from three independent biological replicates. A *p*-value less than 0.05 was considered statistically significant.

## 5. Conclusions

*Morus* is a medicinal plant with various biological activities. In this study, it is indicated that the five secondary metabolites, including mulberroside A, oxyresveratrol, kuwanone H, chalcomoracin, and morusin, and total flavonoids contents in *Morus* roots are higher than in other organs, which might be responsible for its highest antioxidant activity. Proteomic analysis of the leaf, branch, and root from *Morus* revealed that proteins related to the flavonoid pathway such as chalcone flavanone isomerase and flavonoid 3,5-hydroxylase were accumulated in the root, resulting in the highest total flavonoid contents among the three examined organs. Additionally, the protein expression profiling of the leaf, branch, and root in *Morus* will enrich the proteome database of *Morus*. Additionally, the present findings suggest that flavonoid biosynthesis is an important function in *Morus* root.

## Figures and Tables

**Table 1:** Contents of five secondary metabolites in different organs of *Morus*.

	Mulberroside A (mg/g DW)	Oxyresveratrol (mg/g DW)	Kuwanone H (mg/g DW)	Chalcomoracin (mg/g DW)	Morusin (mg/g DW)
**Leaf**	0.917 ± 0.015	n.d. *	n.d.	0.045 ± 0.005	n.d.
**Branch**	0.451 ± 0.012	0.453 ± 0.008	n.d.	n.d.	0.043 ± 0.008
**Root**	24.206 ± 0.688	0.345 ± 0.022	5.551 ± 0.226	0.610 ± 0.051	2.874 ± 0.158

* n.d. means the metabolites is not determined in this organ; DW, means dry weight.

**Table 2:** Common proteins identified in the leaf, branch, and root of *Morus* by gel-free/label-free proteomic analysis.

No.	Protein ID ^a^	Description	M.P. ^b^	Score	Mass (Da)	Function ^c^	Mol (%) ^d^		
							leaf	stem	root
1	Morus009492.p1	Macrophage migration inhibitory factor homolog	8	203	8961	not assigned	3.10	2.43	3.69
2	Morus001961.p1	Peroxidase 12	16	244	38,426	misc	0.57	1.29	3.62
3	Morus018316.p1	Superoxide dismutase 1 copper chaperone	9	339	11,171	metal handling	2.11	1.69	2.48
4	Morus009000.p1	60S acidic ribosomal protein P2B	12	110	11,673	protein	1.62	2.32	2.32
5	Morus023628.p1	Tubulin beta-1 chain	22	578	51,015	cell	1.11	1.83	1.99
6	Morus017847.p1	Ribonuclease UK114	16	266	19,960	RNA	1.08	0.27	1.80
7	Morus017207.p1	Proteasome subunit alpha type-4	6	173	27,440	protein	0.24	0.34	1.58
8	Morus003952.p1	Lipoxygenase homology domain-containing protein 1	6	154	21,171	not assigned	0.50	0.95	1.44
9	Morus022430.p1	Proteasome subunit beta type-1	11	313	24,861	protein	0.68	0.56	1.44
10	Morus022592.p1	Thaumatin-like protein 1a	18	362	26,984	stress	1.02	0.44	1.41
11	Morus022525.p1	Calmodulin	9	250	16,894	signaling	1.03	1.12	1.31
12	Morus015082.p1	Auxin-repressed 12.5 kDa protein	3	89	13,355	development	0.49	1.99	1.30
13	Morus003616.p1	Fructokinase-2	10	137	35,370	major CHO metabolism	0.42	0.79	1.25
14	Morus017382.p1	Calcium-binding protein CML27	4	116	18,705	signaling	0.26	0.29	1.05
15	Morus008669.p1	Allene oxide cyclase 2, chloroplastic	6	168	27,569	hormone metabolism	0.60	1.24	0.99
16	Morus010743.p1	Triosephosphate isomerase, cytosolic	19	263	27,548	glycolysis	1.27	1.66	0.99
17	Morus004210.p1	Glucan endo-1,3-beta-glucosidase, basic vacuolar isoform	20	178	39,002	misc	0.63	0.34	0.94
18	Morus011779.p1	Superoxide dismutase [Cu-Zn], chloroplastic	22	579	29,603	redox	0.74	0.31	0.90
19	Morus001936.p1	Peroxiredoxin-2B	17	350	17,391	redox	1.22	1.48	0.90
20	Morus004201.p1	Universal stress protein A-like protein	9	223	18,591	stress	0.58	0.58	0.82
21	Morus001634.p1	Nucleoside diphosphate kinase 1	11	215	16,322	nucleotide metabolism	1.35	0.78	0.78
22	Morus003013.p1	Phosphoglycerate kinase, cytosolic	59	1143	42,729	glycolysis	1.72	2.17	0.75
23	Morus028068.p1	Polygalacturonase inhibitor 1	10	306	37,677	cell wall	0.86	0.73	0.73
24	Morus013807.p1	Fructose-bisphosphate aldolase, cytoplasmic isozyme	23	584	38,459	glycolysis	0.92	1.82	0.71
25	Morus020532.p1	Glutaredoxin	5	244	15,307	redox	0.75	0.69	0.69
26	Morus023908.p1	Uncharacterized protein	15	271	57,888	protein	0.50	1.55	0.69
27	Morus018475.p1	Peroxidase 54	5	128	36,921	misc	0.13	0.47	0.67
28	Morus025517.p1	Tubulin alpha chain	18	426	49,920	cell	0.68	0.72	0.66
29	Morus002489.p1	Nascent polypeptide-associated complex subunit alpha-like protein 1	11	285	22,279	protein	1.01	0.34	0.65
30	Morus014304.p1	Plastocyanin, chloroplastic	23	661	16,620	photosynthesis	1.16	0.62	0.62
31	Morus024265.p1	Aquaporin PIP1-3	5	67	30,856	transport	0.22	0.37	0.59
32	Morus008884.p1	Cysteine proteinase RD21a	10	350	52,217	protein	0.35	0.32	0.58
33	Morus025862.p1	ATP synthase subunit beta, mitochondrial	33	1017	59,400	mitochondrial electron transport	1.06	1.43	0.54
34	Morus026982.p1	Allene oxide synthase, chloroplastic	11	164	56,861	hormone metabolism	0.30	0.91	0.54
35	Morus007342.p1	Peroxiredoxin-2F, mitochondrial	12	178	22,580	redox	0.77	1.03	0.52
36	Morus009738.p1	ATP-dependent Clp protease proteolytic subunit 5, chloroplastic	4	146	34,203	protein	0.13	0.21	0.52
37	Morus009210.p1	60S acidic ribosomal protein P3-2	2	71	12,022	protein	0.42	0.48	0.48
38	Morus007901.p1	Actin-7	30	717	41,897	cell	1.02	1.20	0.47
39	Morus007352.p1	Stem-specific protein TSJT1	7	96	25,521	metal handling	0.18	0.45	0.45
40	Morus026327.p1	Heat shock cognate 70 kDa protein 1	41	791	71,553	stress	0.91	0.67	0.44
41	Morus021433.p1	Malate dehydrogenase, cytoplasmic	20	463	35,912	TCA	0.57	0.95	0.43
42	Morus006184.p1	Cysteine synthase	33	572	34,400	amino acid metabolism	1.77	0.59	0.39
43	Morus018842.p1	2-Cys peroxiredoxin BAS1-like, chloroplastic	23	321	29,121	redox	0.81	0.65	0.39
44	Morus022454.p1	Fasciclin-like arabinogalactan protein 8	6	120	43,455	cell wall	0.22	0.16	0.39
45	Morus008883.p1	Uncharacterized protein	6	104	49,487	signaling	0.17	0.34	0.38
46	Morus000210.p1	Calvin cycle protein CP12	8	288	14,542	photosynthesis	0.34	0.38	0.38
47	Morus002920.p1	Thioredoxin M-type 4, chloroplastic	6	164	20,233	redox	0.42	0.26	0.37
48	Morus018564.p1	Isoflavone reductase homolog P3	12	102	45,171	secondary metabolism	0.33	1.17	0.37
49	Morus013051.p1	Adenosine kinase 2	7	216	37,797	nucleotide metabolism	0.65	0.41	0.35
50	Morus000836.p1	Ribulose bisphosphate carboxylase large chain (Fragment)	290	4805	61,599	photosynthesis	1.18	0.63	0.33
51	Morus015202.p1	Uncharacterized protein	11	234	33,994	not assigned	0.46	0.39	0.32
52	Morus014140.p1	Plastid-lipid-associated protein, chloroplastic	10	281	35,137	cell	0.52	0.31	0.31
53	Morus025784.p1	Phospholipase D alpha 1	4	47	92,059	lipid metabolism	0.05	0.37	0.30
54	Morus018550.p1	Glycine-rich RNA-binding protein GRP1A	5	165	18,416	RNA	0.39	0.29	0.29
55	Morus014011.p1	Glycerophosphoryl diester phosphodiesterase 2	6	295	81,816	lipid metabolism	0.16	0.12	0.29
56	Morus019087.p1	Putative mitochondrial 2-oxoglutarate/malate carrier protein	12	194	32,224	transport	0.98	0.29	0.28
57	Morus002874.p1	Leucine aminopeptidase 3, chloroplastic	39	981	60,563	protein	0.96	1.09	0.27
58	Morus010230.p1	Superoxide dismutase [Cu-Zn]	2	63	20,420	redox	0.34	0.37	0.26
59	Morus019413.p1	Cysteine proteinase 15A	9	259	41,574	protein	0.23	0.12	0.26
60	Morus015818.p1	Probable glucan endo-1,3-beta-glucosidase A6	3	111	52,145	misc	0.15	0.17	0.24
61	Morus020384.p1	Cysteine synthase, chloroplastic/chromoplastic	21	258	43,997	amino acid metabolism	0.52	0.11	0.24
62	Morus013361.p1	Protein disulfide-isomerase	15	432	56,492	redox	0.66	0.92	0.22
63	Morus007114.p1	Glycine-rich RNA-binding protein 2	3	170	27,802	RNA	0.17	0.19	0.19
64	Morus011198.p1	L-ascorbate peroxidase, cytosolic	26	392	27,414	redox	1.05	1.13	0.19
65	Morus008123.p1	IAA-amino acid hydrolase ILR1-like 5	5	89	47,707	hormone metabolism	0.15	0.50	0.18
66	Morus024851.p1	Catalase isozyme 1	19	137	57,208	redox	0.62	0.32	0.18
67	Morus017351.p1	Serine carboxypeptidase-like 50	5	55	49,604	protein	0.15	0.14	0.17
68	Morus014667.p1	Alpha-xylosidase	15	257	103,539	misc	0.17	0.45	0.15
69	Morus017174.p1	Predicted protein	8	193	33,060	signaling	0.20	0.27	0.15
70	Morus024951.p1	Triosephosphate isomerase, chloroplastic	42	655	34,813	photosynthesis	1.66	0.99	0.15
71	Morus008661.p1	14-3-3-like protein A	15	283	81,889	cell	0.26	0.37	0.14
72	Morus016271.p1	Elongation factor 2	14	106	99,403	protein	0.11	0.44	0.14
73	Morus001657.p1	6-phosphogluconolactonase 4, chloroplastic	6	108	35,151	OPP	0.18	0.97	0.14
74	Morus009365.p1	5-methyltetrahydropteroyltriglutamate--homocysteine methyltransferase	13	188	84,904	amino acid metabolism	0.17	0.46	0.14
75	Morus007784.p1	UTP--glucose-1-phosphate uridylyltransferase	26	372	76,133	glycolysis	0.48	0.80	0.13
76	Morus017695.p1	31 kDa ribonucleoprotein, chloroplastic	10	230	38,128	RNA	0.17	0.13	0.13
77	Morus007494.p1	RuBisCO large subunit-binding protein subunit alpha, chloroplastic	30	964	62,000	photosynthesis	1.29	0.33	0.11
78	Morus011664.p1	L-ascorbate oxidase homolog	4	53	60,522	not assigned	0.13	0.37	0.11
79	Morus006060.p1	V-type proton ATPase subunit B2	10	400	63,333	transport	0.41	0.16	0.11
80	Morus004111.p1	Calreticulin	7	266	50,196	signaling	0.32	0.56	0.10
81	Morus013778.p1	Monodehydroascorbate reductase	13	142	49,982	redox	0.30	0.33	0.10
82	Morus024141.p1	Beta-D-xylosidase 4	6	187	84,604	cell wall	0.14	0.10	0.10
83	Morus007961.p1	Hypothetical protein	7	62	95,561	not assigned	0.05	0.07	0.09
84	Morus025925.p1	Alpha-glucosidase	6	207	93,365	misc	0.12	0.25	0.07

^a^ Protein ID, according to the *Morus* database; ^b^ M.P., number of matched peptides; ^c^ Function, function categorized using MapMan bin codes; ^d^ Mol (%), protein abundance; misc, miscellaneous; protein, protein synthesis/degradation/folding/targeting; cell, cell organization/vesicle transport; RNA, RNA processing/regulation of transcription; redox, redox ascorbate/glutathione metabolism; TCA, tricarboxylic acid cycle; and OPP, oxidative pentose phosphate.

## Supplementary Materials

Supplementary Materials can be found at http://www.mdpi.com/1422-0067/20/2/365/s1. Supplemental Figure S1. Comparison of organ-specific proteins related to secondary metabolism. Supplemental Figure S2. Experimental design of the proteomic study. Supplemental Figure S3. Workflow of the gel-free/label-free proteomic methods in the present study. Supplemental Figure S4. Pie chart of functional categorization of leaf, branch, and root in Morus. Supplemental Table S1. Proteins Identified in the leaf of *Morus* by gel-free/label-free proteomic analysis. Supplemental Table S2. Proteins Identified in the branch of *Morus* by gel-free/label-free proteomic analysis. Supplemental Table S3. Proteins Identified in the root of *Morus* by gel-free/label-free proteomic analysis. Supplemental Table S4. Contents of total flavonoids in different organs of *Morus*. Supplemental Table S5. Primers of three genes used in this study.

## Abbreviations

ABTS	2,2’-Azino-bis (3-ethylbenzothiazoline-6-sulfonic acid)
CHAPS	3-[(3-Cholamidopropyl) dimethylammonio] propanesulfonate
CHI	Chalcone flavanone isomerase
emPAI	Exponentially-modified protein abundance index
HPLC	High-performance liquid chromatography
ISO	Isoflavone reductase
IFRh	Isoflavonoid reductase homolog
MS	Mass spectrometry
MVA	Mevalonate
PGK	Phosphoglycerate kinase
POD	Peroxidase
qRT-PCR	Quantitative reverse transcription-polymerase chain reaction
SOD	Superoxide dismutase
TCA	Tricarboxylic acid
TEAC	Trolox-equivalent antioxidant capacity
