# Supplementary material for: Organ-Specific Analysis of Morus alba Using a Gel-Free/Label-Free Proteomic Technique
Source: Int J Mol Sci. 2019 Jan 16;20(2):365. doi: 10.3390/ijms20020365 (PMC6359061; doi:10.3390/ijms20020365)
Supplement: Supplementary file 1 [file ijms-20-00365-s001.pdf]

Supplementary Material

Organ-Specific Analysis of *Morus alba* Using a Gel-Free/Label-Free Proteomic Technique

Wei Zhu [1,†], Zhuoheng Zhong [1,†], Shengzhi Liu [1], Bingxian Yang [1], Setsuko Komatsu [2], Zhiwei Ge [3] and Jingkui Tian [1,4,*]

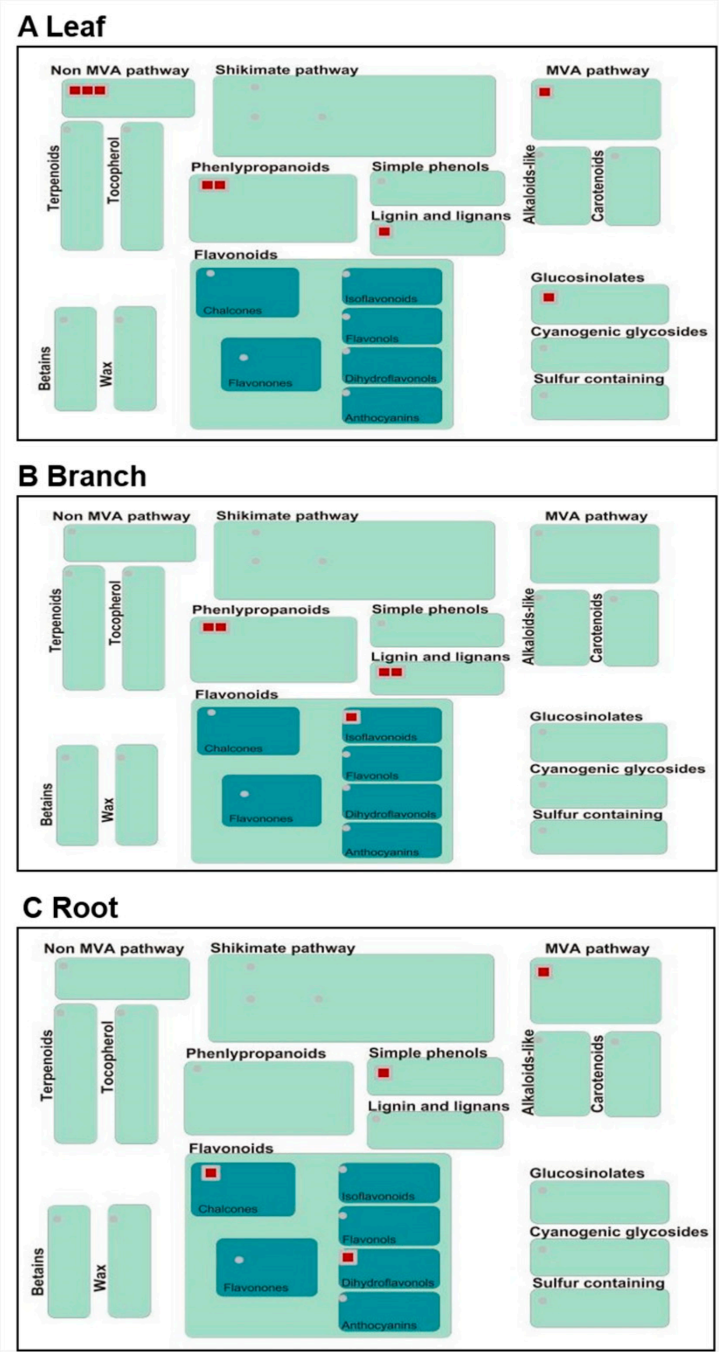

Supplemental Figure S1. Comparison of organ-specific proteins related to secondary metabolism. Leaf- (A), branch- (B), and root-specific (C) proteins related to secondary metabolism were submitted to the MapMan software (version 3.6.0RC1, Aachen, Germany). Each red square indicates one mapped protein.

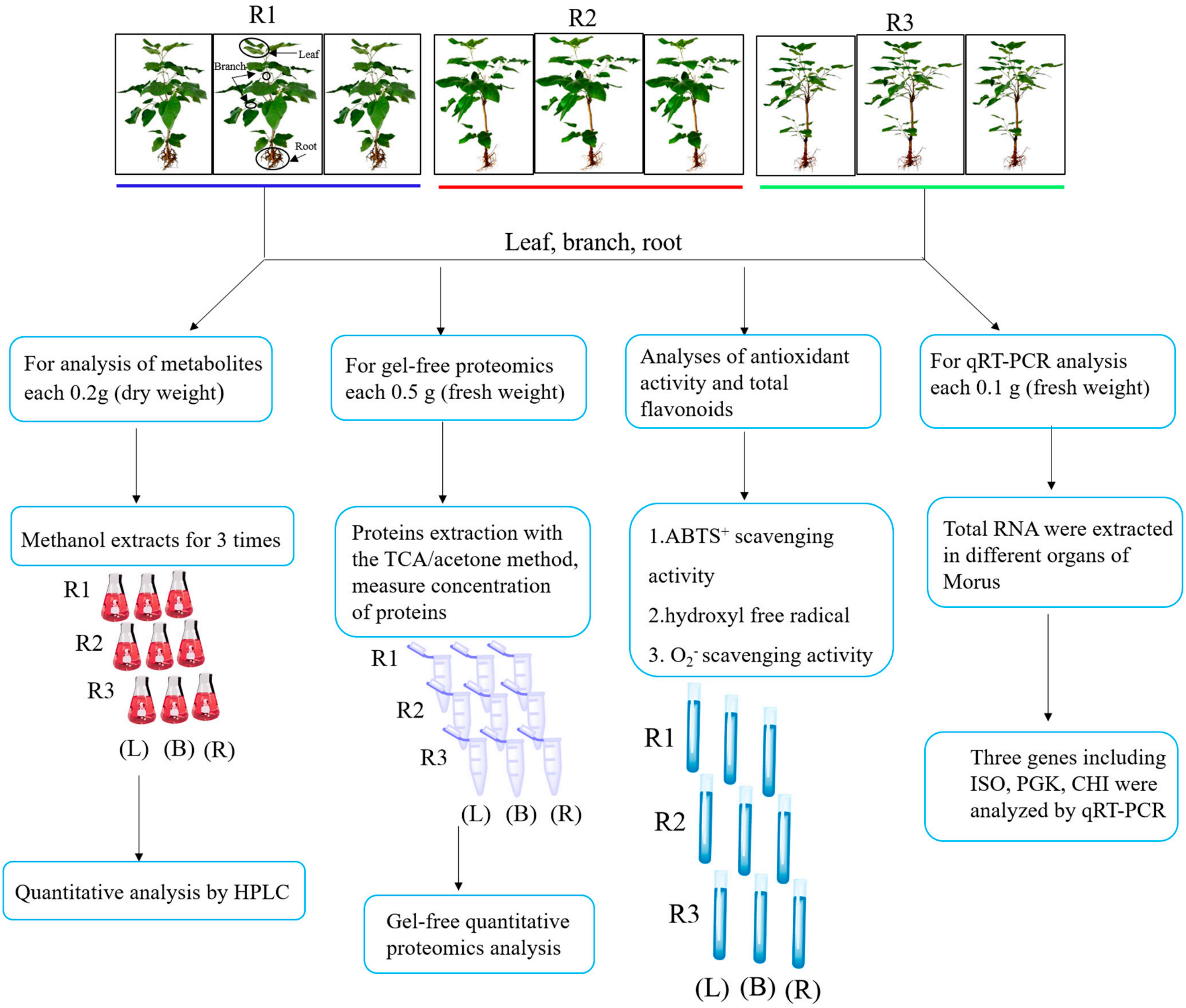

Supplemental Figure S2. Experimental design of the proteomic study. Leaves, branches, and roots were collected from three *Morus* individuals as one biological replicate. Three independent experiments were performed.

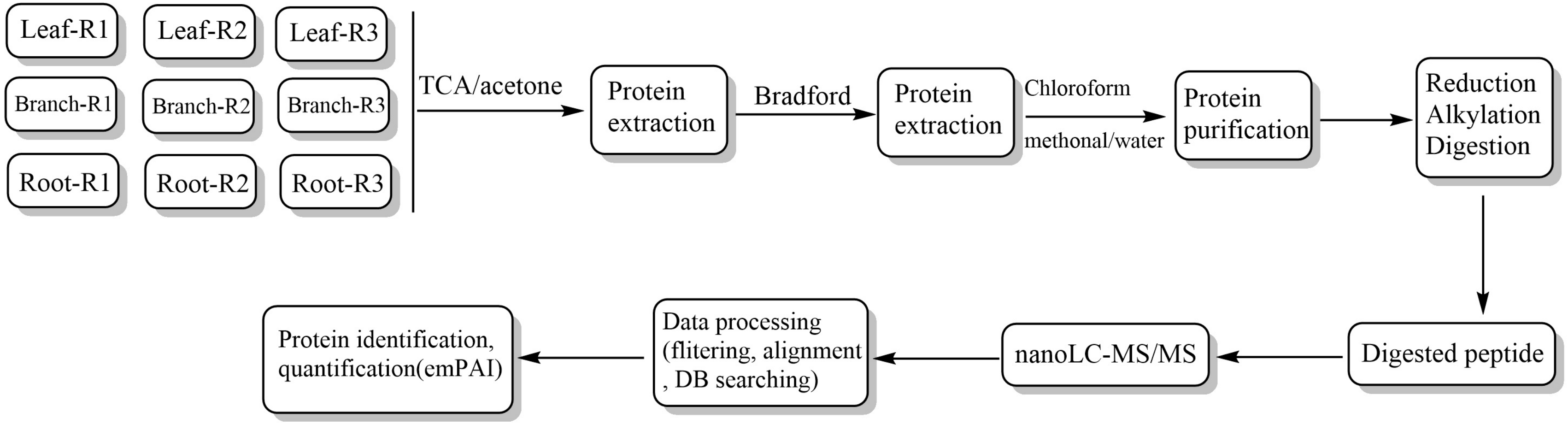

Supplemental Figure S3. Workflow of the gel-free/label-free proteomic methods in the present study.

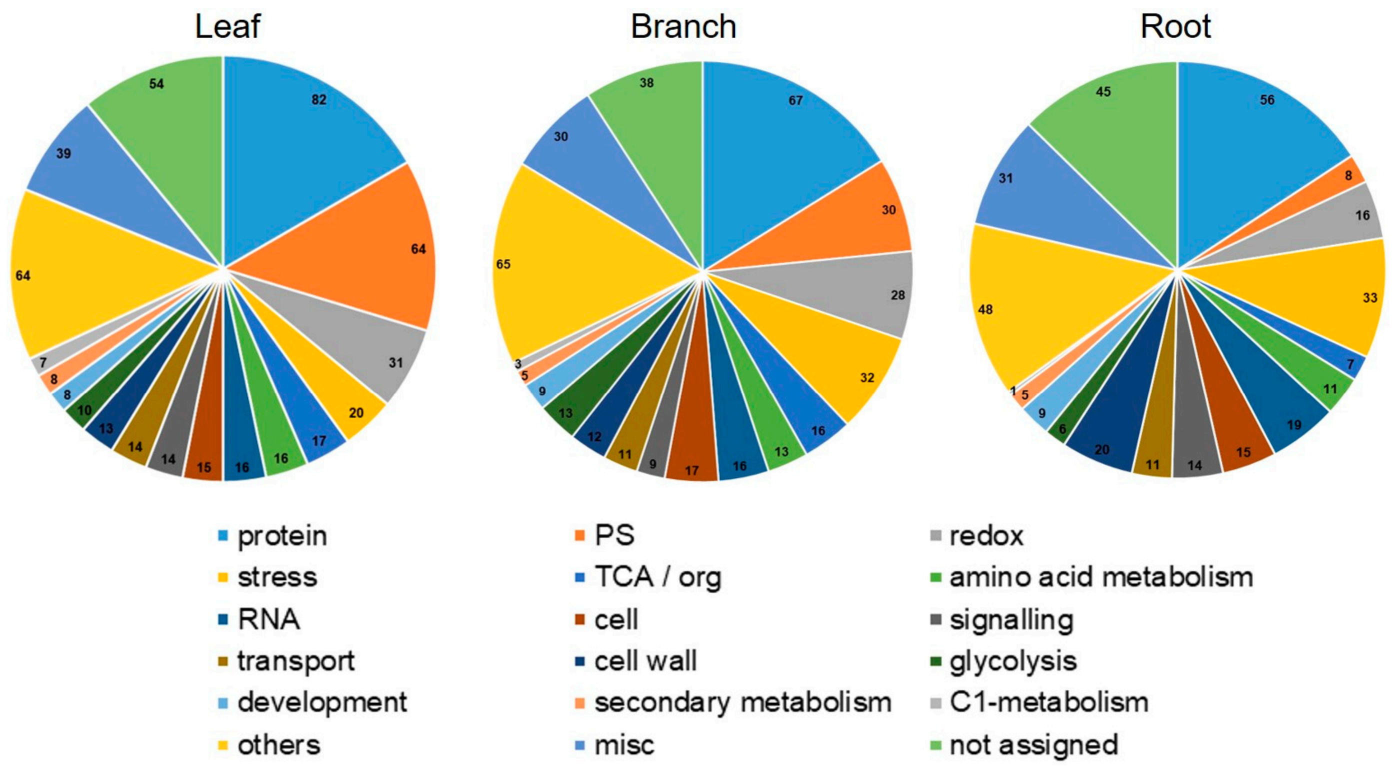

Supplemental Figure S4. Pie chart of functional categorization of leaf, branch, and root in *Morus*.

Supplemental Table S1. Proteins Identified in the Leaf of *Morus* by Gel-free/Label-free Proteomic Analysis

No	Protein ID [a]	Description	M.P. [b]	Score	Mol (%) [c]	Mass (Da)	Function [d]
1	Morus009492.p1	Macrophage migration inhibitory factor homolog	8	203	3.10	8961	not assigned
2	Morus013312.p1	Oxygen-evolving enhancer protein 2, chloroplastic	69	809	2.83	28487	photosynthesis
3	Morus025354.p1	Ribulose bisphosphate carboxylase/oxygenase activase,	122	3985	2.76	48142	photosynthesis
4	Morus003011.p1	Phosphoglycerate kinase, chloroplastic	77	1532	2.13	49827	photosynthesis
5	Morus018316.p1	Superoxide dismutase 1 copper chaperone	9	339	2.11	11171	metal handling
6	Morus001784.p1	ATP synthase epsilon chain, chloroplastic	15	363	2.03	14790	photosynthesis
7	Morus011742.p1	Oxygen-evolving enhancer protein 1, chloroplastic	111	2890	2.03	35257	photosynthesis
8	Morus003629.p1	Apocytochrome f	25	281	1.93	28559	photosynthesis
9	Morus025426.p1	Glutathione S-transferase DHAR1, mitochondrial	21	681	1.85	26882	redox
10	Morus006184.p1	Cysteine synthase	33	572	1.77	34400	amino acid metabolism
11	Morus003013.p1	Phosphoglycerate kinase, cytosolic	59	1143	1.72	42729	glycolysis
12	Morus014190.p1	Aminomethyltransferase, mitochondrial	46	886	1.67	44375	amino acid metabolism
13	Morus022811.p1	Phosphoribulokinase, chloroplastic	42	746	1.66	46380	photosynthesis
14	Morus024951.p1	Triosephosphate isomerase, chloroplastic	42	655	1.66	34813	photosynthesis
15	Morus014647.p1	Ribulose bisphosphate carboxylase small chain, chloroplastic	122	1239	1.63	20671	photosynthesis
16	Morus009000.p1	60S acidic ribosomal protein P2B	12	110	1.62	11673	protein
17	Morus024765.p1	Photosystem II CP43 chlorophyll apoprotein	11	207	1.47	18044	photosynthesis
18	Morus011314.p1	Glutamine synthetase leaf isozyme, chloroplastic	65	942	1.44	48057	N-metabolism
19	Morus014845.p1	Oxygen-evolving enhancer protein 3-2, chloroplastic	30	563	1.39	24703	photosynthesis
20	Morus001634.p1	Nucleoside diphosphate kinase 1	11	215	1.35	16322	nucleotide metabolism
21	Morus018988.p1	Elongation factor Tu, chloroplastic	55	958	1.30	52230	protein
22	Morus007494.p1	RuBisCO large subunit-binding protein subunit alpha,	30	964	1.29	62000	photosynthesis
23	Morus007512.p1	Kiwellin	20	762	1.29	24050	not assigned
24	Morus010743.p1	Triosephosphate isomerase, cytosolic	19	263	1.27	27548	glycolysis
25	Morus001112.p1	Malate dehydrogenase, glyoxysomal	29	798	1.26	37408	gluconeogenesis
26	Morus003661.p1	Lactoylglutathione lyase	19	313	1.25	38679	biodegradation of xenobiotics
27	Morus027698.p1	Alanine aminotransferase 2	34	963	1.25	53868	amino acid metabolism
28	Morus026672.p1	Ferredoxin--NADP reductase, leaf isozyme, chloroplastic	54	946	1.22	40191	photosynthesis
29	Morus001936.p1	Peroxiredoxin-2B	17	350	1.22	17391	redox
30	Morus001377.p1	Stromal 70 kDa heat shock-related protein, chloroplastic	47	1282	1.22	75481	stress

31	Morus018688.p1	Malate dehydrogenase, mitochondrial	37	1149	1.21	36695	TCA
32	Morus018888.p1	NAD dependent epimerase/dehydratase	41	476	1.18	42360	not assigned
33	Morus000836.p1	Ribulose bisphosphate carboxylase large chain (Fragment)	290	4805	1.18	61599	photosynthesis
34	Morus021638.p1	Probable peroxisomal (S)-2-hydroxy-acid oxidase 2	36	460	1.17	36982	photosynthesis
35	Morus014304.p1	Plastocyanin, chloroplastic	23	661	1.16	16620	photosynthesis
36	Morus027774.p1	Quinone oxidoreductase-like protein	21	592	1.16	40725	miscellaneousellaneous
37	Morus009593.p1	Auxin-binding protein ABP19a	154	623	1.16	22473	stress
38	Morus025917.p1	Photosystem I reaction center subunit III, chloroplastic	17	332	1.15	24882	photosynthesis
39	Morus006727.p1	Glyceraldehyde-3-phosphate dehydrogenase, cytosolic	30	711	1.12	37065	glycolysis
40	Morus014362.p1	Endochitinase 1	28	633	1.12	35841	stress
41	Morus023628.p1	Tubulin beta-1 chain	22	578	1.11	51015	cell
42	Morus017847.p1	Ribonuclease UK114	16	266	1.08	19960	RNA
43	Morus006427.p1	Thylakoid lumenal 16.5 kDa protein, chloroplastic	20	551	1.07	26247	not assigned
44	Morus007581.p1	Glyceraldehyde-3-phosphate dehydrogenase A, chloroplastic	25	677	1.06	43228	photosynthesis
45	Morus025862.p1	ATP synthase subunit beta, mitochondrial	33	1017	1.06	59400	mitochondrial electron transport
46	Morus026150.p1	Dihydrolipoyl dehydrogenase 1, mitochondrial	30	590	1.06	53054	TCA
47	Morus011198.p1	L-ascorbate peroxidase, cytosolic	26	392	1.05	27414	redox
48	Morus015139.p1	Regulator of ribonuclease-like protein 2	5	127	1.04	18062	C1-metabolism
49	Morus004086.p1	20 kDa chaperonin, chloroplastic	19	365	1.04	26730	protein
50	Morus022525.p1	Calmodulin	9	250	1.03	16894	signalling
51	Morus025068.p1	Uncharacterized protein	29	534	1.03	44547	RNA
52	Morus022592.p1	Thaumatin-like protein 1a	18	362	1.02	26984	stress
53	Morus011714.p1	Probable plastid-lipid-associated protein 6, chloroplastic	21	585	1.02	30747	cell
54	Morus025582.p1	Transketolase, chloroplastic	65	1241	1.02	80655	OPP
55	Morus007901.p1	Actin-7	30	717	1.02	41897	cell
56	Morus024957.p1	Peptidylprolyl isomerase	9	267	1.02	18291	cell
57	Morus020362.p1	Probable NADP-dependent oxidoreductase P2	22	446	1.02	38288	miscellaneousellaneous
58	Morus002489.p1	Nascent polypeptide-associated complex subunit alpha-like protein 1	11	285	1.01	22279	protein
59	Morus022215.p1	Cytochrome b6-f complex iron-sulfur subunit, chloroplastic	37	647	1.01	24759	photosynthesis
60	Morus020519.p1	50S ribosomal protein L12, chloroplastic	22	377	1.00	19940	protein
61	Morus019087.p1	Putative mitochondrial 2-oxoglutarate/malate carrier protein	12	194	0.98	32224	transport
62	Morus017723.p1	Chlorophyll a-b binding protein 8, chloroplastic	21	375	0.97	29521	photosynthesis

63	Morus002874.p1	Leucine aminopeptidase 3, chloroplastic	39	981	0.96	60563	protein
64	Morus018939.p1	Peptide methionine sulfoxide reductase (Fragment)	10	258	0.96	21860	protein
65	Morus000148.p1	S-formylglutathione hydrolase	3	75	0.95	18681	C1-metabolism
66	Morus002782.p1	Predicted protein	4	189	0.93	12851	not assigned
67	Morus014360.p1	Class I chitinase, putative	22	610	0.93	35679	stress
68	Morus013807.p1	Fructose-bisphosphate aldolase, cytoplasmic isozyme	23	584	0.92	38459	glycolysis
69	Morus026327.p1	Heat shock cognate 70 kDa protein 1	41	791	0.91	71553	stress
70	Morus003301.p1	40S ribosomal protein S12	10	195	0.89	13290	protein
71	Morus026318.p1	Heat shock cognate 70 kDa protein 2	38	651	0.89	71352	stress
72	Morus025300.p1	Uncharacterized protein	19	822	0.87	51118	not assigned
73	Morus002945.p1	RuBisCO large subunit-binding protein subunit beta,	35	1059	0.87	83597	protein
74	Morus018893.p1	PsbP domain-containing protein 1, chloroplastic	7	266	0.87	12182	photosynthesis
75	Morus008377.p1	Glycerate dehydrogenase	16	483	0.87	40570	amino acid metabolism
76	Morus028068.p1	Polygalacturonase inhibitor 1	10	306	0.86	37677	cell wall
77	Morus012656.p1	Chlorophyll a-b binding protein CP26, chloroplastic	21	409	0.86	39425	photosynthesis
78	Morus002855.p1	Chlorophyll a-b binding protein 40, chloroplastic	41	828	0.85	28113	photosynthesis
79	Morus020914.p1	Thylakoid lumenal 17.4 kDa protein, chloroplastic	10	351	0.85	26383	not assigned
80	Morus013363.p1	L-ascorbate peroxidase T, chloroplastic	20	453	0.84	45708	redox
81	Morus016574.p1	Glucan endo-1,3-beta-glucosidase, basic isoform	16	306	0.84	38364	miscellaneousellaneous
82	Morus021363.p1	Cell division protease ftsH homolog 2, chloroplastic	33	641	0.83	74368	protein
83	Morus017803.p1	Apolipoprotein D	15	321	0.82	38206	stress
84	Morus019127.p1	Protein binding protein	21	434	0.82	39137	not assigned
85	Morus018842.p1	2-Cys peroxiredoxin BAS1-like, chloroplastic	23	321	0.81	29121	redox
86	Morus017475.p1	Peptidyl-prolyl cis-trans isomerase CYP20-2, chloroplastic	23	492	0.81	28281	cell
87	Morus009083.p1	Methylenetetrahydrofolate dehydrogenase	7	220	0.78	28243	C1-metabolism
88	Morus025221.p1	Actin-1	25	598	0.78	41856	cell
89	Morus019010.p1	Thylakoid lumenal protein	11	442	0.78	36137	not assigned
90	Morus016237.p1	Sedoheptulose-1,7-bisphosphatase, chloroplastic	30	401	0.78	42916	photosynthesis
91	Morus007342.p1	Peroxiredoxin-2F, mitochondrial	12	178	0.77	22580	redox
92	Morus002102.p1	Serine--glyoxylate aminotransferase	16	451	0.76	39846	amino acid metabolism
93	Morus020532.p1	Glutaredoxin	5	244	0.75	15307	redox
94	Morus010676.p1	Superoxide dismutase [Mn], mitochondrial	21	247	0.74	26407	redox

95	Morus015920.p1	Photosystem I reaction center subunit IV B, chloroplastic	17	237	0.74	15399	photosynthesis
96	Morus011779.p1	Superoxide dismutase [Cu-Zn], chloroplastic	22	579	0.74	29603	redox
97	Morus006935.p1	Ferritin-3, chloroplastic	11	135	0.74	29626	metal handling
98	Morus012368.p1	Photosystem I reaction center subunit II, chloroplastic	31	511	0.73	23554	photosynthesis
99	Morus005391.p1	Thioredoxin M-type, chloroplastic	11	136	0.73	20426	redox
100	Morus020134.p1	FKBP-type peptidyl-prolyl cis-trans isomerase 4,	9	340	0.72	23758	protein
101	Morus022547.p1	Zeaxanthin epoxidase, chloroplastic	5	155	0.71	14278	not assigned
102	Morus007962.p1	Serine-threonine protein kinase	12	338	0.71	40358	not assigned
103	Morus025981.p1	Tubulin beta-3 chain	17	362	0.71	50624	cell
104	Morus016205.p1	Agglutinin alpha chain	20	375	0.70	15947	miscellaneousellaneous
105	Morus020497.p1	Quinone oxidoreductase	10	291	0.70	34081	miscellaneousellaneous
106	Morus001356.p1	Unknown	9	125	0.70	31673	not assigned
107	Morus000610.p1	Lipoxygenase 1	4	155	0.69	7887	hormone metabolism
108	Morus003374.p1	Uncharacterized protein	11	241	0.69	26692	not assigned
109	Morus018536.p1	Fructose-bisphosphate aldolase 1, chloroplastic	21	344	0.69	42531	photosynthesis
110	Morus019878.p1	Proteasome subunit beta type-6	7	245	0.69	26634	protein
111	Morus013522.p1	ATP synthase gamma chain, chloroplastic	12	292	0.69	41514	photosynthesis
112	Morus022430.p1	Proteasome subunit beta type-1	11	313	0.68	24861	protein
113	Morus020220.p1	Fructose-bisphosphate aldolase, cytoplasmic isozyme 1	13	548	0.68	38357	photosynthesis
114	Morus025517.p1	Tubulin alpha chain	18	426	0.68	49920	cell
115	Morus001516.p1	Mitochondrial outer membrane protein porin of 34 kDa	14	262	0.68	29598	transport
116	Morus011207.p1	Putative selenium-binding protein	25	546	0.67	55119	Metal handling
117	Morus018094.p1	Photosystem II stability/assembly factor HCF136, chloroplastic	12	316	0.67	45453	protein
118	Morus013122.p1	ATP synthase subunit b, chloroplastic	9	92	0.67	20103	photosynthesis
119	Morus003491.p1	Ferredoxin-thioredoxin reductase catalytic chain, chloroplastic	9	201	0.67	16643	redox
120	Morus007765.p1	Xyloglucan endotransglucosylase/hydrolase protein 6	19	304	0.67	33647	cell wall
121	Morus013361.p1	Protein disulfide-isomerase	15	432	0.66	56492	redox
122	Morus013051.p1	Adenosine kinase 2	7	216	0.65	37797	nucleotide metabolism
123	Morus015899.p1	Alcohol dehydrogenase class-3	23	481	0.65	43063	miscellaneousellaneous
124	Morus020016.p1	Chlorophyll a-b binding protein 151, chloroplastic	19	219	0.65	28504	photosynthesis
125	Morus017402.p1	Guanine nucleotide-binding protein subunit beta-like protein	15	370	0.64	36552	development
126	Morus016996.p1	Ribulose bisphosphate carboxylase/oxygenase activase 1, chloroplastic	60	1169	0.64	52694	photosynthesis

127	Morus025411.p1	Photosystem I P700 chlorophyll a apoprotein A2	4	55	0.64	8476	photosynthesis
128	Morus010361.p1	Flocculation protein FLO11	40	775	0.64	48806	lipid metabolism
129	Morus013212.p1	Eukaryotic translation initiation factor 5A-2	12	182	0.63	17687	protein
130	Morus004210.p1	Glucan endo-1,3-beta-glucosidase, basic vacuolar isoform	20	178	0.63	39002	miscellaneousellaneous
131	Morus024614.p1	Heme-binding protein 2	4	202	0.62	25414	tetrapyrrole synthesis
132	Morus024851.p1	Catalase isozyme 1	19	137	0.62	57208	redox
133	Morus003800.p1	V-type proton ATPase catalytic subunit A	28	457	0.61	68994	transport
134	Morus003300.p1	NADP-dependent glyceraldehyde-3-phosphate dehydrogenase	38	497	0.60	54041	glycolysis
135	Morus008669.p1	Allene oxide cyclase 2, chloroplastic	6	168	0.60	27569	hormone metabolism
136	Morus019506.p1	Glycine dehydrogenase [decarboxylating], mitochondrial	59	968	0.60	115851	C1-metabolism
137	Morus018505.p1	NADP-dependent oxidoreductase P2	9	296	0.59	40979	miscellaneousellaneous
138	Morus009923.p1	Photosystem I reaction center subunit psaK, chloroplastic	6	156	0.58	13118	photosynthesis
139	Morus003622.p1	Uncharacterized protein	6	158	0.58	24597	not assigned
140	Morus004201.p1	Universal stress protein A-like protein	9	223	0.58	18591	stress
141	Morus007339.p1	Glucose-1-phosphate adenylyltransferase large subunit 1, chloroplastic	23	460	0.58	58263	major CHO metabolism
142	Morus003829.p1	NAD(P)H-quinone oxidoreductase subunit M, chloroplastic	13	205	0.58	24846	photosynthesis
143	Morus023303.p1	Glycine cleavage system H protein 2, mitochondrial	16	534	0.57	18914	photosynthesis
144	Morus000248.p1	Lachrymatory-factor synthase	4	162	0.57	18846	not assigned
145	Morus021433.p1	Malate dehydrogenase, cytoplasmic	20	463	0.57	35912	TCA
146	Morus000029.p1	Photosystem Q(B) protein	10	194	0.57	34748	photosynthesis
147	Morus001961.p1	Peroxidase 12	16	244	0.57	38426	miscellaneousellaneous
148	Morus009076.p1	Glutathione S-transferase 6, chloroplastic	8	155	0.57	28645	miscellaneousellaneous
149	Morus026718.p1	Probable fructose-bisphosphate aldolase 2, chloroplastic	21	373	0.56	44113	photosynthesis
150	Morus026654.p1	L-ascorbate peroxidase 3, peroxisomal	12	358	0.56	36515	redox
151	Morus017852.p1	Thioredoxin	9	250	0.56	19335	redox
152	Morus021189.p1	Hypothetical protein	5	139	0.55	23480	not assigned
153	Morus024190.p1	Probable plastid-lipid-associated protein 4, chloroplastic	13	150	0.55	32048	cell
154	Morus021346.p1	Protein notum homolog	9	250	0.54	43696	cell wall
155	Morus016604.p1	(3R)-hydroxymyristoyl-[acyl-carrier-protein] dehydratase	4	106	0.54	23809	lipid metabolism
156	Morus019089.p1	Tubulin alpha-3/alpha-5 chain	16	250	0.54	50214	cell
157	Morus004407.p1	Unknown	17	407	0.54	32727	not assigned
158	Morus008689.p1	Unknown	2	171	0.54	9765	signalling

159	Morus015151.p1	Thioredoxin F-type, chloroplastic	4	119	0.53	20251	redox
160	Morus021269.p1	Uncharacterized protein	12	241	0.53	34892	not assigned
161	Morus020384.p1	Cysteine synthase, chloroplastic/chromoplastic	21	258	0.52	43997	amino acid metabolism
162	Morus014140.p1	Plastid-lipid-associated protein, chloroplastic	10	281	0.52	35137	cell
163	Morus017876.p1	Fructose-1,6-bisphosphatase, cytosolic	9	155	0.51	37605	major CHO metabolism
164	Morus015811.p1	Quinone-oxidoreductase homolog, chloroplastic	13	191	0.51	35313	miscellaneousellaneous
165	Morus015572.p1	Chlorophyll a-b binding protein CP29.1, chloroplastic	17	290	0.51	31544	photosynthesis
166	Morus004100.p1	Peroxiredoxin Q, chloroplastic	4	114	0.50	23684	redox
167	Morus008192.p1	Protein cbbY	13	341	0.50	36041	not assigned
168	Morus002098.p1	PsbP domain-containing protein 6, chloroplastic	7	159	0.50	29451	photosynthesis
169	Morus003952.p1	Lipoxygenase homology domain-containing protein 1	6	154	0.50	21171	not assigned
170	Morus016874.p1	Apolipoprotein d	4	72	0.50	21306	transport
171	Morus023908.p1	Uncharacterized protein	15	271	0.50	57888	protein
172	Morus015082.p1	Auxin-repressed 12.5 kDa protein	3	89	0.49	13355	development
173	Morus026213.p1	Photosystem I reaction center subunit VI, chloroplastic	14	143	0.49	15137	photosynthesis
174	Morus017899.p1	Hypothetical protein	5	97	0.49	23791	not assigned
175	Morus011997.p1	Probable pectinesterase/pectinesterase inhibitor 6	19	668	0.49	50473	cell wall
176	Morus026660.p1	Proteasome subunit alpha type-7	5	154	0.49	26129	protein
177	Morus007784.p1	UTP--glucose-1-phosphate uridylyltransferase	26	372	0.48	76133	glycolysis
178	Morus010420.p1	Soluble inorganic pyrophosphatase 1, chloroplastic	12	234	0.47	33355	nucleotide metabolism
179	Morus004838.p1	Uncharacterized protein	19	404	0.47	65234	signalling
180	Morus007482.p1	Fumarate hydratase 1, mitochondrial	13	363	0.46	53693	TCA
181	Morus015202.p1	Uncharacterized protein	11	234	0.46	33994	not assigned
182	Morus024109.p1	50S ribosomal protein L11, chloroplastic	10	151	0.46	25513	protein
183	Morus024292.p1	Glutathione peroxidase	6	116	0.46	18602	redox
184	Morus025405.p1	PsbP-like protein 1, chloroplastic	6	135	0.46	27439	photosynthesis
185	Morus022381.p1	Uncharacterized oxidoreductase	8	278	0.46	43697	minor CHO metabolism
186	Morus023512.p1	Translationally-controlled tumor protein homolog	12	127	0.45	18888	development
187	Morus000821.p1	Hexokinase-1	2	81	0.44	11518	major CHO metabolism
188	Morus026837.p1	Serine hydroxymethyltransferase 1	15	286	0.44	52275	C1-metabolism
189	Morus027772.p1	Carbonic anhydrase, chloroplastic	11	227	0.44	28452	TCA
190	Morus019389.p1	Ferredoxin-dependent glutamate synthase 1, chloroplastic	19	408	0.44	61859	N-metabolism

191	Morus020641.p1	Phosphoglycolate phosphatase	10	97	0.43	40477	photosynthesis
192	Morus001983.p1	Enolase	24	618	0.43	45388	glycolysis
193	Morus008166.p1	Proteasome subunit alpha type-6	8	207	0.43	33732	protein
194	Morus022003.p1	Pyridoxal biosynthesis protein PDX1	7	228	0.43	33507	Co-factor and vitamine metabolism
195	Morus020343.p1	40S ribosomal protein SA	5	78	0.42	32102	protein
196	Morus003616.p1	Fructokinase-2	10	137	0.42	35370	major CHO metabolism
197	Morus009210.p1	60S acidic ribosomal protein P3-2	2	71	0.42	12022	protein
198	Morus002920.p1	Thioredoxin M-type 4, chloroplastic	6	164	0.42	20233	redox
199	Morus004767.p1	Thioredoxin-X, chloroplastic	5	116	0.41	20474	redox
200	Morus020652.p1	UPF0603 protein	11	92	0.41	32117	not assigned
201	Morus006060.p1	V-type proton ATPase subunit B2	10	400	0.41	63333	transport
202	Morus007268.p1	Aspartate aminotransferase, chloroplastic	9	213	0.41	50775	amino acid metabolism
203	Morus024124.p1	Chlorophyll a-b binding protein	8	304	0.41	27437	photosynthesis
204	Morus002960.p1	Glutathione S-transferase	18	56	0.41	25231	miscellaneousellaneous
205	Morus020508.p1	Protease Do-like 1, chloroplastic	13	378	0.41	47860	protein
206	Morus007219.p1	Hypothetical protein	14	80	0.40	25566	not assigned
207	Morus017921.p1	3-ketoacyl-CoA thiolase 2, peroxisomal	9	138	0.39	49206	amino acid metabolism
208	Morus017990.p1	Pectinesterase 3	10	72	0.39	39257	cell wall
209	Morus027796.p1	Aldehyde dehydrogenase family 2 member B7	14	196	0.39	58405	fermentation
210	Morus018550.p1	Glycine-rich RNA-binding protein GRP1A	5	165	0.39	18416	RNA
211	Morus002978.p1	Unknown	4	98	0.38	23257	not assigned
212	Morus008699.p1	Phosphate carrier protein, mitochondrial	10	116	0.38	40133	transport
213	Morus001712.p1	PsbP domain-containing protein 4, chloroplastic	10	211	0.37	29642	photosynthesis
214	Morus009329.p1	Aminotransferase y4uB	11	191	0.37	51671	amino acid metabolism
215	Morus018282.p1	Cysteine-rich repeat secretory protein	6	175	0.37	27945	signalling
216	Morus011938.p1	Dihydrolipoyllysine-residue succinyltransferase	13	139	0.37	46499	TCA
217	Morus018049.p1	Putative lactoylglutathione lyase	11	207	0.37	33175	biodegradation of xenobiotics
218	Morus021898.p1	Thioredoxin H-type 1	2	122	0.37	19443	redox
219	Morus022569.p1	Conserved hypothetical protein	5	162	0.36	13788	not assigned
220	Morus017991.p1	Pectinesterase 1	5	77	0.36	25563	cell wall
221	Morus007366.p1	Alpha-1,4-glucan-protein synthase	8	187	0.36	41966	cell wall
222	Morus005554.p1	Chaperon P13.9	2	48	0.36	13862	development

223	Morus008884.p1	Cysteine proteinase RD21a	10	350	0.35	52217	protein
224	Morus016650.p1	Auxin-induced protein PCNT115	7	253	0.35	24910	hormone metabolism
225	Morus019388.p1	Ferredoxin-dependent glutamate synthase, chloroplastic	23	441	0.35	117231	N-metabolism
226	Morus005931.p1	Succinyl-CoA ligase [GDP-forming] subunit beta	13	144	0.35	45635	TCA
227	Morus007651.p1	60S ribosomal protein L12	4	48	0.35	17956	protein
228	Morus008428.p1	Formate dehydrogenase, mitochondrial	15	57	0.35	42456	C1-metabolism
229	Morus018739.p1	Chlorophyll a-b binding protein 13, chloroplastic	9	399	0.35	28571	photosynthesis
230	Morus008822.p1	Glutamate-1-semialdehyde 2,1-aminomutase 1, chloroplastic	11	362	0.35	51141	tetrapyrrole synthesis
231	Morus012127.p1	Polyphenol oxidase, chloroplastic	18	357	0.35	66197	protein
232	Morus010230.p1	Superoxide dismutase [Cu-Zn]	2	63	0.34	20420	redox
233	Morus015887.p1	Photosystem II reaction center photosynthesisB28 protein, chloroplastic	4	129	0.34	20419	photosynthesis
234	Morus006708.p1	2-C-methyl-D-erythritol 2,4-cyclodiphosphate synthase, chloroplastic	6	49	0.34	25833	secondary metabolism
235	Morus004398.p1	Serine protease inhibitor 6	4	51	0.34	26963	stress
236	Morus021009.p1	Mitochondrial-processing peptidase subunit beta	17	166	0.34	59133	protein
237	Morus017397.p1	Beta-glucosidase 44	16	128	0.34	59118	miscellaneousellaneous
238	Morus017091.p1	Superoxide dismutase [Fe], chloroplastic	8	259	0.34	35185	redox
239	Morus024833.p1	Glyceraldehyde-3-phosphate dehydrogenase B, chloroplastic	71	1889	0.34	148833	photosynthesis
240	Morus000210.p1	Calvin cycle protein CP12	8	288	0.34	14542	photosynthesis
241	Morus011027.p1	Ferredoxin-thioredoxin reductase	8	52	0.34	18669	redox
242	Morus001656.p1	Ubiquitin	2	65	0.33	14892	protein
243	Morus013420.p1	Chlorophyll a-b binding protein 6A, chloroplastic	7	154	0.33	26675	photosynthesis
244	Morus015956.p1	Ribulose-phosphate 3-epimerase, chloroplastic	6	185	0.33	30347	photosynthesis
245	Morus018564.p1	Isoflavone reductase homolog P3	12	102	0.33	45171	secondary metabolism
246	Morus018115.p1	FKBP-type peptidyl-prolyl cis-trans isomerase 2, chloroplastic	6	41	0.33	24976	protein
247	Morus022986.p1	Cathepsin B	6	173	0.33	38578	protein
248	Morus015275.p1	ADP, ATP carrier protein, mitochondrial (Fragment)	9	136	0.32	42142	transport
249	Morus004111.p1	Calreticulin	7	266	0.32	50196	signalling
250	Morus009012.p1	Proteasome subunit alpha type-1-B	4	52	0.32	31190	protein
251	Morus026500.p1	Endoplasmin homolog	63	369	0.32	92356	stress
252	Morus008423.p1	Peptidyl-prolyl cis-trans isomerase, chloroplastic	15	418	0.32	52692	protein
253	Morus011230.p1	Alpha-galactosidase	8	92	0.32	46358	minor CHO metabolism
254	Morus024220.p1	Monodehydroascorbate reductase, chloroplastic	16	313	0.32	53498	redox

255	Morus027609.p1	Unknown	7	103	0.32	25788	protein
256	Morus002067.p1	Glycine-rich RNA-binding protein 2, mitochondrial	2	106	0.31	15592	RNA
257	Morus010535.p1	Prohibitin-2	10	151	0.31	31627	mitochondrial electron transport
258	Morus014376.p1	Uncharacterized protein	7	104	0.31	44842	not assigned
259	Morus026246.p1	Hypothetical protein	3	66	0.31	22410	photosynthesis
260	Morus010076.p1	Glutathione reductase, cytosolic	16	284	0.31	54912	redox
261	Morus007916.p1	Aldehyde dehydrogenase	13	257	0.31	64984	fermentation
262	Morus018127.p1	Membrane-associated 30 kDa protein, chloroplastic	6	117	0.30	35490	RNA
263	Morus021008.p1	40S ribosomal protein S19-3	6	53	0.30	15938	protein
264	Morus004463.p1	Ferredoxin	2	55	0.30	16040	miscellaneousellaneous
265	Morus013778.p1	Monodehydroascorbate reductase	13	142	0.30	49982	redox
266	Morus006467.p1	Intracellular protease 1	9	219	0.30	42514	not assigned
267	Morus004803.p1	Ketol-acid reductoisomerase, chloroplastic	12	343	0.30	46064	amino acid metabolism
268	Morus013867.p1	S-adenosylmethionine synthetase 2	8	237	0.30	43654	amino acid metabolism
269	Morus026826.p1	Succinate dehydrogenase [ubiquinone] flavoprotein subunit 1	15	362	0.30	69366	TCA
270	Morus026982.p1	Allene oxide synthase, chloroplastic	11	164	0.30	56861	hormone metabolism
271	Morus017143.p1	Conserved hypothetical protein	2	58	0.29	16590	not assigned
272	Morus004387.p1	Photosystem II 22 kDa protein, chloroplastic	4	154	0.29	29604	photosynthesis
273	Morus013680.p1	Proteasome subunit alpha type-3	7	145	0.29	27514	protein
274	Morus022764.p1	2,3-bisphosphoglycerate-independent phosphoglycerate mutase	20	254	0.29	61217	glycolysis
275	Morus017078.p1	Peptide methionine sulfoxide reductase	4	131	0.29	29553	protein
276	Morus012569.p1	Glutathione reductase, chloroplastic (Fragment)	14	196	0.29	62981	redox
277	Morus023257.p1	Mitochondrial carnitine/acylcarnitine carrier-like protein	10	75	0.29	40163	transport
278	Morus020098.p1	Small heat shock protein C2	6	176	0.29	34449	stress
279	Morus014310.p1	Thioredoxin H-type 4	4	110	0.29	34297	redox
280	Morus004336.p1	WW domain-containing oxidoreductase	9	187	0.29	29839	miscellaneousellaneous
281	Morus015849.p1	Uncharacterized protein	7	179	0.29	16911	not assigned
282	Morus023635.p1	Fructose-1,6-bisphosphatase, chloroplastic	11	230	0.28	45296	photosynthesis
283	Morus000834.p1	ATP synthase subunit beta, chloroplastic	11	144	0.28	17084	photosynthesis
284	Morus005114.p1	Nuclear protein NP60	11	152	0.28	39477	OPP
285	Morus007626.p1	Ubiquitin-conjugating enzyme E2 2	2	55	0.28	17452	protein
286	Morus005387.p1	Thiol:disulfide interchange protein txlA homolog	5	171	0.28	28943	protein

287	Morus005460.p1	Proteasome subunit beta type-5	3	158	0.27	29253	protein
288	Morus022202.p1	Protein THYLAKOID FORMATION1, chloroplastic	5	67	0.27	34026	protein
289	Morus012437.p1	40S ribosomal protein S5 (Fragment)	7	56	0.27	22844	protein
290	Morus016969.p1	Uracil phosphoribosyltransferase	9	76	0.26	32116	nucleotide metabolism
291	Morus005351.p1	L-idonate 5-dehydrogenase	6	60	0.26	40268	minor CHO metabolism
292	Morus017908.p1	Voltage-gated potassium channel subunit beta	7	44	0.26	37314	transport
293	Morus005497.p1	Elongation factor 1-delta 1	10	130	0.26	26235	protein
294	Morus025788.p1	Conserved hypothetical protein	2	45	0.26	18479	development
295	Morus021726.p1	Carboxyvinyl-carboxyphosphonate phosphorylmutase	15	282	0.26	56407	not assigned
296	Morus025863.p1	Fruit protein pKIWI502	7	48	0.26	30781	photosynthesis
297	Morus017382.p1	Calcium-binding protein CML27	4	116	0.26	18705	signalling
298	Morus006034.p1	18.5 kDa class I heat shock protein	2	146	0.26	18598	stress
299	Morus001363.p1	Chlorophyll a-b binding protein, chloroplastic	3	59	0.26	31112	photosynthesis
300	Morus008661.p1	14-3-3-like protein A	15	283	0.26	81889	cell
301	Morus020701.p1	Photosystem I reaction center subunit N, chloroplastic	7	99	0.26	18618	photosynthesis
302	Morus000146.p1	Thylakoid lumenal 19 kDa protein, chloroplastic	11	85	0.25	26494	not assigned
303	Morus001781.p1	Photosystem II CP47 chlorophyll apoprotein	7	220	0.25	50773	photosynthesis
304	Morus011993.p1	Phosphoglucomutase, cytoplasmic	13	181	0.25	63757	glycolysis
305	Morus024890.p1	Cell division protease ftsH homolog, chloroplastic	21	457	0.25	76263	protein
306	Morus012310.p1	Uroporphyrinogen decarboxylase, chloroplastic	10	48	0.25	26608	tetrapyrrole synthesis
307	Morus008001.p1	Malate dehydrogenase, chloroplastic	8	216	0.25	49140	TCA
308	Morus010836.p1	40S ribosomal protein S15a-1	2	41	0.25	19069	protein
309	Morus027355.p1	40S ribosomal protein S3-3	5	79	0.25	35945	protein
310	Morus017748.p1	Plastid-lipid-associated protein 3, chloroplastic	8	97	0.24	40414	cell
311	Morus017207.p1	Proteasome subunit alpha type-4	6	173	0.24	27440	protein
312	Morus024255.p1	Aminopeptidase N	26	205	0.24	107865	protein
313	Morus014322.p1	Conserved hypothetical protein	4	73	0.24	35760	not assigned
314	Morus014494.p1	30S ribosomal protein S5, chloroplastic	5	238	0.23	36310	protein
315	Morus000857.p1	26S protease regulatory subunit 6A homolog	6	121	0.23	20506	protein
316	Morus004996.p1	Flavoprotein wrbA	5	103	0.23	20275	lipid metabolism
317	Morus019413.p1	Cysteine proteinase 15A	9	259	0.23	41574	protein
318	Morus023973.p1	Peptidyl-prolyl cis-trans isomerase H	3	69	0.23	20594	cell

319	Morus007159.p1	Uncharacterized protein	3	170	0.23	28739	not assigned
320	Morus012704.p1	Coproporphyrinogen-III oxidase, chloroplastic	7	94	0.23	44931	tetrapyrrole synthesis
321	Morus018600.p1	Hypothetical protein	4	76	0.23	26529	not assigned
322	Morus006591.p1	Isocitrate dehydrogenase	6	203	0.23	40226	TCA
323	Morus021090.p1	Mitochondrial outer membrane protein porin of 36 kDa	4	138	0.23	29418	transport
324	Morus008122.p1	Stem 28 kDa glycoprotein	2	126	0.23	29466	miscellaneousellaneous
325	Morus015157.p1	Chaperonin CPN60-2, mitochondrial	8	234	0.23	61639	protein
326	Morus010371.p1	Aspartate aminotransferase, mitochondrial	12	222	0.22	43136	amino acid metabolism
327	Morus022454.p1	Fasciclin-like arabinogalactan protein 8	6	120	0.22	43455	cell wall
328	Morus012965.p1	Predicted protein	2	33	0.22	21111	development
329	Morus013768.p1	Ankyrin repeat domain-containing protein 2	8	139	0.22	46613	RNA
330	Morus024265.p1	Aquaporin PIP1-3	5	67	0.22	30856	transport
331	Morus024212.p1	ATP-dependent Clp protease proteolytic subunit 6	7	95	0.21	36288	protein
332	Morus018783.p1	Nitrogen regulatory protein P-II	10	37	0.21	22307	signalling
333	Morus007731.p1	GTP-binding protein SAR1A	6	60	0.21	22126	signalling
334	Morus017087.p1	Glutamate dehydrogenase 2	9	204	0.21	44675	N-metabolism
335	Morus013364.p1	Aldehyde dehydrogenase family 3 member H1	7	42	0.21	54449	fermentation
336	Morus004812.p1	14-3-3-like protein D	6	172	0.21	73254	signalling
337	Morus024571.p1	Pro-hevein	2	91	0.21	23009	stress
338	Morus018244.p1	30S ribosomal protein S10, chloroplastic	3	24	0.21	28631	protein
339	Morus003021.p1	3-mercaptopyruvate sulfurtransferase	5	81	0.20	41188	amino acid metabolism
340	Morus015127.p1	Photosystem I reaction center subunit XI, chloroplastic	3	39	0.20	23180	photosynthesis
341	Morus001079.p1	Adenylate kinase 2, chloroplastic	4	69	0.20	32711	nucleotide metabolism
342	Morus026631.p1	Conserved hypothetical protein	4	145	0.20	23352	not assigned
343	Morus027852.p1	Pyruvate dehydrogenase E1 component subunit alpha	8	98	0.20	36217	TCA
344	Morus006152.p1	GDSL esterase/lipase	10	33	0.20	32970	miscellaneousellaneous
345	Morus017594.p1	Uncharacterized protein	10	360	0.20	96824	not assigned
346	Morus025974.p1	Reticuline oxidase-like protein	16	138	0.20	61753	miscellaneousellaneous
347	Morus006886.p1	Glucose-1-phosphate adenylyltransferase small subunit 2	12	129	0.20	57117	major CHO metabolism
348	Morus018656.p1	Epidermis-specific secreted glycoprotein EP1	10	245	0.20	48964	miscellaneousellaneous
349	Morus020347.p1	Formin-like protein 5	4	83	0.20	33422	not assigned
350	Morus024964.p1	Putative amidase C869.01	6	104	0.20	47961	miscellaneousellaneous

351	Morus017174.p1	Predicted protein	8	193	0.20	33060	signalling
352	Morus012896.p1	Pectinesterase/pectinesterase inhibitor	11	243	0.19	60870	cell wall
353	Morus016740.p1	NifU-like protein 3, chloroplastic	5	154	0.19	58350	protein
354	Morus017236.p1	50S ribosomal protein L10, chloroplastic	5	70	0.19	25325	protein
355	Morus014749.p1	Glutamate dehydrogenase 1	10	252	0.19	44436	N-metabolism
356	Morus001657.p1	6-phosphogluconolactonase 4, chloroplastic	6	108	0.18	35151	OPP
357	Morus005976.p1	30S ribosomal protein S1, chloroplastic	36	60	0.18	46489	protein
358	Morus026600.p1	UPF0548 protein	4	74	0.18	25718	not assigned
359	Morus004280.p1	Aspartic proteinase nepenthesin-1	3	98	0.18	53453	RNA
360	Morus027612.p1	Small heat shock protein, chloroplastic	3	42	0.18	25588	stress
361	Morus007352.p1	Stem-specific protein TSJT1	7	96	0.18	25521	metal handling
362	Morus005045.p1	GDSL esterase/lipase 1	4	98	0.18	42286	miscellaneousellaneous
363	Morus010536.p1	Fasciclin-like arabinogalactan protein 13	3	84	0.18	25698	cell wall
364	Morus001844.p1	THO complex subunit 4	4	164	0.18	26090	RNA
365	Morus005596.p1	beta-1,3-glucanase	11	99	0.18	36954	miscellaneousellaneous
366	Morus001782.p1	Cytochrome b6	2	180	0.18	26503	photosynthesis
367	Morus028058.p1	Polygalacturonase inhibitor	8	160	0.17	37452	cell wall
368	Morus021132.p1	DAG protein, chloroplastic	5	44	0.17	27490	development
369	Morus014281.p1	Hypothetical protein	2	145	0.17	27268	miscellaneousellaneous
370	Morus015140.p1	Isopentenyl-diphosphate Delta-isomerase II	8	78	0.17	27253	secondary metabolism
371	Morus010823.p1	Rubber elongation factor	3	46	0.17	27896	not assigned
372	Morus008883.p1	Uncharacterized protein	6	104	0.17	49487	signalling
373	Morus009365.p1	5-methyltetrahydropteroyltriglutamate--homocysteine methyltransferase	13	188	0.17	84904	amino acid metabolism
374	Morus018097.p1	ATP-dependent Clp protease proteolytic subunit 2	4	127	0.17	27076	protein
375	Morus002800.p1	Phosphoglucomutase, chloroplastic	9	237	0.17	65708	glycolysis
376	Morus007114.p1	Glycine-rich RNA-binding protein 2	3	170	0.17	27802	RNA
377	Morus014667.p1	Alpha-xylosidase	15	257	0.17	103539	miscellaneousellaneous
378	Morus017695.p1	31 kDa ribonucleoprotein, chloroplastic	10	230	0.17	38128	RNA
379	Morus007054.p1	NADPH-dependent thioredoxin reductase 1	4	39	0.17	39165	redox
380	Morus001993.p1	Aconitate hydratase 2, mitochondrial	22	167	0.16	102920	TCA
381	Morus010046.p1	Endo-1,3;1,4-beta-D-glucanase	6	122	0.16	57458	miscellaneousellaneous
382	Morus014800.p1	Heat shock 70 kDa protein, mitochondrial	5	96	0.16	71853	stress

383	Morus021702.p1	3-oxoacyl-[acyl-carrier-protein] synthase I, chloroplastic	10	205	0.16	49092	lipid metabolism
384	Morus003911.p1	ATP-dependent Clp protease proteolytic subunit-related protein 3	5	141	0.16	40882	protein
385	Morus024555.p1	Poly(rC)-binding protein 3	4	152	0.16	48844	RNA
386	Morus014011.p1	Probable glycerophosphoryl diester phosphodiesterase 2	6	295	0.16	81816	lipid metabolism
387	Morus001599.p1	Conserved hypothetical protein	6	67	0.16	47820	not assigned
388	Morus026409.p1	Chlorophyll a-b binding protein CP29.3, chloroplastic	5	86	0.15	30586	photosynthesis
389	Morus000761.p1	Pentatricopeptide repeat-containing protein	12	114	0.15	92406	not assigned
390	Morus024368.p1	Isocitrate dehydrogenase [NAD] catalytic subunit 5	4	89	0.15	41620	TCA
391	Morus010797.p1	ABC transporter I family member 11, chloroplastic	6	35	0.15	30483	transport
392	Morus010382.p1	Cucumisin	20	285	0.15	158227	protein
393	Morus017351.p1	Serine carboxypeptidase-like 50	5	55	0.15	49604	protein
394	Morus008123.p1	IAA-amino acid hydrolase ILR1-like 5	5	89	0.15	47707	hormone metabolism
395	Morus026532.p1	6-phosphogluconate dehydrogenase, decarboxylating	6	111	0.15	53844	OPP
396	Morus005911.p1	Formamidase	7	62	0.15	50475	miscellaneousellaneous
397	Morus026663.p1	Lysosomal alpha-mannosidase	18	368	0.15	114282	miscellaneousellaneous
398	Morus015818.p1	Probable glucan endo-1,3-beta-glucosidase A6	3	111	0.15	52145	miscellaneousellaneous
399	Morus019003.p1	Elongation factor G, chloroplastic	13	148	0.15	86851	protein
400	Morus011915.p1	Fatty acid hydroperoxide lyase	6	119	0.15	54855	miscellaneousellaneous
401	Morus026095.p1	Dipeptidyl peptidase family member 6	6	91	0.15	43551	protein
402	Morus022055.p1	50S ribosomal protein L4, chloroplastic	4	74	0.14	32054	protein
403	Morus024380.p1	Ribonucleoprotein	3	65	0.14	31292	RNA
404	Morus018635.p1	Conserved hypothetical protein	2	52	0.14	45158	not assigned
405	Morus024141.p1	Beta-D-xylosidase 4	6	187	0.14	84604	cell wall
406	Morus013374.p1	Proteasome subunit beta type-3-A	8	370	0.14	80974	protein
407	Morus022626.p1	FKBP-type peptidyl-prolyl cis-trans isomerase 1,	9	209	0.14	87839	protein
408	Morus025738.p1	Haloalkane dehalogenase	3	96	0.14	43640	not assigned
409	Morus006819.p1	Pectinesterase/pectinesterase inhibitor 34	7	47	0.14	65972	cell wall
410	Morus024051.p1	ATP-dependent Clp protease proteolytic subunit-related protein 4, chloroplastic	2	48	0.14	33495	protein
411	Morus009738.p1	ATP-dependent Clp protease proteolytic subunit 5	4	146	0.13	34203	protein
412	Morus027558.p1	Cinnamyl alcohol dehydrogenase 1	10	100	0.13	35081	secondary metabolism
413	Morus011664.p1	L-ascorbate oxidase homolog	4	53	0.13	60522	not assigned

414	Morus024806.p1	PsbP domain-containing protein 3, chloroplastic	5	62	0.13	34574	photosynthesis
415	Morus027553.p1	Gibberellin receptor GID1L3	5	92	0.13	35038	not assigned
416	Morus000839.p1	Photosystem II D2 protein	4	173	0.13	34260	photosynthesis
417	Morus005714.p1	Glutathione S-transferase omega	7	100	0.13	47998	miscellaneousellaneous
418	Morus027277.p1	WD-40 repeat-containing protein MSI4	5	42	0.13	58927	development
419	Morus011111.p1	Epoxide hydrolase 2	2	118	0.13	36070	miscellaneousellaneous
420	Morus000583.p1	Red chlorophyll catabolite reductase, chloroplastic	6	62	0.13	36126	stress
421	Morus018475.p1	Peroxidase 54	5	128	0.13	36921	miscellaneousellaneous
422	Morus007148.p1	Aspartic proteinase nepenthesin-2	4	50	0.13	46862	RNA
423	Morus001109.p1	Glutamine synthetase nodule isozyme	8	116	0.13	35940	N-metabolism
424	Morus011888.p1	Thiazole biosynthetic enzyme, chloroplastic	6	92	0.12	37455	Co-factor and vitamine metabolism
425	Morus016733.p1	Apospory-associated protein C	2	78	0.12	37446	minor CHO metabolism
426	Morus025925.p1	Alpha-glucosidase	6	207	0.12	93365	miscellaneousellaneous
427	Morus012627.p1	LRR receptor-like serine/threonine-protein kinase FLS2	4	86	0.12	53876	stress
428	Morus006929.p1	Eukaryotic translation initiation factor 2 subunit alpha	3	34	0.12	39146	protein
429	Morus019051.p1	L-lactate dehydrogenase A	7	55	0.12	37755	fermentation
430	Morus023189.p1	Carotenoid cleavage dioxygenase 4, chloroplastic	4	81	0.12	66020	hormone metabolism
431	Morus016314.p1	Bifunctional nitrilase/nitrile hydratase NIT4B	2	124	0.12	38067	secondary metabolism
432	Morus023909.p1	Uncharacterized protein	7	123	0.12	65250	protein
433	Morus016033.p1	Bifunctional 3-phosphoadenosine 5-phosphosulfate synthetase 2	6	41	0.11	54015	S-assimilation
434	Morus004124.p1	Serine hydroxymethyltransferase, mitochondrial	3	80	0.11	56893	C1-metabolism
435	Morus007545.p1	Porphobilinogen deaminase, chloroplastic	4	68	0.11	41353	tetrapyrrole synthesis
436	Morus016271.p1	Elongation factor 2	14	106	0.11	99403	protein
437	Morus008435.p1	Xylulose kinase	3	79	0.11	43714	minor CHO metabolism
438	Morus003768.p1	Nitrilase homolog 2-A	5	84	0.11	41611	not assigned
439	Morus004376.p1	Chaperone surA	7	83	0.11	42981	miscellaneousellaneous
440	Morus012145.p1	(+)-neomenthol dehydrogenase	4	129	0.10	62178	miscellaneousellaneous
441	Morus011320.p1	Enolase-phosphatase E1	2	102	0.10	59956	minor CHO metabolism
442	Morus012451.p1	Trigger factor	12	34	0.10	61352	protein
443	Morus001759.p1	Expansin-B2	4	111	0.10	62061	miscellaneousellaneous
444	Morus011294.p1	Pyruvate, phosphate dikinase, chloroplastic	19	111	0.10	106396	gluconeogenese
445	Morus003683.p1	ATP-dependent Clp protease proteolytic subunit-related protein	3	80	0.10	45567	protein

446	Morus009342.p1	Adenylosuccinate synthetase, chloroplastic	4	46	0.10	46710	nucleotide metabolism
447	Morus004691.p1	Calnexin homolog	2	86	0.10	62734	signalling
448	Morus013629.p1	Fumarylacetoacetase	5	109	0.10	47073	amino acid metabolism
449	Morus024527.p1	Vegetative cell wall protein gp1	3	144	0.10	47353	not assigned
450	Morus004531.p1	Beta-galactosidase 1	8	197	0.10	94371	miscellaneousellaneous
451	Morus007660.p1	4-hydroxy-3-methylbut-2-en-1-yl diphosphate synthase	16	72	0.10	108178	secondary metabolism
452	Morus005609.p1	GTP-binding nuclear protein Ran/TC4	3	92	0.10	64460	not assigned
453	Morus020169.p1	Aminotransferase ybdL	3	69	0.09	50948	secondary metabolism
454	Morus026175.p1	97 kDa heat shock protein	9	113	0.09	95518	stress
455	Morus022146.p1	Chaperone protein dnaJ	6	77	0.09	48298	stress
456	Morus004390.p1	Esterase	4	91	0.09	48462	miscellaneousellaneous
457	Morus020326.p1	Rab GDP dissociation inhibitor alpha	10	55	0.09	51270	signalling
458	Morus013185.p1	33 kDa ribonucleoprotein, chloroplastic	8	105	0.09	68806	not assigned
459	Morus004948.p1	NADH-ubiquinone oxidoreductase 75 kDa subunit	5	88	0.09	82539	mitochondrial electron transport
460	Morus015017.p1	1-deoxy-D-xylulose 5-phosphate reductoisomerase	3	73	0.09	51672	secondary metabolism
461	Morus017250.p1	Protein phosphatase 2C 38	4	122	0.09	72176	protein
462	Morus008067.p1	Aspartic proteinase	11	96	0.08	56778	protein
463	Morus003223.p1	Dihydrolipoyllysine-residue acetyltransferase	7	67	0.08	56317	TCA
464	Morus002148.p1	Vacuolar amino acid transporter 1	7	31	0.08	46012	transport
465	Morus006551.p1	NAD-dependent malic enzyme 59 kDa isoform	9	89	0.08	76993	TCA
466	Morus002684.p1	Aspartyl aminopeptidase	6	159	0.08	56319	protein
467	Morus013137.p1	Glycyl-tRNA synthetase 1, mitochondrial	9	97	0.08	77821	protein
468	Morus027550.p1	Presequence protease 2	14	43	0.08	114795	protein
469	Morus025629.p1	Heme-binding-like protein	2	53	0.07	59316	redox
470	Morus027347.p1	Protein Brevis radix-like 4	6	89	0.07	58739	not assigned
471	Morus003590.p1	Cysteine-rich receptor-like protein kinase 2	6	94	0.07	64741	signalling
472	Morus014247.p1	Prolyl-tRNA synthetase	2	41	0.07	65654	protein
473	Morus016023.p1	Subtilisin	5	120	0.06	71290	protein
474	Morus011017.p1	Microtubule-associated protein TORTIFOLIA1	8	47	0.06	71851	not assigned
475	Morus023897.p1	NAD-dependent malic enzyme 62 kDa isoform	4	83	0.06	73839	TCA
476	Morus022056.p1	Thiamine biosynthesis protein	9	39	0.06	72895	Co-factor and vitamine metabolism
477	Morus007324.p1	DEAD-box ATP-dependent RNA helicase 37	3	60	0.06	66427	RNA

478	Morus002253.p1	Polyadenylate-binding protein 2	11	120	0.06	70009	RNA
479	Morus021701.p1	DNA replication licensing factor MCM3 homolog	8	38	0.06	123515	DNA
480	Morus023653.p1	2-deoxyglucose-6-phosphate phosphatase, putative	8	126	0.05	83279	not assigned
481	Morus003998.p1	Serine carboxypeptidase-like 7	3	31	0.05	86547	protein
482	Morus010734.p1	NADP-dependent malic enzyme	5	113	0.05	81998	TCA
483	Morus025784.p1	Phospholipase D alpha 1	4	47	0.05	92059	lipid metabolism
484	Morus025292.p1	Protein TOC75-3, chloroplastic	3	91	0.05	90429	protein
485	Morus007961.p1	Hypothetical protein	7	62	0.05	95561	not assigned
486	Morus002045.p1	Methylcrotonoyl-CoA carboxylase subunit alpha	6	30	0.04	128681	amino acid metabolism
487	Morus017204.p1	Copper-transporting ATPase PAA1	5	75	0.04	101020	transport
488	Morus008310.p1	Receptor protein kinase ZmPK1	7	146	0.04	135647	miscellaneousellaneous
489	Morus024576.p1	GDP-mannose 3,5-epimerase 1	20	135	0.04	241773	RNA
490	Morus021444.p1	Protein tas	10	196	0.04	251110	cell
491	Morus003653.p1	Elongation factor Ts	10	66	0.04	115475	protein
492	Morus020407.p1	T-complex protein 1 subunit gamma	5	45	0.03	153297	protein

[a] Protein ID, according to the Morus database; [b] M.P., number of matched peptides; [c] Mol (%), protein abundance; [d] Function, function categorized using MapMan bin codes; redox, redox ascorbate/glutathione metabolism/dismutases/peroxiredoxin; protein, protein synthesis/ assembly/folding/degradation; TCA, tricarboxylic acid cycle; cell, cell organization/vesicle transport; RNA, RNA processing/regulation of transcription; and OPP, oxidative pentose phosphate.

Supplemental Table S2. Proteins Identified in the Branch of *Morus* by Gel-free/Label-free Proteomic Analysis

No.	Protein ID [a]	Description	M.P. [b]	Score	Mol (%) [c]	Mass (Da)	Function [d]
1	Morus025426.p1	Glutathione S-transferase DHAR1, mitochondrial	76	1152	3.95	26882	redox
2	Morus003561.p1	Enolase	192	3313	3.01	48199	glycolysis
3	Morus009492.p1	Macrophage migration inhibitory factor homolog	11	175	2.43	8961	not assigned
4	Morus001850.p1	Histone H4	15	188	2.40	11402	cell
5	Morus009000.p1	60S acidic ribosomal protein P2B	18	336	2.32	11673	protein
6	Morus000135.p1	Lipoxygenase 1	10	449	2.22	7922	hormone metabolism
7	Morus003013.p1	Phosphoglycerate kinase, cytosolic	72	1192	2.17	42729	glycolysis
8	Morus009016.p1	Isoflavone reductase homolog	101	2384	2.16	34553	secondary metabolism
9	Morus022108.p1	Annexin D2	52	894	2.06	36262	cell
10	Morus020498.p1	Quinone oxidoreductase-like protein	57	636	1.99	33732	miscellaneousellaneous
11	Morus015082.p1	Auxin-repressed 12.5 kDa protein	10	332	1.99	13355	development
12	Morus014845.p1	Oxygen-evolving enhancer protein 3-2, chloroplastic	49	939	1.97	24703	photosynthesis
13	Morus023628.p1	Tubulin beta-1 chain	67	1231	1.83	51015	cell
14	Morus013807.p1	Fructose-bisphosphate aldolase, cytoplasmic isozyme	53	1227	1.82	38459	glycolysis
15	Morus018316.p1	Superoxide dismutase 1 copper chaperone	6	149	1.69	11171	metal handling
16	Morus010743.p1	Triosephosphate isomerase, cytosolic	43	563	1.66	27548	glycolysis
17	Morus011742.p1	Oxygen-evolving enhancer protein 1, chloroplastic	61	947	1.59	35257	photosynthesis
18	Morus026837.p1	Serine hydroxymethyltransferase 1	55	803	1.57	52275	C1-metabolism
19	Morus018565.p1	Isoflavone reductase homolog	106	2284	1.55	33825	secondary metabolism
20	Morus023908.p1	Uncharacterized protein	72	1381	1.55	57888	protein
21	Morus003011.p1	Phosphoglycerate kinase, chloroplastic	57	777	1.53	49827	photosynthesis
22	Morus001936.p1	Peroxiredoxin-2B	71	383	1.48	17391	redox
23	Morus025862.p1	ATP synthase subunit beta, mitochondrial	81	1331	1.43	59400	mitochondrial electron transport
24	Morus014126.p1	ATP synthase subunit d, mitochondrial	20	231	1.42	19705	mitochondrial electron transport
25	Morus022535.p1	60S ribosomal protein L30	6	190	1.40	12651	protein
26	Morus014360.p1	Endochitinase 1	35	898	1.34	35679	stress
27	Morus008276.p1	Patatin group J-1	114	1532	1.33	43407	development
28	Morus002144.p1	ATP synthase subunit beta, chloroplastic	28	525	1.29	40899	photosynthesis
29	Morus001961.p1	Peroxidase 12	33	768	1.29	38426	miscellaneousellaneous
30	Morus014032.p1	NADH dehydrogenase [ubiquinone] 1 alpha subcomplex subunit 2	4	202	1.28	11288	mitochondrial electron transport

31	Morus025981.p1	Tubulin beta-3 chain	56	847	1.28	50624	cell
32	Morus008669.p1	Allene oxide cyclase 2, chloroplastic	26	568	1.24	27569	hormone metabolism
33	Morus021008.p1	40S ribosomal protein	15	125	1.21	15938	protein
34	Morus000548.p1	60S ribosomal protein L22-2	8	91	1.20	14085	protein
35	Morus007901.p1	Actin-7	47	765	1.20	41897	cell
36	Morus004086.p1	20 kDa chaperonin, chloroplastic	20	438	1.19	26730	protein
37	Morus013312.p1	Oxygen-evolving enhancer protein 2, chloroplastic	21	357	1.18	28487	photosynthesis
38	Morus000148.p1	S-formylglutathione hydrolase	11	159	1.18	18681	C1-metabolism
39	Morus018564.p1	Isoflavone reductase homolog P3	88	2104	1.17	45171	secondary metabolism
40	Morus011198.p1	L-ascorbate peroxidase, cytosolic	46	281	1.13	27414	redox
41	Morus022525.p1	Calmodulin	20	708	1.12	16894	signalling
42	Morus014092.p1	Subtilisin-like protease	109	1926	1.12	82582	protein
43	Morus004273.p1	Cysteine proteinase inhibitor 5	6	122	1.11	12559	protein
44	Morus006912.p1	Glyceraldehyde-3-phosphate dehydrogenase, cytosolic	110	2995	1.10	38377	glycolysis
45	Morus002874.p1	Leucine aminopeptidase 3, chloroplastic	43	960	1.09	60563	protein
46	Morus016168.p1	MLP-like protein 329	10	103	1.08	17320	stress
47	Morus006026.p1	17.5 kDa class I heat shock protein	25	188	1.08	17983	stress
48	Morus002575.p1	Small heat shock protein, chloroplastic	113	2298	1.04	26610	stress
49	Morus007342.p1	Peroxiredoxin-2F, mitochondrial	12	242	1.03	22580	redox
50	Morus014647.p1	Ribulose bisphosphate carboxylase small chain, chloroplastic	35	567	1.02	20671	photosynthesis
51	Morus014994.p1	Aldehyde dehydrogenase family 2 member B4, mitochondrial	27	608	1.00	59755	fermentation
52	Morus024951.p1	Triosephosphate isomerase, chloroplastic	29	406	0.99	34813	photosynthesis
53	Morus001657.p1	6-phosphogluconolactonase 4, chloroplastic	24	502	0.97	35151	OPP
54	Morus020362.p1	NADP-dependent oxidoreductase P2	20	481	0.95	38288	miscellaneousellaneous
55	Morus003952.p1	Lipoxygenase homology domain-containing protein 1	27	511	0.95	21171	not assigned
56	Morus021433.p1	Malate dehydrogenase, cytoplasmic	25	669	0.95	35912	TCA
57	Morus013361.p1	Protein disulfide-isomerase	42	647	0.92	56492	redox
58	Morus026982.p1	Allene oxide synthase, chloroplastic	49	552	0.91	56861	hormone metabolism
59	Morus018939.p1	Peptide methionine sulfoxide reductase (Fragment)	7	193	0.91	21860	protein
60	Morus002613.p1	40S ribosomal protein S3-3	13	266	0.91	32001	protein
61	Morus019723.p1	Conserved hypothetical protein	6	113	0.90	14633	not assigned
62	Morus013363.p1	L-ascorbate peroxidase T, chloroplastic	22	384	0.88	45708	redox

63	Morus013506.p1	40S ribosomal protein S12	5	149	0.86	15220	protein
64	Morus005704.p1	60S ribosomal protein L23	14	259	0.86	15188	protein
65	Morus021346.p1	Protein notum homolog	29	744	0.85	43696	cell wall
66	Morus006935.p1	Ferritin-3, chloroplastic	48	335	0.84	29626	metal handling
67	Morus010016.p1	10 kDa chaperonin	7	65	0.83	10611	protein
68	Morus018688.p1	Malate dehydrogenase, mitochondrial	42	731	0.83	36695	TCA
69	Morus010676.p1	Superoxide dismutase [Mn], mitochondrial	12	202	0.82	26407	redox
70	Morus007784.p1	UTP-glucose-1-phosphate uridylyltransferase	44	652	0.80	76133	glycolysis
71	Morus007512.p1	Kiwellin	10	666	0.80	24050	not assigned
72	Morus015814.p1	Quinone-oxidoreductase homolog, chloroplastic	17	268	0.80	35511	miscellaneousellaneous
73	Morus003616.p1	Fructokinase-2	34	389	0.79	35370	major CHO metabolism
74	Morus014500.p1	Pistil-specific extensin-like protein	63	1012	0.79	32716	stress
75	Morus001634.p1	Nucleoside diphosphate kinase 1	11	79	0.78	16322	nucleotide metabolism
76	Morus024224.p1	40S ribosomal protein S10	14	297	0.78	24791	protein
77	Morus025843.p1	Fructose-bisphosphate aldolase 3, chloroplastic	15	520	0.76	42615	photosynthesis
78	Morus021001.p1	Alpha-1,4 glucan phosphorylase L isozyme	81	1486	0.76	111309	major CHO metabolism
79	Morus019101.p1	Patatin group M-2	17	363	0.75	48408	development
80	Morus012122.p1	Polyphenol oxidase, chloroplastic	53	1438	0.75	65528	protein
81	Morus021899.p1	Beta-galactosidase 3	77	1426	0.74	92114	miscellaneousellaneous
82	Morus028068.p1	Polygalacturonase inhibitor 1	14	299	0.73	37677	cell wall
83	Morus025517.p1	Tubulin alpha chain	28	569	0.72	49920	cell
84	Morus027025.p1	Cinnamyl alcohol dehydrogenase 1	27	234	0.72	39604	secondary metabolism
85	Morus024398.p1	V-type proton ATPase subunit G	14	262	0.70	12219	transport
86	Morus022530.p1	Elongation factor 1-beta 2	9	221	0.69	24218	protein
87	Morus007651.p1	60S ribosomal protein L12	8	128	0.69	17956	protein
88	Morus020532.p1	Glutaredoxin	4	93	0.69	15307	redox
89	Morus005071.p1	RuBisCO large subunit-binding protein subunit beta, chloroplastic	22	586	0.68	64352	protein
90	Morus002856.p1	Chlorophyll a-b binding protein 40, chloroplastic	26	732	0.67	28149	photosynthesis
91	Morus026327.p1	Heat shock cognate 70 kDa protein 1	37	752	0.67	71553	stress
92	Morus012243.p1	Unknown	2	138	0.67	8961	not assigned
93	Morus024292.p1	Glutathione peroxidase	10	129	0.66	18602	redox
94	Morus020701.p1	Photosystem I reaction center subunit N, chloroplastic	9	316	0.66	18618	photosynthesis

95	Morus019263.p1	Lipid binding protein	3	71	0.65	12923	miscellaneousellaneous
96	Morus018842.p1	2-Cys peroxiredoxin BAS1-like, chloroplastic	20	241	0.65	29121	redox
97	Morus018664.p1	Carboxyvinyl-carboxyphosphonate phosphorylmutase	25	708	0.64	32039	not assigned
98	Morus000169.p1	GPI-anchored protein	10	299	0.64	19083	not assigned
99	Morus014354.p1	Expansin-like A1	8	214	0.64	28742	cell wall
100	Morus011207.p1	Selenium-binding protein	20	317	0.63	55119	metal handling
101	Morus000836.p1	Ribulose bisphosphate carboxylase large chain (Fragment)	64	1200	0.63	61599	photosynthesis
102	Morus022674.p1	Rhicadhesin receptor	18	431	0.63	23014	stress
103	Morus001377.p1	Stromal 70 kDa heat shock-related protein, chloroplastic	30	633	0.63	75481	stress
104	Morus017402.p1	Guanine nucleotide-binding protein subunit beta-like protein	14	140	0.63	36552	development
105	Morus001516.p1	Mitochondrial outer membrane protein porin of 34 kDa	13	323	0.62	29598	transport
106	Morus015899.p1	Alcohol dehydrogenase class-3	29	421	0.62	43063	miscellaneousellaneous
107	Morus018049.p1	Lactoylglutathione lyase	20	331	0.62	33175	biodegradation of xenobiotics
108	Morus014304.p1	Plastocyanin, chloroplastic	8	154	0.62	16620	photosynthesis
109	Morus002680.p1	Adenylate kinase B	13	285	0.62	26642	nucleotide metabolism
110	Morus019089.p1	Tubulin alpha-3/alpha-5 chain	25	279	0.61	50214	cell
111	Morus020052.p1	Surface protein	7	219	0.61	19872	not assigned
112	Morus022337.p1	Ferredoxin-3, chloroplastic	6	220	0.60	16962	OPP
113	Morus021269.p1	Uncharacterized protein	14	229	0.59	34892	not assigned
114	Morus004996.p1	Flavoprotein wrbA	7	255	0.59	20275	lipid metabolism
115	Morus006184.p1	Cysteine synthase	21	130	0.59	34400	amino acid metabolism
116	Morus010853.p1	Glutelin type-B 5	10	316	0.58	38656	development
117	Morus004201.p1	Universal stress protein A-like protein	4	208	0.58	18591	stress
118	Morus012628.p1	DNA-damage-repair/toleration protein DRT100	29	714	0.56	53868	stress
119	Morus022430.p1	Proteasome subunit beta type-1	9	201	0.56	24861	protein
120	Morus007268.p1	Aspartate aminotransferase, chloroplastic	18	307	0.56	50775	amino acid metabolism
121	Morus003426.p1	UPF0189 protein XAC3343	6	325	0.56	21275	not assigned
122	Morus004111.p1	Calreticulin	20	611	0.56	50196	signalling
123	Morus022005.p1	Peptidyl-prolyl cis-trans isomerase CYP20-1	26	414	0.56	22112	cell
124	Morus008757.p1	40S ribosomal protein S25	8	229	0.55	18057	protein
125	Morus008983.p1	Uncharacterized protein	14	336	0.55	10516	not assigned
126	Morus000672.p1	60S ribosomal protein L9	21	142	0.54	21870	protein

127	Morus024979.p1	Glycine-rich protein 2	14	169	0.54	18605	RNA
128	Morus006170.p1	DNA-damage-repair/toleration protein DRT102	16	184	0.53	33933	DNA
129	Morus011296.p1	Uncharacterized protein	2	56	0.52	15381	RNA
130	Morus024735.p1	Proteasome subunit alpha type-5	13	250	0.51	26997	protein
131	Morus012437.p1	40S ribosomal protein S5 (Fragment)	16	253	0.51	22844	protein
132	Morus008123.p1	IAA-amino acid hydrolase ILR1-like 5	26	229	0.50	47707	hormone metabolism
133	Morus010823.p1	REF/SRPP-like protein	6	36	0.50	27896	not assigned
134	Morus027200.p1	Glutathione S-transferase	20	275	0.50	40039	redox
135	Morus015372.p1	Granule-bound starch synthase 1, chloroplastic/amyloplastic	34	266	0.49	68120	major CHO metabolism
136	Morus020339.p1	Desiccation protectant protein Lea14 homolog	15	173	0.49	40399	development
137	Morus002213.p1	Peroxidase 4	14	167	0.49	35498	miscellaneousellaneous
138	Morus016604.p1	(3R)-hydroxymyristoyl-[acyl-carrier-protein] dehydratase	4	132	0.49	23809	lipid metabolism
139	Morus017475.p1	Peptidyl-prolyl cis-trans isomerase CYP20-2, chloroplastic	9	341	0.48	28281	cell
140	Morus021750.p1	Auxin-induced protein	12	196	0.48	40671	hormone metabolism
141	Morus009210.p1	60S acidic ribosomal protein P3-2	4	80	0.48	12022	protein
142	Morus007983.p1	Lipoxygenase 2, chloroplastic	32	600	0.48	102865	hormone metabolism
143	Morus018475.p1	Peroxidase 54	21	486	0.47	36921	miscellaneousellaneous
144	Morus007366.p1	Alpha-1,4-glucan-protein synthase	11	314	0.47	41966	cell wall
145	Morus006467.p1	Intracellular protease 1	19	73	0.46	42514	not assigned
146	Morus001808.p1	L-ascorbate peroxidase 2, cytosolic	27	197	0.46	21059	redox
147	Morus008822.p1	Glutamate-1-semialdehyde 2,1-aminomutase 1, chloroplastic	14	239	0.46	51141	tetrapyrrole synthesis
148	Morus016650.p1	Auxin-induced protein PCNT115	8	237	0.46	24910	hormone metabolism
149	Morus025502.p1	Cytochrome c	3	38	0.46	12423	mitochondrial electron transport
150	Morus009365.p1	5-methyltetrahydropteroyltriglutamate--homocysteine methyltransferase	22	581	0.46	84904	amino acid metabolism
151	Morus008428.p1	Formate dehydrogenase, mitochondrial	18	116	0.46	42456	C1-metabolism
152	Morus007711.p1	Aspartic proteinase nepenthesin-1	13	505	0.45	47654	RNA
153	Morus014667.p1	Alpha-xylosidase	45	591	0.45	103539	miscellaneousellaneous
154	Morus005567.p1	40S ribosomal protein S2-4	20	287	0.45	30047	protein
155	Morus007352.p1	Stem-specific protein TSJT1	6	258	0.45	25521	metal handling
156	Morus015229.p1	Eukaryotic translation initiation factor 5A-2	20	172	0.44	17698	protein
157	Morus013867.p1	S-adenosylmethionine synthetase 2	7	298	0.44	43654	amino acid metabolism
158	Morus011604.p1	Nucleosome-remodeling factor subunit BPTF	25	57	0.44	17601	not assigned

159	Morus002800.p1	Phosphoglucomutase, chloroplastic	21	287	0.44	65708	glycolysis
160	Morus016271.p1	Elongation factor 2	35	618	0.44	99403	protein
161	Morus026660.p1	Proteasome subunit alpha type-7	10	245	0.44	26129	protein
162	Morus022592.p1	Thaumatin-like protein 1a	4	119	0.44	26984	stress
163	Morus025073.p1	Ribosome-recycling factor, chloroplastic	7	280	0.44	35025	cell
164	Morus001906.p1	Disulfide-isomerase A6	6	103	0.43	40439	redox
165	Morus024765.p1	Photosystem II CP43 chlorophyll apoprotein	7	105	0.43	18044	photosynthesis
166	Morus024185.p1	Basic blue protein	5	117	0.42	13345	miscellaneous
167	Morus004803.p1	Ketol-acid reductoisomerase, chloroplastic	11	384	0.42	46064	amino acid metabolism
168	Morus023906.p1	Protein tolB	29	468	0.41	83312	not assigned
169	Morus013051.p1	Adenosine kinase 2	14	138	0.41	37797	nucleotide metabolism
170	Morus011938.p1	Dihydrolipoyllysine-residue succinyltransferase	15	143	0.41	46499	TCA
171	Morus021351.p1	60S ribosomal protein L11-2	4	164	0.41	23571	protein
172	Morus025037.p1	Glutathione peroxidase 5	11	162	0.40	19122	redox
173	Morus014113.p1	40S ribosomal protein S20-2	6	160	0.40	13838	protein
174	Morus004891.p1	Splicing factor, arginine/serine-rich 19	3	59	0.40	23705	stress
175	Morus025296.p1	Ribulose bisphosphate carboxylase/oxygenase activase 1, chloroplastic	12	334	0.40	52076	photosynthesis
176	Morus005874.p1	Profilin-3	5	164	0.39	14233	cell
177	Morus003800.p1	V-type proton ATPase catalytic subunit A	27	393	0.39	68994	transport
178	Morus015202.p1	Uncharacterized protein	15	250	0.39	33994	not assigned
179	Morus022215.p1	Cytochrome b6-f complex iron-sulfur subunit, chloroplastic	9	125	0.38	24759	photosynthesis
180	Morus000210.p1	Calvin cycle protein CP12	3	220	0.38	14542	photosynthesis
181	Morus026175.p1	97 kDa heat shock protein	39	661	0.38	95518	stress
182	Morus011664.p1	L-ascorbate oxidase homolog	24	326	0.37	60522	not assigned
183	Morus024265.p1	Aquaporin PIP1-3	9	140	0.37	30856	transport
184	Morus025784.p1	Phospholipase D alpha 1	24	549	0.37	92059	lipid metabolism
185	Morus003281.p1	Hypothetical protein	5	123	0.37	15053	stress
186	Morus010230.p1	Superoxide dismutase [Cu-Zn]	6	68	0.37	20420	redox
187	Morus019074.p1	Vegetative cell wall protein gp1	6	45	0.37	14951	not assigned
188	Morus026829.p1	Conserved hypothetical protein	2	95	0.37	14907	not assigned
189	Morus008661.p1	14-3-3 protein	30	704	0.37	81889	cell
190	Morus004247.p1	Forkhead box protein G1	5	186	0.36	15418	stress
191	Morus009634.p1	Proteasome subunit alpha type-2-B	9	129	0.36	25924	protein

192	Morus015920.p1	Photosystem I reaction center subunit IV B, chloroplastic	6	72	0.36	15399	photosynthesis
193	Morus016343.p1	Hypothetical protein	35	504	0.35	79098	not assigned
194	Morus014570.p1	Cell division cycle protein 48 homolog	33	740	0.35	96870	cell
195	Morus002073.p1	Ferredoxin-NADP reductase	9	140	0.34	33013	OPP
196	Morus001847.p1	Histone H2B	11	256	0.34	16166	DNA
197	Morus008883.p1	Uncharacterized protein	10	122	0.34	49487	signalling
198	Morus002489.p1	Nascent polypeptide-associated complex subunit alpha-like protein 1	16	238	0.34	22279	protein
199	Morus017207.p1	Proteasome subunit alpha type-4	4	180	0.34	27440	protein
200	Morus004210.p1	Glucan endo-1,3-beta-glucosidase, basic vacuolar isoform	10	136	0.34	39002	miscellaneous
201	Morus000089.p1	60S ribosomal protein	5	35	0.33	16487	protein
202	Morus003577.p1	40S ribosomal protein SA	4	62	0.33	33376	protein
203	Morus013778.p1	Monodehydroascorbate reductase	4	103	0.33	49982	redox
204	Morus007494.p1	RuBisCO large subunit-binding protein subunit alpha, chloroplastic	11	364	0.33	62000	photosynthesis
205	Morus007819.p1	Luminal-binding protein 5	17	516	0.33	73680	stress
206	Morus004691.p1	Calnexin homolog	10	122	0.33	62734	signalling
207	Morus010889.p1	NADP-dependent malic enzyme	53	534	0.32	113884	TCA
208	Morus008884.p1	Cysteine proteinase RD21a	7	283	0.32	52217	protein
209	Morus006808.p1	MFP1 attachment factor 1	3	107	0.32	16837	cell
210	Morus024220.p1	Monodehydroascorbate reductase, chloroplastic	16	300	0.32	53498	redox
211	Morus012024.p1	Proliferation-associated protein 2G4	13	95	0.32	47369	protein
212	Morus010327.p1	ADP,ATP carrier protein, mitochondrial	8	133	0.32	40544	transport
213	Morus024851.p1	Catalase isozyme 1	90	675	0.32	57208	redox
214	Morus003471.p1	Caffeic acid 3-O-methyltransferase	2	98	0.32	16921	secondary metabolism
215	Morus018265.p1	Peroxidase 3	45	887	0.32	64098	stress
216	Morus017723.p1	Chlorophyll a-b binding protein 8, chloroplastic	6	170	0.31	29521	photosynthesis
217	Morus011779.p1	Superoxide dismutase [Cu-Zn], chloroplastic	5	238	0.31	29603	redox
218	Morus014140.p1	Plastid-lipid-associated protein, chloroplastic	6	101	0.31	35137	cell
219	Morus007149.p1	Aspartic proteinase nepenthesin-2	14	414	0.31	52614	RNA
220	Morus001832.p1	Acetylcholinesterase	11	76	0.31	47515	miscellaneous
221	Morus000865.p1	Alcohol dehydrogenase 1	13	180	0.30	48703	fermentation
222	Morus001109.p1	Glutamine synthetase nodule isozyme	11	285	0.30	35940	N-metabolism
223	Morus016022.p1	SKP1-like protein 1B	4	115	0.30	17876	protein

224	Morus023007.p1	Uncharacterized protein	5	104	0.30	30644	development
225	Morus024842.p1	1,4-alpha-glucan-branching enzyme, chloroplastic/amyloplastic	15	185	0.30	86910	major CHO metabolism
226	Morus019087.p1	Mitochondrial 2-oxoglutarate/malate carrier protein	1	69	0.29	32224	transport
227	Morus025123.p1	Glutamate decarboxylase	25	542	0.29	56571	amino acid metabolism
228	Morus017382.p1	Calcium-binding protein	4	153	0.29	18705	signalling
229	Morus016063.p1	Peptidyl-prolyl cis-trans isomerase	44	316	0.29	18321	cell
230	Morus018550.p1	Glycine-rich RNA-binding protein GRP1A	6	115	0.29	18416	RNA
231	Morus006034.p1	18.5 kDa class I heat shock protein	11	222	0.29	18598	stress
232	Morus000338.p1	17.4 kDa class I heat shock protein	6	78	0.29	18282	stress
233	Morus026718.p1	Fructose-bisphosphate aldolase 2, chloroplastic	5	110	0.29	44113	photosynthesis
234	Morus011993.p1	Phosphoglucomutase, cytoplasmic	16	191	0.28	63757	glycolysis
235	Morus019004.p1	Heat shock protein STI	9	111	0.28	65100	stress
236	Morus023303.p1	Glycine cleavage system H protein 2, mitochondrial	3	79	0.28	18914	photosynthesis
237	Morus021898.p1	Thioredoxin H-type 1	13	191	0.28	19443	redox
238	Morus026150.p1	Dihydrolipoyl dehydrogenase 1, mitochondrial	15	313	0.28	53054	TCA
239	Morus016835.p1	Citrate synthase, mitochondrial	12	123	0.28	53248	TCA
240	Morus008166.p1	Proteasome subunit alpha type-6	13	228	0.27	33732	protein
241	Morus017847.p1	Ribonuclease	7	146	0.27	19960	RNA
242	Morus006195.p1	21 kDa protein	3	59	0.27	20062	miscellaneous
243	Morus017174.p1	Predicted protein	5	80	0.27	33060	signalling
244	Morus024003.p1	Cysteine proteinase inhibitor 12	3	36	0.27	26865	protein
245	Morus020098.p1	Small heat shock protein C2	8	188	0.26	34449	stress
246	Morus026532.p1	6-phosphogluconate dehydrogenase	11	216	0.26	53844	OPP
247	Morus002122.p1	Ribonuclease 3	2	108	0.26	20448	RNA
248	Morus018657.p1	Epidermis-specific secreted glycoprotein EP1	20	232	0.26	48950	miscellaneous
249	Morus002920.p1	Thioredoxin M-type 4, chloroplastic	4	136	0.26	20233	redox
250	Morus001779.p1	Transaldolase	5	269	0.26	48623	OPP
251	Morus020016.p1	Chlorophyll a-b binding protein 151, chloroplastic	2	79	0.25	28504	photosynthesis
252	Morus017117.p1	Hydroxyacylglutathione hydrolase cytoplasmic	1	45	0.25	28996	biodegradation of xenobiotics
253	Morus005609.p1	GTP-binding nuclear protein Ran/TC4	12	170	0.25	64460	not assigned
254	Morus025925.p1	Alpha-glucosidase	11	274	0.25	93365	miscellaneous
255	Morus016874.p1	Outer membrane lipoprotein blc	5	37	0.25	21306	transport

256	Morus011926.p1	Protein usf	16	92	0.25	29460	miscellaneous
257	Morus006217.p1	Importin subunit alpha-1	6	149	0.25	58914	protein
258	Morus001993.p1	Aconitate hydratase 2, mitochondrial	41	421	0.24	102920	TCA
259	Morus016710.p1	Disease resistance response protein 206	10	64	0.24	21649	stress
260	Morus023627.p1	Heme-binding-like protein	4	52	0.24	22207	not assigned
261	Morus013569.p1	Uncharacterized protein	7	101	0.24	44717	not assigned
262	Morus022764.p1	2,3-bisphosphoglycerate-independent phosphoglycerate mutase	21	239	0.24	61217	glycolysis
263	Morus001110.p1	Proline iminopeptidase	8	31	0.24	37855	protein
264	Morus004713.p1	Heat shock protein 101	34	617	0.23	101636	stress
265	Morus007809.p1	Isocitrate dehydrogenase	11	202	0.23	46429	TCA
266	Morus012656.p1	Chlorophyll a-b binding protein CP26, chloroplastic	11	38	0.22	39425	photosynthesis
267	Morus024998.p1	L-ascorbate peroxidase 3, peroxisomal	7	86	0.22	31854	redox
268	Morus006591.p1	Isocitrate dehydrogenase regulatory subunit 1, mitochondrial	6	178	0.22	40226	TCA
269	Morus012368.p1	Photosystem I reaction center subunit II, chloroplastic	10	54	0.22	23554	photosynthesis
270	Morus025778.p1	Cylicin-2	3	72	0.22	32881	RNA
271	Morus027774.p1	Quinone oxidoreductase	8	84	0.22	40725	miscellaneous
272	Morus027762.p1	Nucleoredoxin	11	119	0.22	65041	not assigned
273	Morus021663.p1	Conserved hypothetical protein	5	66	0.22	24281	not assigned
274	Morus004100.p1	Peroxiredoxin Q, chloroplastic	5	42	0.22	23684	redox
275	Morus010416.p1	UPF0664 stress-induced protein C29B12.11c	3	62	0.22	23524	not assigned
276	Morus007658.p1	Protein disulfide-isomerase 2	7	111	0.22	65186	redox
277	Morus013255.p1	Late embryogenesis abundant protein D-11	6	199	0.22	23457	stress
278	Morus019157.p1	Beta-fructofuranosidase, insoluble isoenzyme 1	24	369	0.22	88603	major CHO metabolism
279	Morus003542.p1	Cucumisin	11	277	0.22	81287	protein
280	Morus023055.p1	Pyruvate kinase, cytosolic isozyme	10	190	0.22	56922	glycolysis
281	Morus003021.p1	3-mercaptopyruvate sulfurtransferase	7	44	0.21	41188	amino acid metabolism
282	Morus018412.p1	Enoyl-[acyl-carrier-protein] reductase	7	53	0.21	41362	lipid metabolism
283	Morus003768.p1	Nitrilase homolog 2-A	10	157	0.21	41611	not assigned
284	Morus003263.p1	ATP-citrate synthase	4	93	0.21	24577	TCA
285	Morus022518.p1	Protein argonaute 4B	2	42	0.21	24941	RNA
286	Morus018853.p1	Ubiquitin-conjugating enzyme E2 variant 1C	5	68	0.21	25054	protein
287	Morus011651.p1	Glutathione S-transferase 6, chloroplastic	3	28	0.21	25064	miscellaneous

288	Morus027457.p1	Kynurenine formamidase	4	33	0.21	33244	DNA
289	Morus010420.p1	Soluble inorganic pyrophosphatase 1, chloroplastic	10	41	0.21	33355	nucleotide metabolism
290	Morus007590.p1	Ran-binding protein 1 homolog a	5	29	0.21	24980	signalling
291	Morus014388.p1	Fasciclin-like arabinogalactan protein 12	4	29	0.21	33689	cell wall
292	Morus009738.p1	ATP-dependent Clp protease proteolytic subunit 5, chloroplastic	10	182	0.21	34203	protein
293	Morus027367.p1	12-oxophytodienoate reductase 3	6	51	0.21	43271	hormone metabolism
294	Morus018536.p1	Fructose-bisphosphate aldolase 1, chloroplastic	24	85	0.21	42531	photosynthesis
295	Morus020326.p1	Rab GDP dissociation inhibitor alpha	9	108	0.21	51270	signalling
296	Morus026826.p1	Succinate dehydrogenase flavoprotein subunit 1, mitochondrial	3	127	0.21	69366	TCA
297	Morus024791.p1	Pyrophosphate--fructose 6-phosphate 1-phosphotransferase subunit alpha	12	148	0.20	70281	glycolysis
298	Morus014148.p1	Pyrophosphate--fructose 6-phosphate 1-phosphotransferase subunit beta	7	107	0.20	62016	glycolysis
299	Morus011845.p1	Multiple RNA-binding domain-containing protein 1	2	60	0.20	26121	RNA
300	Morus022685.p1	Uncharacterized protein	7	139	0.20	52791	not assigned
301	Morus003300.p1	NADP-dependent glyceraldehyde-3-phosphate dehydrogenase	18	66	0.20	54041	glycolysis
302	Morus017854.p1	Citrate-binding protein	4	46	0.20	35481	miscellaneous
303	Morus000876.p1	26S protease regulatory subunit S10B	5	100	0.20	44826	protein
304	Morus007483.p1	Predicted protein	8	84	0.19	36386	signalling
305	Morus024124.p1	Chlorophyll a-b binding protein CP24 10A, chloroplastic	3	134	0.19	27437	photosynthesis
306	Morus004398.p1	Serine protease inhibitor 6	6	152	0.19	26963	stress
307	Morus005113.p1	Beta-fructofuranosidase	12	166	0.19	64321	major CHO metabolism
308	Morus013333.p1	Stem-specific protein	2	54	0.19	27206	hormone metabolism
309	Morus007114.p1	Glycine-rich RNA-binding protein 2	4	241	0.19	27802	RNA
310	Morus013629.p1	Fumarylacetoacetase	6	86	0.18	47073	amino acid metabolism
311	Morus012593.p1	DnaJ protein homolog	5	116	0.18	47160	stress
312	Morus020913.p1	S-adenosylmethionine-dependent methyltransferase	7	130	0.18	38170	amino acid metabolism
313	Morus009848.p1	Thioredoxin-1	3	61	0.18	28633	redox
314	Morus022974.p1	Allene oxide cyclase 4, chloroplastic	3	27	0.18	28337	hormone metabolism
315	Morus017803.p1	Apolipoprotein D	13	42	0.18	38206	stress
316	Morus007054.p1	Thioredoxin reductase 2	4	55	0.18	39165	redox
317	Morus024406.p1	Fasciclin-like arabinogalactan protein 11	8	106	0.18	28231	cell wall
318	Morus007159.p1	Uncharacterized protein	3	139	0.18	28739	not assigned
319	Morus015784.p1	Low-temperature-induced 65 kDa protein	12	320	0.18	57985	stress

320	Morus018739.p1	Chlorophyll a-b binding protein 13, chloroplastic	2	83	0.18	28571	photosynthesis
321	Morus027934.p1	Alpha-L-arabinofuranosidase 1	11	87	0.18	69049	cell wall
322	Morus025018.p1	Elongation factor Tu, mitochondrial	8	126	0.17	51793	protein
323	Morus025582.p1	Transketolase, chloroplastic	13	154	0.17	80655	OPP
324	Morus000946.p1	GDSL esterase/lipase At5g03610	2	58	0.17	29578	miscellaneous
325	Morus011633.p1	DEAD-box ATP-dependent RNA helicase 56	9	94	0.17	59448	DNA
326	Morus013688.p1	Histidine-rich glycoprotein	7	115	0.17	41446	not assigned
327	Morus015818.p1	Glucan endo-1,3-beta-glucosidase A6	5	118	0.17	52145	miscellaneous
328	Morus018995.p1	FAM10 family protein	5	72	0.17	41923	not assigned
329	Morus021880.p1	Endoplasmin homolog	17	180	0.17	114221	stress
330	Morus006060.p1	V-type proton ATPase subunit B2	14	271	0.16	63333	transport
331	Morus014443.p1	Serpin-ZX	4	70	0.16	42391	protein
332	Morus022454.p1	Fasciclin-like arabinogalactan protein 8	11	225	0.16	43455	cell wall
333	Morus027409.p1	Aldehyde dehydrogenase family	8	61	0.16	65994	fermentation
334	Morus006848.p1	Protein SEC13 homolog	5	42	0.16	32870	protein
335	Morus009910.p1	60S ribosomal protein L18a	3	29	0.16	32699	protein
336	Morus004025.p1	Nicotinamide-nucleotide adenylyltransferase 2	9	186	0.16	31967	Co-factor and vitamine metabolism
337	Morus022871.p1	Xylose isomerase	9	53	0.16	54186	minor CHO metabolism
338	Morus003849.p1	60S ribosomal protein L4	18	571	0.15	44987	protein
339	Morus027470.p1	D-3-phosphoglycerate dehydrogenase, chloroplastic	7	67	0.15	44670	amino acid metabolism
340	Morus005931.p1	Succinyl-CoA ligase subunit beta, mitochondrial	9	131	0.15	45635	TCA
341	Morus007324.p1	DEAD-box ATP-dependent RNA helicase 37	5	29	0.15	66427	RNA
342	Morus026391.p1	60S acidic ribosomal protein	9	47	0.15	34186	protein
343	Morus003223.p1	Dihydrolipoyllysine-residue acetyltransferase	5	146	0.15	56317	TCA
344	Morus020380.p1	60S ribosomal protein L5	4	121	0.15	35074	protein
345	Morus009602.p1	Early nodulin-like protein 2	8	155	0.15	34674	miscellaneous
346	Morus002684.p1	Aspartyl aminopeptidase	13	161	0.15	56319	protein
347	Morus000029.p1	Photosystem Q(B) protein	2	34	0.15	34748	photosynthesis
348	Morus013359.p1	Delta-aminolevulinic acid dehydratase, chloroplastic	5	106	0.15	47067	tetrapyrrole synthesis
349	Morus009835.p1	Protein disulfide-isomerase A6	7	281	0.14	48711	redox
350	Morus017885.p1	Transcription factor RF2a	7	102	0.14	37053	cell
351	Morus017980.p1	26S protease regulatory subunit 7	6	46	0.14	48208	protein

352	Morus009361.p1	Proteoglycan 4	3	43	0.14	36829	stress
353	Morus025727.p1	NADH-ubiquinone oxidoreductase 24 kDa subunit, mitochondrial	5	64	0.14	36183	mitochondrial electron transport
354	Morus000168.p1	Uncharacterized oxidoreductase	6	57	0.14	36852	OPP
355	Morus001759.p1	Expansin-B2	12	220	0.14	62061	miscellaneous
356	Morus007739.p1	Beta-D-xylosidase 1	16	227	0.14	85868	cell wall
357	Morus017351.p1	Serine carboxypeptidase-like 50	4	67	0.14	49604	protein
358	Morus001781.p1	Photosystem II CP47 chlorophyll apoprotein	5	144	0.14	50773	photosynthesis
359	Morus009329.p1	Aminotransferase	7	55	0.13	51671	amino acid metabolism
360	Morus026663.p1	Lysosomal alpha-mannosidase	13	154	0.13	114282	miscellaneous
361	Morus017695.p1	31 kDa ribonucleoprotein, chloroplastic	5	87	0.13	38128	RNA
362	Morus013818.p1	Cytochrome c1-1, heme protein, mitochondrial	9	248	0.12	69151	mitochondrial electron transport
363	Morus007482.p1	Fumarate hydratase 1, mitochondrial	9	81	0.12	53693	TCA
364	Morus014011.p1	Glycerophosphoryl diester phosphodiesterase 2	5	203	0.12	81816	lipid metabolism
365	Morus026717.p1	Thymus-specific serine protease	9	54	0.12	53786	protein
366	Morus008306.p1	Patellin-3	4	56	0.12	58217	transport
367	Morus011151.p1	Malonyl CoA-acyl carrier protein transacylase, mitochondrial	2	117	0.12	43116	lipid metabolism
368	Morus026664.p1	26S proteasome non-ATPase regulatory subunit 4	5	47	0.12	43012	protein
369	Morus019413.p1	Cysteine proteinase 15A	9	187	0.12	41574	protein
370	Morus024368.p1	Isocitrate dehydrogenase catalytic subunit 5, mitochondrial	13	68	0.12	41620	TCA
371	Morus006886.p1	Glucose-1-phosphate adenylyltransferase small subunit 2, chloroplastic	6	105	0.12	57117	major CHO metabolism
372	Morus017397.p1	Beta-glucosidase 44	1	38	0.11	59118	miscellaneous
373	Morus027277.p1	WD-40 repeat-containing protein	3	130	0.11	58927	development
374	Morus019225.p1	Metacaspase-4	8	250	0.11	47736	protein
375	Morus020384.p1	Cysteine synthase, chloroplastic/chromoplastic	8	68	0.11	43997	amino acid metabolism
376	Morus014749.p1	Glutamate dehydrogenase 1	6	114	0.11	44436	N-metabolism
377	Morus006819.p1	Pectinesterase/pectinesterase inhibitor 34	13	168	0.11	65972	cell wall
378	Morus020994.p1	Chaperone protein	25	130	0.11	108156	stress
379	Morus027308.p1	Eukaryotic initiation factor 4A-15	14	297	0.10	96147	protein
380	Morus018677.p1	Uncharacterized protein	4	157	0.10	49861	not assigned
381	Morus012367.p1	Lipoxygenase 5, chloroplastic	8	242	0.10	100606	hormone metabolism
382	Morus026880.p1	Chaperonin	5	131	0.10	49295	protein
383	Morus019025.p1	Monocopper oxidase-like protein	2	64	0.10	66194	development

384	Morus024026.p1	Plasminogen activator inhibitor 1 RNA-binding protein	4	40	0.10	50388	RNA
385	Morus024964.p1	Amidase C869.01	9	52	0.10	47961	miscellaneous
386	Morus005911.p1	Formamidase	6	78	0.10	50475	miscellaneous
387	Morus026134.p1	ADP-ribosylation factor 1	6	148	0.10	50877	transport
388	Morus001167.p1	Seryl-tRNA synthetase	7	30	0.10	51656	protein
389	Morus008001.p1	Malate dehydrogenase [NADP], chloroplastic	3	51	0.10	49140	TCA
390	Morus024141.p1	Beta-D-xylosidase 4	5	88	0.10	84604	cell wall
391	Morus020103.p1	Gamma-glutamyltranspeptidase 1	9	55	0.10	66667	stress
392	Morus008304.p1	Patellin-2	12	49	0.09	72014	transport
393	Morus008067.p1	Aspartic proteinase	8	165	0.09	56778	protein
394	Morus022663.p1	Serine-rich adhesin for platelets	9	138	0.09	94637	not assigned
395	Morus010046.p1	Endo-1,3;1,4-beta-D-glucanase	3	103	0.09	57458	miscellaneous
396	Morus010076.p1	Glutathione reductase, cytosolic	4	116	0.09	54912	redox
397	Morus020926.p1	Citrate synthase, glyoxysomal	16	34	0.09	56879	gluconeogenese
398	Morus014786.p1	Methylmalonate-semialdehyde dehydrogenase	10	161	0.08	105060	amino acid metabolism
399	Morus007672.p1	Beta-galactosidase 8	10	90	0.08	98498	miscellaneous
400	Morus014298.p1	Hypothetical protein	3	99	0.08	60016	not assigned
401	Morus019523.p1	Far upstream element-binding protein 1	10	59	0.07	71479	RNA
402	Morus027674.p1	Alpha-galactosidase	8	94	0.07	69562	minor CHO metabolism
403	Morus007961.p1	Hypothetical protein	18	218	0.07	95561	not assigned
404	Morus017594.p1	Uncharacterized protein	3	72	0.07	96824	not assigned
405	Morus003360.p1	Protein VAC14 homolog	8	38	0.06	81973	not assigned
406	Morus004948.p1	NADH-ubiquinone oxidoreductase 75 kDa subunit, mitochondrial	7	57	0.06	82539	mitochondrial electron transport
407	Morus013957.p1	Eukaryotic initiation factor iso-4F subunit p82-34	9	38	0.05	90263	protein
408	Morus024833.p1	Glyceraldehyde-3-phosphate dehydrogenase B, chloroplastic	18	75	0.04	148833	photosynthesis
409	Morus009727.p1	Nodal modulator 1	8	45	0.04	131562	not assigned
410	Morus008507.p1	Sucrose-phosphate synthase 1	12	21	0.04	121085	major CHO metabolism
411	Morus024576.p1	GDP-mannose 3,5-epimerase 1	24	90	0.02	241773	RNA
412	Morus020837.p1	Extensin	8	44	-	84300	cell wall
413	Morus027760.p1	Pantothenate kinase 2	7	42	-	45114	Co-factor and vitamine metabolism
414	Morus027199.p1	Valyl-tRNA synthetase	12	39	-	80902	protein

[a] Protein ID, according to the Morus database; [b] M.P., number of matched peptides; [c] Mol (%), protein abundance; [d] Function, function categorized using MapMan bin codes; redox, redox ascorbate/glutathione metabolism/dismutases/peroxiredoxin; protein, cell, cell organization/cycle; protein synthesis/degradation/posttranslational modification; TCA, tricarboxylic acid cycle; RNA processing/regulation of transcription/binding; OPP, oxidative pentose phosphate; and DNA, DNA synthesis.

Supplemental Table S3. Proteins Identified in the Root of *Morus* by Gel-free/Label-free Proteomic Analysis

No.	Protein ID [a]	Description	M.P. [b]	Score	Mol (%) [c]	Mass (Da)	Function [d]
1	Morus026438.p1	Hypothetical protein isoform 2	10	272	4.04	11566	not assigned
2	Morus009492.p1	Macrophage migration inhibitory factor homolog	10	214	3.69	8961	not assigned
3	Morus001961.p1	Peroxidase 12	42	829	3.62	38426	miscellaneous
4	Morus003889.p1	Subtilisin-like protease	8	110	2.57	11007	not assigned
5	Morus018316.p1	Superoxide dismutase 1 copper chaperone	13	352	2.48	11171	metal handling
6	Morus009000.p1	60S acidic ribosomal protein P2B	17	320	2.32	11673	protein
7	Morus023628.p1	Tubulin beta-1 chain	47	620	1.99	51015	cell
8	Morus004699.p1	Unknown	15	172	1.94	23534	not assigned
9	Morus024735.p1	Proteasome subunit alpha type-5	26	510	1.87	26997	protein
10	Morus010016.p1	10 kDa chaperonin	10	102	1.84	10611	protein
11	Morus017847.p1	Ribonuclease	17	387	1.80	19960	RNA
12	Morus014362.p1	Endochitinase 1	33	755	1.77	35841	stress
13	Morus001850.p1	Histone H4	4	71	1.64	11402	cell
14	Morus023244.p1	Conserved hypothetical protein	7	218	1.63	14530	secondary metabolism
15	Morus019723.p1	Conserved hypothetical protein	9	240	1.62	14633	not assigned
16	Morus017207.p1	Proteasome subunit alpha type-4	22	378	1.58	27440	protein
17	Morus019878.p1	Proteasome subunit beta type-6	18	323	1.46	26634	protein
18	Morus022430.p1	Proteasome subunit beta type-1	18	293	1.44	24861	protein
19	Morus003952.p1	Lipoxygenase homology domain-containing protein 1	19	430	1.44	21171	not assigned
20	Morus022592.p1	Thaumatin-like protein 1a	26	654	1.41	26984	stress
21	Morus023332.p1	Serine/arginine repetitive matrix protein 1	5	155	1.37	10758	not assigned
22	Morus025483.p1	Steroid-binding protein 3	8	216	1.35	10872	redox
23	Morus022525.p1	Calmodulin	22	505	1.31	16894	signalling
24	Morus015082.p1	Auxin-repressed 12.5 kDa protein	8	153	1.30	13355	development
25	Morus003616.p1	Fructokinase-2	25	394	1.25	35370	major CHO metabolism
26	Morus005874.p1	Profilin-3	14	231	1.19	14233	cell
27	Morus022674.p1	Rhicadhesin receptor	22	284	1.14	23014	stress
28	Morus016057.p1	Tubulin beta-6 chain	38	455	1.13	50888	cell
29	Morus020498.p1	Quinone oxidoreductase	16	330	1.12	33732	miscellaneous
30	Morus004259.p1	Unknown	6	125	1.09	10814	not assigned

31	Morus017382.p1	Calcium-binding protein CML27	6	215	1.05	18705	signalling
32	Morus027137.p1	Pleckstrin homology domain	12	175	1.03	22842	lipid metabolism
33	Morus015139.p1	Regulator of ribonuclease-like protein 2	6	162	1.02	18062	C1-metabolism
34	Morus008669.p1	Allene oxide cyclase 2, chloroplastic	16	304	0.99	27569	hormone metabolism
35	Morus010743.p1	Triosephosphate isomerase, cytosolic	13	187	0.99	27548	glycolysis
36	Morus004210.p1	Glucan endo-1,3-beta-glucosidase, basic vacuolar isoform	52	287	0.94	39002	miscellaneous
37	Morus002936.p1	Flavoprotein	15	249	0.91	21844	lipid metabolism
38	Morus011779.p1	Superoxide dismutase [Cu-Zn], chloroplastic	20	454	0.90	29603	redox
39	Morus001936.p1	Peroxiredoxin-2B	17	163	0.90	17391	redox
40	Morus005497.p1	Elongation factor 1-delta 1	16	174	0.85	26235	protein
41	Morus002067.p1	Glycine-rich RNA-binding protein 2, mitochondrial	4	181	0.83	15592	RNA
42	Morus004201.p1	Universal stress protein A-like protein	6	236	0.82	18591	stress
43	Morus017423.p1	Glu S.griseus protease inhibitor	13	275	0.81	7768	stress
44	Morus000135.p1	Lipoxygenase 1	2	170	0.79	7922	hormone metabolism
45	Morus015337.p1	Agglutinin alpha chain	124	1898	0.79	16247	miscellaneous
46	Morus002674.p1	Mucin-5AC (Fragments)	8	192	0.78	16290	signalling
47	Morus001634.p1	Nucleoside diphosphate kinase 1	7	186	0.78	16322	nucleotide metabolism
48	Morus000706.p1	Ubiquitin-like protein SMT3	5	70	0.78	11206	protein
49	Morus022108.p1	Annexin D2	8	161	0.77	36262	cell
50	Morus003013.p1	Phosphoglycerate kinase, cytosolic	10	267	0.75	42729	glycolysis
51	Morus013777.p1	60S acidic ribosomal protein	4	50	0.75	11483	protein
52	Morus016701.p1	GPI-anchored protein	8	96	0.74	20065	lipid metabolism
53	Morus022337.p1	Ferredoxin-3, chloroplastic	13	466	0.74	16962	OPP
54	Morus028068.p1	Polygalacturonase inhibitor 1	11	213	0.73	37677	cell wall
55	Morus013807.p1	Fructose-bisphosphate aldolase	21	248	0.71	38459	glycolysis
56	Morus008941.p1	Germin-like protein subfamily T member 2	13	87	0.71	23695	stress
57	Morus002887.p1	Serine protease inhibitor 6	18	417	0.70	26906	stress
58	Morus020532.p1	Glutaredoxin	7	168	0.69	15307	redox
59	Morus023908.p1	Uncharacterized protein	21	396	0.69	57888	protein
60	Morus018658.p1	Epidermis-specific secreted glycoprotein	32	474	0.68	48987	miscellaneous
61	Morus019147.p1	Small nuclear ribonucleoprotein G	2	68	0.68	8830	RNA
62	Morus009630.p1	Hypothetical protein	19	434	0.68	42982	protein

63	Morus020213.p1	Heat shock cognate protein 80	28	254	0.67	83687	stress
64	Morus003694.p1	UPF0133 protein	4	108	0.67	19163	not assigned
65	Morus018475.p1	Peroxidase 54	36	599	0.67	36921	miscellaneous
66	Morus025517.p1	Tubulin alpha chain	18	308	0.66	49920	cell
67	Morus024979.p1	Glycine-rich protein 2	10	157	0.66	18605	RNA
68	Morus008830.p1	Mitochondrial import inner membrane translocase subunit Tim8	3	151	0.66	9120	protein
69	Morus018219.p1	Fasciclin-like arabinogalactan protein 1	28	353	0.66	44165	cell wall
70	Morus028063.p1	Polygalacturonase inhibitor	19	486	0.66	41106	cell wall
71	Morus002489.p1	Nascent polypeptide-associated complex subunit alpha-like protein 1	10	317	0.65	22279	protein
72	Morus000240.p1	Ras-related protein	5	124	0.64	18933	signalling
73	Morus013561.p1	Pathogenesis-related protein 1	5	154	0.64	19020	stress
74	Morus023442.p1	Protein SSXT	5	142	0.64	22743	RNA
75	Morus003542.p1	Cucumisin	30	824	0.63	81287	protein
76	Morus024103.p1	Mitochondrial import inner membrane translocase subunit	2	70	0.62	9637	protein
77	Morus014304.p1	Plastocyanin, chloroplastic	12	75	0.62	16620	photosynthesis
78	Morus020052.p1	Surface protein	14	300	0.61	19872	not assigned
79	Morus009633.p1	Glycine decarboxylase complex H-protein	7	125	0.61	16830	photosynthesis
80	Morus002122.p1	Ribonuclease 3	8	160	0.59	20448	RNA
81	Morus024265.p1	Aquaporin	13	188	0.59	30856	transport
82	Morus021001.p1	Alpha-1,4 glucan phosphorylase L isozyme	34	490	0.59	111309	major CHO metabolism
83	Morus008884.p1	Cysteine proteinase RD21a	26	527	0.58	52217	protein
84	Morus008307.p1	Hypothetical protein	3	40	0.58	10266	not assigned
85	Morus025133.p1	Cell wall protein	6	178	0.58	31344	not assigned
86	Morus018328.p1	NifU-like protein 4, mitochondrial	9	133	0.58	31854	protein
87	Morus022710.p1	Acyl-CoA-binding protein	5	93	0.56	10432	lipid metabolism
88	Morus015372.p1	Granule-bound starch synthase 1	17	268	0.56	68120	major CHO metabolism
89	Morus012741.p1	14-3-3-like protein	8	107	0.55	29214	signalling
90	Morus024957.p1	Peptidyl-prolyl cis-trans isomerase	4	69	0.54	18291	cell
91	Morus003561.p1	Enolase	28	203	0.54	48199	glycolysis
92	Morus025862.p1	ATP synthase subunit beta, mitochondrial	19	285	0.54	59400	mitochondrial electron transport
93	Morus026982.p1	Allene oxide synthase, chloroplastic	18	142	0.54	56861	hormone metabolism
94	Morus016711.p1	Disease resistance response protein 206	7	65	0.53	22057	stress

95	Morus019538.p1	UPF0587 protein	2	38	0.53	18810	not assigned
96	Morus001779.p1	Transaldolase	18	448	0.53	48623	OPP
97	Morus009738.p1	ATP-dependent Clp protease proteolytic subunit 5, chloroplastic	13	327	0.52	34203	protein
98	Morus007342.p1	Peroxiredoxin-2F, mitochondrial	5	81	0.52	22580	redox
99	Morus024571.p1	Pro-hevein	5	298	0.51	23009	stress
100	Morus025201.p1	Heat shock cognate 70 kDa protein	12	321	0.51	71553	stress
101	Morus009012.p1	Proteasome subunit alpha type-1-B	6	175	0.51	31190	protein
102	Morus014500.p1	Pistil-specific extensin	13	166	0.49	32716	stress
103	Morus004363.p1	40S ribosomal protein S13	3	78	0.49	16047	protein
104	Morus024406.p1	Fasciclin-like arabinogalactan protein 11	8	100	0.49	28231	cell wall
105	Morus009210.p1	60S acidic ribosomal protein P3-2	4	127	0.48	12022	protein
106	Morus017143.p1	Conserved hypothetical protein	3	38	0.48	16590	not assigned
107	Morus006727.p1	Glyceraldehyde-3-phosphate dehydrogenase, cytosolic	12	147	0.48	37065	glycolysis
108	Morus002073.p1	Ferredoxin--NADP reductase, chloroplastic	8	133	0.47	33013	OPP
109	Morus005596.p1	Lichenase	6	246	0.47	36954	miscellaneous
110	Morus007901.p1	Actin-7	13	101	0.47	41897	cell
111	Morus006808.p1	MFP1 attachment factor 1	5	84	0.46	16837	cell
112	Morus006581.p1	Ubiquilin	14	210	0.46	59047	protein
113	Morus016169.p1	MLP-like protein 328	2	76	0.46	17128	stress
114	Morus025502.p1	Cytochrome c	2	51	0.46	12423	mitochondrial electron transport
115	Morus028046.p1	Mitochondrial import inner membrane translocase subunit TIM22	5	33	0.46	17073	protein
116	Morus007983.p1	Lipoxygenase 2, chloroplastic	27	189	0.46	102865	hormone metabolism
117	Morus010987.p1	Endogenous alpha-amylase/subtilisin inhibitor	5	58	0.46	21443	stress
118	Morus003426.p1	UPF0189 protein	9	206	0.46	21275	not assigned
119	Morus004627.p1	Germin-like protein subfamily 3 member 4	3	126	0.46	25850	stress
120	Morus024122.p1	(3R)-hydroxymyristoyl-[acyl-carrier-protein] dehydratase	5	96	0.45	25770	lipid metabolism
121	Morus017991.p1	Pectinesterase 1	5	99	0.45	25563	cell wall
122	Morus007352.p1	Stem-specific protein TSJT1	8	222	0.45	25521	metal handling
123	Morus016168.p1	MLP-like protein 329	3	30	0.45	17320	stress
124	Morus013984.p1	Cationic peroxidase 1	10	177	0.45	34651	miscellaneous
125	Morus025167.p1	Glucan endo-1,3-beta-glucosidase 6	11	219	0.44	52451	miscellaneous
126	Morus019025.p1	Monocopper oxidase-like protein	25	437	0.44	66194	development

127	Morus010375.p1	Predicted protein	4	196	0.44	12773	miscellaneous
128	Morus026327.p1	Heat shock cognate 70 kDa protein 1	12	271	0.44	71553	stress
129	Morus002575.p1	Small heat shock protein, chloroplastic	8	186	0.43	26610	stress
130	Morus021433.p1	Malate dehydrogenase, cytoplasmic	9	303	0.43	35912	TCA
131	Morus024030.p1	GTP-binding protein	2	47	0.42	22822	signalling
132	Morus020343.p1	40S ribosomal protein SA	10	147	0.41	32102	protein
133	Morus019047.p1	14 kDa proline-rich protein	7	144	0.41	13554	miscellaneous
134	Morus007805.p1	Conserved hypothetical protein	2	51	0.41	23337	not assigned
135	Morus015952.p1	Malate dehydrogenase, chloroplastic	8	195	0.41	42106	TCA
136	Morus011888.p1	Thiazole biosynthetic enzyme, chloroplastic	14	271	0.40	37455	Co-factor and vitamine metabolism
137	Morus016678.p1	Isoaspartyl peptidase/L-asparaginase 1	4	175	0.40	33403	amino acid metabolism
138	Morus009848.p1	Thioredoxin-1	4	113	0.39	28633	redox
139	Morus022454.p1	Fasciclin-like arabinogalactan protein 8	27	296	0.39	43455	cell wall
140	Morus006184.p1	Cysteine synthase	5	157	0.39	34400	amino acid metabolism
141	Morus018842.p1	2-Cys peroxiredoxin	9	98	0.39	29121	redox
142	Morus008883.p1	Uncharacterized protein	20	250	0.38	49487	signalling
143	Morus003953.p1	Polycystic kidney disease protein 1-like 2	10	98	0.38	19765	not assigned
144	Morus020519.p1	50S ribosomal protein L12, chloroplastic	6	68	0.38	19940	protein
145	Morus000210.p1	Calvin cycle protein CP12	2	127	0.38	14542	photosynthesis
146	Morus002920.p1	Thioredoxin M-type 4, chloroplastic	5	147	0.37	20233	redox
147	Morus003281.p1	Hypothetical protein	4	84	0.37	15053	stress
148	Morus020187.p1	LysM domain-containing GPI-anchored protein 1	15	94	0.37	45278	not assigned
149	Morus018564.p1	Isoflavone reductase homolog P3	8	95	0.37	45171	secondary metabolism
150	Morus024842.p1	1,4-alpha-glucan-branching enzyme	29	290	0.37	86910	major CHO metabolism
151	Morus011225.p1	Superoxide dismutase [Mn], mitochondrial	8	90	0.37	25638	redox
152	Morus010227.p1	Acyl carrier protein 1, chloroplastic	2	139	0.36	15264	lipid metabolism
153	Morus009634.p1	Proteasome subunit alpha type-2-B	8	35	0.36	25924	protein
154	Morus018948.p1	Protein-L-isoaspartate O-methyltransferase	15	124	0.36	41607	protein
155	Morus011296.p1	Uncharacterized protein	4	55	0.36	15381	RNA
156	Morus018265.p1	Peroxidase 3	34	403	0.35	64098	stress
157	Morus011760.p1	Patatin-2-Kuras 3	4	217	0.35	31307	development
158	Morus013051.p1	Adenosine kinase 2	6	145	0.35	37797	nucleotide metabolism

159	Morus014908.p1	Glucan endo-1,3-beta-glucosidase 5	6	98	0.34	54117	miscellaneous
160	Morus000881.p1	Prefoldin subunit 5	4	101	0.33	16624	RNA
161	Morus023325.p1	Conserved hypothetical protein	4	222	0.33	28409	development
162	Morus024050.p1	Expansin	4	79	0.33	28457	cell wall
163	Morus009602.p1	Early nodulin-like protein 2	3	53	0.33	34674	miscellaneous
164	Morus000836.p1	Ribulose bisphosphate carboxylase large chain (Fragment)	14	193	0.33	61599	photosynthesis
165	Morus013768.p1	Ankyrin repeat domain	13	174	0.32	46613	RNA
166	Morus015202.p1	Uncharacterized protein	8	213	0.32	33994	not assigned
167	Morus015849.p1	Uncharacterized protein	3	43	0.32	16911	not assigned
168	Morus006165.p1	40S ribosomal protein S3a	7	44	0.31	30205	protein
169	Morus015229.p1	Eukaryotic translation initiation factor 5A-2	4	129	0.31	17698	protein
170	Morus024832.p1	Hypothetical protein	3	58	0.31	17287	not assigned
171	Morus014140.p1	Plastid-lipid-associated protein, chloroplastic	10	163	0.31	35137	cell
172	Morus007149.p1	Aspartic proteinase nepenthesin-2	22	512	0.31	52614	RNA
173	Morus012628.p1	DNA-damage-repair/toleration protein DRT100	10	132	0.31	53868	stress
174	Morus023982.p1	Aspartic proteinase nepenthesin-1	25	477	0.31	47201	RNA
175	Morus001832.p1	Acetylcholinesterase	12	153	0.31	47515	miscellaneous
176	Morus007710.p1	Universal stress protein A	9	119	0.30	18184	hormone metabolism
177	Morus007783.p1	Hypothetical protein	6	254	0.30	36763	not assigned
178	Morus026638.p1	SKP1-like protein 1A	5	120	0.30	18172	protein
179	Morus025784.p1	Phospholipase D alpha 1	12	264	0.30	92059	lipid metabolism
180	Morus011120.p1	Pectinesterase/pectinesterase inhibitor 39	20	124	0.29	62063	cell wall
181	Morus027934.p1	Alpha-L-arabinofuranosidase 1	9	230	0.29	69049	cell wall
182	Morus001243.p1	Hypothetical protein	9	68	0.29	18600	not assigned
183	Morus018550.p1	Glycine-rich RNA-binding protein	7	98	0.29	18416	RNA
184	Morus025788.p1	Conserved hypothetical protein	3	51	0.29	18479	development
185	Morus002798.p1	Fructokinase-5	5	86	0.29	36855	major CHO metabolism
186	Morus014011.p1	Glycerophosphoryl diester phosphodiesterase 2	22	247	0.29	81816	lipid metabolism
187	Morus014029.p1	Cell division protein	6	165	0.29	44620	cell
188	Morus005375.p1	Blue copper protein	3	42	0.28	18851	not assigned
189	Morus019087.p1	Mitochondrial 2-oxoglutarate/malate carrier protein	8	169	0.28	32224	transport
190	Morus011731.p1	STS14 protein	2	68	0.28	19321	stress

191	Morus012122.p1	Polyphenol oxidase, chloroplastic	8	175	0.27	65528	protein
192	Morus002874.p1	Leucine aminopeptidase 3, chloroplastic	14	308	0.27	60563	protein
193	Morus013333.p1	Specific protein TSJT1	2	43	0.27	27206	hormone metabolism
194	Morus009084.p1	Desiccation-related protein PCC13-62	3	34	0.26	34144	stress
195	Morus010230.p1	Superoxide dismutase [Cu-Zn]	2	43	0.26	20420	redox
196	Morus019413.p1	Cysteine proteinase 15A	8	227	0.26	41574	protein
197	Morus020958.p1	Lamin-like protein	9	117	0.26	20403	miscellaneous
198	Morus001864.p1	Hypothetical protein	4	103	0.26	20449	protein
199	Morus019523.p1	Far uphotosynthesistream element-binding protein 1	10	202	0.25	71479	RNA
200	Morus001308.p1	Disease resistance response protein	2	56	0.25	21320	stress
201	Morus025843.p1	Fructose-bisphosphate aldolase 3, chloroplastic	5	172	0.25	42615	photosynthesis
202	Morus008025.p1	ATP synthase subunit delta, mitochondrial	6	155	0.25	21494	mitochondrial electron transport
203	Morus012965.p1	Predicted protein	4	49	0.25	21111	development
204	Morus003380.p1	Beta-fructofuranosidase	7	84	0.25	72627	major CHO metabolism
205	Morus021774.p1	Expansin-B2	6	45	0.25	29266	cell wall
206	Morus016343.p1	Hypothetical protein	11	53	0.24	79098	not assigned
207	Morus004410.p1	DNA repair protein RAD23-1	7	212	0.24	45167	DNA
208	Morus020384.p1	Cysteine synthase	5	106	0.24	43997	amino acid metabolism
209	Morus015818.p1	Glucan endo-1,3-beta-glucosidase A6	12	134	0.24	52145	miscellaneous
210	Morus000168.p1	Uncharacterized oxidoreductase	11	79	0.24	36852	OPP
211	Morus003990.p1	Cysteine-rich repeat secretory protein 12	2	69	0.24	37585	signalling
212	Morus023984.p1	NAD(P)H:quinone oxidoreductase	9	64	0.24	37824	not assigned
213	Morus024573.p1	Hevein-like protein	2	74	0.23	23166	stress
214	Morus000761.p1	Pentatricopeptide repeat-containing protein	19	233	0.23	92406	not assigned
215	Morus011230.p1	Alpha-galactosidase	7	100	0.23	46358	minor CHO metabolism
216	Morus022986.p1	Cathepsin B	4	104	0.23	38578	protein
217	Morus026631.p1	Conserved hypothetical protein	4	147	0.23	23352	not assigned
218	Morus020153.p1	Pathogenesis-related protein 5	5	96	0.22	32013	stress
219	Morus017748.p1	Plastid-lipid-associated protein 3, chloroplastic	15	116	0.22	40414	cell
220	Morus007643.p1	Protein PPLZ12	5	49	0.22	31999	not assigned
221	Morus021189.p1	Hypothetical protein	4	74	0.22	23480	not assigned
222	Morus025612.p1	Inactive receptor kinase	9	147	0.22	72471	signalling

223	Morus015771.p1	Membrane steroid-binding protein 2	2	63	0.22	24221	redox
224	Morus017264.p1	Chalcone-flavonone isomerase	4	106	0.22	24290	secondary metabolism
225	Morus024594.p1	Unknown	6	248	0.22	24078	not assigned
226	Morus026294.p1	Acid phosphatase 1	4	137	0.22	24323	miscellaneous
227	Morus013255.p1	Late embryogenesis abundant protein D-11	2	72	0.22	23457	stress
228	Morus013361.p1	Protein disulfide-isomerase	8	96	0.22	56492	redox
229	Morus024614.p1	Heme-binding protein 2	6	167	0.21	25414	tetrapyrrole synthesis
230	Morus004394.p1	Miraculin	11	331	0.21	24635	stress
231	Morus026245.p1	Aquaporin TIP2-1	2	58	0.21	25221	transport
232	Morus019552.p1	Sucrose synthase	12	200	0.21	92842	major CHO metabolism
233	Morus002597.p1	Nascent polypeptide-associated complex subunit alpha-like protein 2	2	67	0.21	24462	protein
234	Morus002210.p1	LL-diaminopimelate aminotransferase, chloroplastic	6	81	0.21	50847	amino acid metabolism
235	Morus009247.p1	ATP-citrate synthase	6	156	0.21	66507	TCA
236	Morus020380.p1	60S ribosomal protein L5	4	125	0.20	35074	protein
237	Morus002328.p1	Glucan endo-1,3-beta-glucosidase 4	6	187	0.20	53492	miscellaneous
238	Morus012993.p1	UPF0308 protein	3	48	0.20	26190	not assigned
239	Morus011198.p1	L-ascorbate peroxidase, cytosolic	5	65	0.19	27414	redox
240	Morus003374.p1	Uncharacterized protein	2	33	0.19	26692	not assigned
241	Morus013581.p1	Expansin-A10	8	43	0.19	27507	cell wall
242	Morus024082.p1	3-isopropylmalate dehydratase small subunit	2	153	0.19	26695	not assigned
243	Morus023079.p1	Receptor-like protein kinase	8	143	0.19	64907	stress
244	Morus007114.p1	Glycine-rich RNA-binding protein 2	4	152	0.19	27802	RNA
245	Morus004471.p1	60S ribosomal protein L3	9	47	0.18	47228	protein
246	Morus008123.p1	IAA-amino acid hydrolase	4	82	0.18	47707	hormone metabolism
247	Morus024851.p1	Catalase isozyme 1	10	49	0.18	57208	redox
248	Morus018045.p1	Glucan endo-1,3-beta-glucosidase 1	9	180	0.18	57055	not assigned
249	Morus008306.p1	Patellin-3	5	75	0.18	58217	transport
250	Morus013470.p1	Histone H1	16	31	0.18	29133	DNA
251	Morus019101.p1	Patatin group M-2	6	77	0.18	48408	development
252	Morus008205.p1	Proteasomal ubiquitin receptor ADRM1	5	92	0.18	28976	not assigned
253	Morus002856.p1	Chlorophyll a-b binding protein 40, chloroplastic	5	76	0.18	28149	photosynthesis
254	Morus024555.p1	Poly(rC)-binding protein 3	5	168	0.18	48844	RNA

255	Morus018282.p1	Cysteine-rich repeat secretory protein 38	2	52	0.18	27945	signalling
256	Morus004151.p1	Protein grpE	7	73	0.18	57585	protein
257	Morus014380.p1	LysM domain-containing GPI-anchored protein 2	5	94	0.18	39401	not assigned
258	Morus014761.p1	Arginine biosynthesis bifunctional protein	4	50	0.18	40125	amino acid metabolism
259	Morus017351.p1	Serine carboxypeptidase-like 50	6	84	0.17	49604	protein
260	Morus004905.p1	Polygalacturonase QRT3	5	50	0.17	52230	not assigned
261	Morus009128.p1	Endo-1,3(4)-beta-glucanase 1	11	178	0.17	83842	stress
262	Morus026664.p1	26S proteasome non-ATPase regulatory subunit 4	11	83	0.16	43012	protein
263	Morus014426.p1	Conserved hypothetical protein	2	146	0.16	32568	development
264	Morus016969.p1	Uracil phosphoribosyltransferase	2	31	0.16	32116	nucleotide metabolism
265	Morus017496.p1	Pyruvate dehydrogenase	4	122	0.16	32244	TCA
266	Morus014390.p1	Fasciclin-like arabinogalactan protein 12	14	295	0.16	31735	cell wall
267	Morus002842.p1	Glucan endo-1,3-beta-glucosidase 8	3	69	0.16	54713	miscellaneous
268	Morus003849.p1	60S ribosomal protein L4	7	104	0.15	44987	protein
269	Morus013569.p1	Uncharacterized protein	2	41	0.15	44717	not assigned
270	Morus025732.p1	Pyrophosphate-energized vacuolar membrane proton pump 1	10	95	0.15	80999	transport
271	Morus014667.p1	Alpha-xylosidase	17	43	0.15	103539	miscellaneous
272	Morus024951.p1	Triosephosphate isomerase, chloroplastic	4	76	0.15	34813	photosynthesis
273	Morus025594.p1	Pectinesterase 2	4	91	0.15	57091	cell wall
274	Morus006170.p1	DNA-damage-repair/toleration protein DRT102	9	121	0.15	33933	DNA
275	Morus018207.p1	Transmembrane emp24 domain-containing protein	5	188	0.15	33455	protein
276	Morus002253.p1	Polyadenylate-binding protein 2	8	72	0.15	70009	RNA
277	Morus011044.p1	PI-PLC X domain	3	155	0.15	45428	signalling
278	Morus017174.p1	Predicted protein	6	104	0.15	33060	signalling
279	Morus009108.p1	Uncharacterized protein	5	74	0.15	47451	cell wall
280	Morus016271.p1	Elongation factor 2	8	44	0.14	99403	protein
281	Morus008661.p1	14-3-3-like protein A	6	129	0.14	81889	cell
282	Morus013471.p1	Eukaryotic initiation factor 4A-8	13	98	0.14	48351	protein
283	Morus017833.p1	RNA-binding protein C23E6.01c	8	101	0.14	60390	RNA
284	Morus001657.p1	6-phosphogluconolactonase 4, chloroplastic	5	128	0.14	35151	OPP
285	Morus008646.p1	DNA repair protein RAD23-3	6	30	0.14	59703	DNA
286	Morus009365.p1	5-methyltetrahydropteroyltriglutamate-homocysteine methyltransferase	4	61	0.14	84904	amino acid metabolism

287	Morus016376.p1	Dihydrolipoyllysin	5	72	0.14	50682	TCA
288	Morus000860.p1	Glucan endo-1,3-beta-glucosidase 3	8	56	0.14	50508	miscellaneous
289	Morus003338.p1	Glucan endo-1,3-beta-glucosidase 7	12	49	0.14	49924	not assigned
290	Morus007784.p1	UTP-glucose-1-phosphate uridylyltransferase	8	54	0.13	76133	glycolysis
291	Morus013374.p1	Proteasome subunit beta type-3-A	5	191	0.13	80974	protein
292	Morus013723.p1	Homoserine kinase	2	77	0.13	38585	amino acid metabolism
293	Morus001906.p1	Probable protein disulfide-isomerase A6	4	48	0.13	40439	redox
294	Morus008766.p1	Serine/arginine repetitive matrix protein 2	2	79	0.13	37780	not assigned
295	Morus000879.p1	RING finger protein 126	5	56	0.13	40372	protein
296	Morus005419.p1	Late embryogenesis abundant protein D-34	6	122	0.13	37985	development
297	Morus014256.p1	Fasciclin-like arabinogalactan protein 2	2	119	0.13	40127	cell wall
298	Morus017695.p1	31 kDa ribonucleoprotein, chloroplastic	7	79	0.13	38128	RNA
299	Morus021899.p1	Beta-galactosidase 3	15	388	0.13	92114	miscellaneous
300	Morus010889.p1	NADP-dependent malic enzyme	15	214	0.13	113884	TCA
301	Morus027393.p1	Thiol protease aleurain	2	139	0.13	39623	protein
302	Morus013818.p1	Cytochrome c1-1	6	76	0.12	69151	mitochondrial electron transport
303	Morus014182.p1	Aspartic proteinase	9	138	0.12	56367	protein
304	Morus011342.p1	3,4-dihydroxy-2-butanone kinase	13	93	0.12	56500	minor CHO metabolism
305	Morus027840.p1	Metalloendoproteinase 1	9	35	0.12	41640	protein
306	Morus025123.p1	Glutamate decarboxylase	7	45	0.12	56571	amino acid metabolism
307	Morus018412.p1	Enoyl-[acyl-carrier-protein] reductase	6	51	0.12	41362	lipid metabolism
308	Morus012393.p1	Cysteine synthase, chloroplastic/chromoplastic	4	94	0.12	40471	amino acid metabolism
309	Morus022978.p1	Chitotriosidase-1	2	43	0.12	40642	stress
310	Morus006986.p1	Glucan endo-1,3-beta-glucosidase 13	27	380	0.12	152348	miscellaneous
311	Morus008304.p1	Patellin-2	7	46	0.12	72014	transport
312	Morus027754.p1	Polygalacturonase	6	79	0.11	58616	cell wall
313	Morus020752.p1	Ribokinase	6	72	0.11	45949	minor CHO metabolism
314	Morus011664.p1	Multicopper oxidase	6	50	0.11	60522	not assigned
315	Morus007494.p1	RuBisCO large subunit	11	63	0.11	62000	photosynthesis
316	Morus005412.p1	Eukaryotic translation initiation factor 6	8	36	0.11	45510	protein
317	Morus005944.p1	Heterogeneous nuclear ribonucleoprotein A3 homolog 1	2	102	0.11	43839	RNA
318	Morus024157.p1	60S ribosomal protein	8	81	0.11	65238	protein

319	Morus022863.p1	(R)-mandelonitrile lyase 1	9	96	0.11	76101	miscellaneous
320	Morus006060.p1	V-type proton ATPase subunit B2	5	122	0.11	63333	transport
321	Morus004111.p1	Calreticulin	6	80	0.10	50196	signalling
322	Morus014786.p1	Methylmalonate-semialdehyde dehydrogenase	22	159	0.10	105060	amino acid metabolism
323	Morus006120.p1	ABC transporter A family member 7	10	180	0.10	102773	transport
324	Morus013778.p1	Monodehydroascorbate reductase	6	82	0.10	49982	redox
325	Morus009515.p1	Mucin-5AC (Fragments)	4	80	0.10	48196	stress
326	Morus024141.p1	Beta-D-xylosidase 4	2	124	0.10	84604	cell wall
327	Morus027185.p1	Glucan endo-1,3-beta-glucosidase 2	3	53	0.09	53950	miscellaneous
328	Morus026092.p1	Serine carboxypeptidase II-3	4	106	0.09	53533	protein
329	Morus018991.p1	Flavonoid 3,5-hydroxylase	6	99	0.09	92734	secondary metabolism
330	Morus007961.p1	Hypothetical protein	13	171	0.09	95561	not assigned
331	Morus007672.p1	Beta-galactosidase 8	4	58	0.08	98498	miscellaneous
332	Morus012896.p1	Pectinesterase/pectinesterase inhibitor	12	45	0.08	60870	cell wall
333	Morus024423.p1	Formin-like protein 20	8	30	0.08	85748	cell wall
334	Morus010734.p1	NADP-dependent malic enzyme	9	62	0.08	81998	TCA
335	Morus005110.p1	Beta-galactosidase 17	4	46	0.07	75256	miscellaneous
336	Morus024018.p1	Methylglutaconyl-CoA hydratase, mitochondrial	7	51	0.07	71049	protein
337	Morus025925.p1	Alpha-glucosidase	4	44	0.07	93365	miscellaneous
338	Morus006857.p1	Laccase-15	7	31	0.07	67126	secondary metabolism
339	Morus006939.p1	Cytosolic endo-beta-N-acetylglucosaminidase	4	30	0.06	77642	not assigned
340	Morus020837.p1	Extensin	8	32	0.06	84300	cell wall
341	Morus027199.p1	Valyl-tRNA synthetase	10	33	0.06	80902	protein
342	Morus000633.p1	GDSL esterase/lipase 1	13	43	0.06	86650	miscellaneous
343	Morus023775.p1	SEC14 cytosolic factor	2	50	0.05	91332	transport
344	Morus013835.p1	Vacuolar-processing enzyme	8	105	0.05	103224	protein
345	Morus008787.p1	Beta-galactosidase 9	9	77	0.05	106501	miscellaneous
346	Morus017204.p1	Copper-transporting ATPase	6	127	0.05	101020	transport
347	Morus008686.p1	Lysosomal alpha-mannosidase	6	54	0.05	102857	miscellaneous
348	Morus003274.p1	Sucrose synthase 2	7	48	0.05	91853	major CHO metabolism
349	Morus013674.p1	Receptor-like protein	5	30	0.05	101469	signalling
350	Morus001973.p1	Nuclear transcription factor Y subunit C-9	3	63	0.04	109717	transport

351	Morus006546.p1	Clathrin heavy chain	13	31	0.03	201524	cell
352	Morus027728.p1	Alpha-glucan water dikinase, chloroplastic	16	73	0.03	161084	major CHO metabolism
353	Morus006400.p1	HIPL1 protein	7	110	0.03	157277	cell
354	Morus006573.p1	Niemann-Pick C1 protein	4	77	0.03	144117	not assigned
355	Morus008195.p1	Pentatricopeptide repeat	15	58	0.03	169968	DNA

[a] Protein ID, according to the Morus database; [b] M.P., number of matched peptides; [c] Mol (%), protein abundance; [d] Function, function categorized using MapMan bin codes; protein, protein synthesis/folding/degradation/posttranslational; cell, cell organization/cycle; RNA, RNA regulation of transcription; redox, redox ascorbate/glutathione metabolism; OPP, oxidative pentose phosphate; and TCA, tricarboxylic acid cycle.

Supplemental Table S4. Contents of total flavonoids in different organs of *Morus.*

	Leaf	Branch	Root
Total flavonoids * (mg QE/g DW)	14.23±0.13	5.63±0.21	23.79±0.09

* The values are expressed as mean±SD (n=3). QE, quercetin-3-O-rutinoside equivalents; DW, dry weight.

Supplemental table S5. Primers of three genes used in this study.

**Gene**	**Sense Primer**	**Anti-Sense Primer**
β-actin	AGGGGAAGCTGGCTTATGTT	CGGGCAGCTCATAGTTCTTC
Chalcone isomerase	TCACCTCCGCCATTGCCTTA	CGCCACCAAGAAACAGAGTCT
Phosphoglycerate kinase	GCAACTGAATCACCTCCTCCGA	GCCAGCATCCAGCATTCCAGAT
Isoflavonoid reductase	CGTATGTGTCGTGGAACCTGTT	ATGTTGTCTGCTGGCTTGATGT
